# Unveiling polyphenol-protein interactions: a comprehensive computational analysis

**DOI:** 10.1186/s13321-025-00997-3

**Published:** 2025-04-10

**Authors:** Samo Lešnik, Marko Jukić, Urban Bren

**Affiliations:** 1 Laboratory of Physical Chemistry and Chemical Thermodynamics, Faculty of Chemistry and Chemical Engineering, University of Maribor, Smetanova 17, 2000 Maribor, Slovenia; 2 IOS, Institute of Environmental Protection and Sensors, Beloruska 7, 2000 Maribor, Slovenia; 3 Faculty of Mathematics, Natural Sciences and Information Technologies, University of Primorska, Glagoljaška 8, 6000 Koper, Slovenia

**Keywords:** Polyphenols, Polyphenol-protein complexes, Molecular dynamics simulations, Noncovalent interactions, Water-mediated interactions, Glycosylation, Dynamic behavior

## Abstract

Our study investigates polyphenol-protein interactions, analyzing their structural diversity and dynamic behavior. Analysis of the entire Protein Data Bank reveals diverse polyphenolic structures, engaging in various noncovalent interactions with proteins. Interactions observed across crystal structures among diverse polyphenolic classes reveal similarities, underscoring consistent patterns across a spectrum of structural motifs. On the other hand, molecular dynamics (MD) simulations of polyphenol-protein complexes unveil dynamic binding patterns, highlighting the influx of water molecules into the binding site and underscoring limitations of static crystal structures. Water-mediated interactions emerge as crucial in polyphenol-protein binding, leading to variable binding patterns observed in MD simulations. Comparison of high- and low-resolution crystal structures as starting points for MD simulations demonstrates their robustness, exhibiting consistent dynamics regardless of the quality of the initial structural data. Additionally, the impact of glycosylation on polyphenol binding is explored, revealing its role in modulating interactions with proteins. In contrast to synthetic drugs, polyphenol binding seems to exhibit heightened flexibility, driven by dynamic water-mediated interactions, which may also facilitate their promiscuous binding. Comprehensive dynamic studies are, therefore essential to understand polyphenol-protein recognition mechanisms. Overall, our study provides novel insights into polyphenol-protein interactions, informing future research for harnessing polyphenolic therapeutic potential through rational drug design.

**Scientific contribution**: In this study, we present an analysis of (natural) polyphenol-protein binding conformations, leveraging the entirety of the Protein Data Bank structural data on polyphenols, while extending the binding conformation sampling through molecular dynamics simulations. For the first time, we introduce experimentally supported large-scale systematization of polyphenol binding patterns. Moreover, our insight into the significance of explicit water molecules and hydrogen-bond bridging rationalizes the polyphenol promiscuity paradigm, advocating for a deeper understanding of polyphenol recognition mechanisms crucial for informed natural compound-based drug design.

**Supplementary Information:**

The online version contains supplementary material available at 10.1186/s13321-025-00997-3.

## Introduction

Polyphenols, classified as secondary plant metabolites, are ubiquitously present in a wide range of food sources, including vegetables, fruits, grains, and various beverages [1]. Nevertheless, the scientific literature often grapples with the precise definition and chemical structure of polyphenols, leading to some ambiguity in their characterization [2]. Strictly speaking, polyphenols consist of one or more aromatic rings adorned with hydroxyl group(s). Despite this shared characteristic, they encompass a wide array of molecules with diverse chemical structures. Growing evidence underscores the significance of polyphenols for human health, attributing to them antioxidant, anti-inflammatory, and anticarcinogenic properties, as well as with protective effects against metabolic disorders and chronic diseases [3].

Numerous benefits associated with polyphenols concerning human health have historically been ascribed to their antioxidant properties [4, 5]. However, contemporary perspectives have shifted away from this hypothesis, as compelling evidence now supports the idea that polyphenols can exert their effects through specific interactions with protein targets, irrespective of their redox properties [6, 7]. These interactions, in turn, enable polyphenols to modulate signalling and metabolic pathways implicated in various diseases [8, 9]. Moreover, recent findings provide evidence that polyphenols can interact with protein targets within microorganisms, endowing polyphenols with antimicrobial properties. Notably, their ability to inhibit crucial viral [10–12], bacterial [13], or fungal [14] enzymes highlights their potential in combating microbial threats. The multifaceted interactions with both human and microbial proteins position polyphenols as promising bioactive compounds with diverse therapeutic implications.

In this study, our objective was to leverage the wealth of information within the Protein Data Bank (PDB) database [15] to discern the intricacies of polyphenolic interactions within protein binding sites. By doing so, we aimed to identify a characteristic set of interactions that each class of polyphenols establishes with proteins. This endeavour tried to shed light on the well-documented promiscuity of polyphenols, a phenomenon widely acknowledged but whose precise molecular mechanisms remain largely unexplored.

**Fig. 1:**
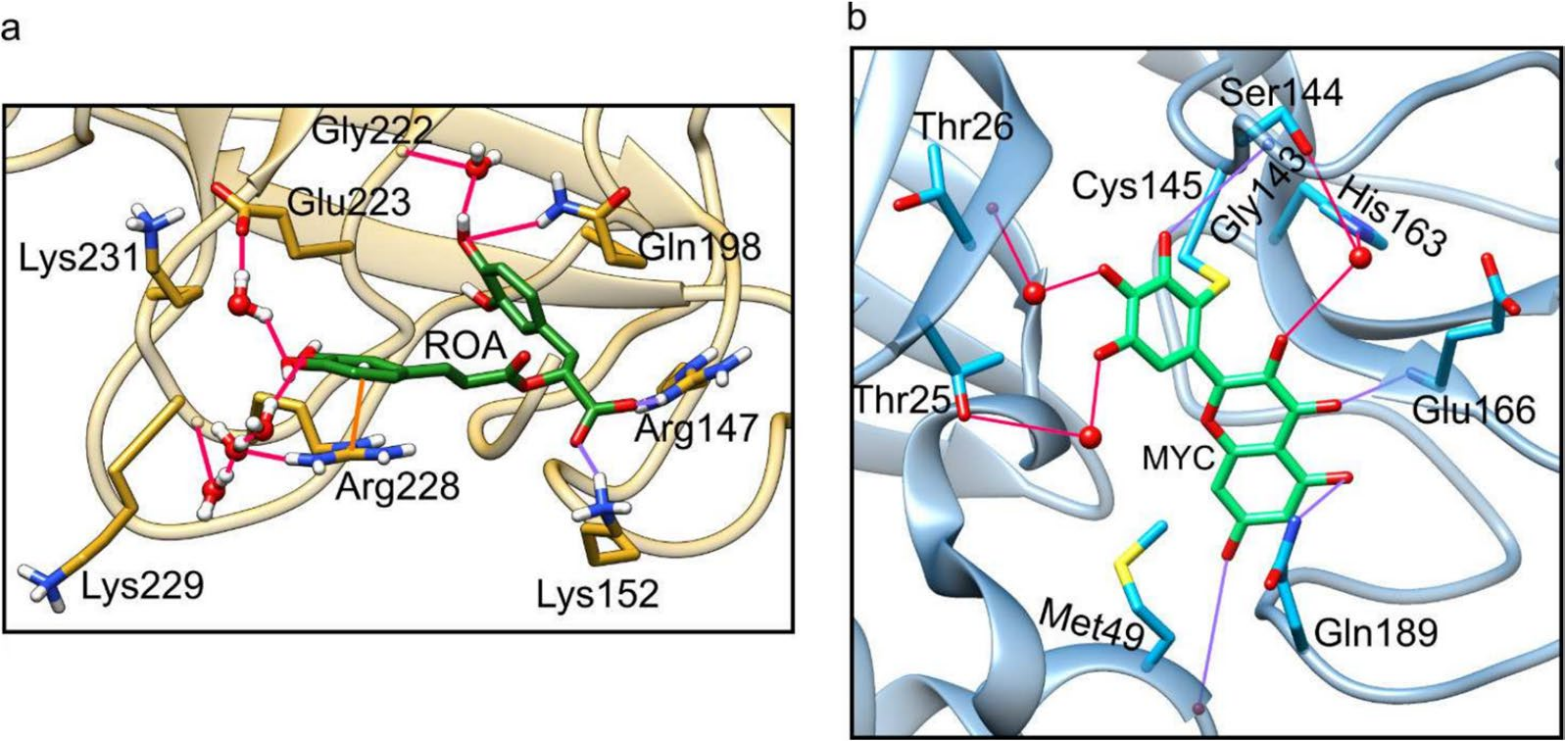
Polyphenols form water-mediated H-bonds in static as well as dynamic structures. **a** A prototypical snapshot from an MD simulation where rosmarinic acid (ROA, carbons denoted as dark-green sticks) binds to the factor X enzyme [16]. We observe several water-mediated H-bond bridges (red lines) within the active binding site that stabilize ROA binding. **b** The crystal structure (PDB ID: 7B3E) of flavonoid myricetin (MYC, carbons denoted as light-green sticks) covalently bound to the severe acute respiratory syndrome coronavirus 2 (SARS-CoV-2) main protease. MYC forms several water-mediated H-bond bridges (red lines) within the binding site. MYC also forms direct H-bonds, which are shown as purple lines

Building upon our prior investigations, wherein extensive molecular dynamics (MD) simulations were conducted on three distinct protein systems binding polyphenols extracted from rosemary (carnosic acid, carnosol, rosmanol, and rosmarinic acid), we made a notable observation regarding the pivotal role played by water molecules in stabilizing their binding [16] (Fig. 1a). It became evident that the investigated polyphenols formed a significant number of hydrogen bonds with water molecules, and our findings underscored the significance of water-mediated interactions in the intricate interplay between polyphenols and proteins. We hypothesized that the inherent tendency of polyphenols to engage in interactions between their numerous hydroxyl groups and conserved water molecules within protein binding sites of diverse configurations could offer a plausible explanation for their promiscuous binding behavior. This observation gains additional support from the prevalence of water-mediated H-bond interactions between polyphenols and amino-acid residues, as evidenced by numerous high-resolution structures in the PDB. A case in point represents the recently published high-resolution structure of the severe acute respiratory syndrome coronavirus 2 (SARS-CoV-2) main protease, where flavonoid myricetin binds to the protease binding site through several water-mediated H-bond bridges (Fig. 1b) [12]. Similar water-mediated interactions are present in additional structures such as resveratrol-3-O-glucuronide bound to transthyretin [17], rosmarinic acid bound to myotoxin II [18] or catechol bound to urease [19].

The utility of high-resolution protein structures is emphasized in this context, as structures solved at lower resolutions often lack structural waters and may underestimate the internal hydrogen bond networks of proteins [20]. Given the relative scarcity of high-resolution structures involving polyphenols bound to proteins in the PDB, in silico approaches are essential for locating bridging water molecules. An example of such an approach represents MADE (and ProBiS H2O) applications, which have been developed to identify conserved water molecules in macromolecular systems [21, 22]. The MADE workflow scans available experimental PDB data to identify binding sites structurally similar to the binding site of the query protein. These identified similar binding sites are then superimposed, facilitating a transfer of water molecules within such sites to the query protein. The resulting water location data is clustered to identify discrete spaces exhibiting a high conservation of water molecules, providing a powerful visualization tool in the context of the studied protein system. MADE thus represents a rapid method that harnesses existing experimental data to place conserved water molecules within protein binding sites.

Moreover, as already emphasized earlier, the inclusion of explicit water molecules in MD simulations is imperative when investigating water-mediated polyphenolic interactions. This approach proves essential in discerning potential bridging water molecules that play a pivotal role in polyphenolic binding [13, 18].

The focus of this work is directed towards globular proteins characterized by well-defined binding cavities, notably enzymes and receptors, where strong binding constants and specific interactions between polyphenols and proteins can be anticipated [23–25]. This stands in contrast to conformationally open proteins with multiple binding sites, exemplified by proline-rich salivary proteins, where small polyphenols can be expected to exhibit a weak binding.

In the initial phase of our study, we meticulously examined polyphenol-protein interactions across the entire PDB, categorizing polyphenoles into distinct classes for a systematic analysis. Exploration of the entire PDB revealed a wide array of polyphenolic structures, each engaging in noncovalent interactions with proteins. Across crystal structures representing different classes of polyphenols, we observed common interaction patterns, indicating a consistent behavior across various structural motifs.

However, subsequent extensive MD simulations of polyphenol-protein complexes uncover dynamic binding patterns, emphasizing the influx of water molecules into the binding sites and exposing the limitations of static crystal structures. Notably, water-mediated interactions emerge as pivotal in polyphenol-protein binding, contributing to the variable binding patterns observed in MD simulations. Moreover, comparing high- and low-resolution crystal structures as initial points of MD simulations demonstrates their robustness. Furthermore, we explored the influence of glycosylation on polyphenol binding, shedding light on its role in modulating interactions with proteins.

The work presented here marks the initial phase of a comprehensive project aimed at constructing a database delineating polyphenol-protein interaction profiles, utilizing known structures deposited in the PDB. An establishment of such a database holds immense value for future endeavors in target identification and drug design. Specifically, it provides a practical means of validating pose predictions derived from classical- and inverse-docking procedures. If a given docking pose of a polyphenol aligns with the interactions identified in this study for a particular polyphenolic class (e.g., stilbenes), there is an enhanced confidence in the accuracy of such a pose.

Moreover, by harnessing existing polyphenol-protein interaction data, we aspire to formulate in the future a knowledge-based scoring function tailored to polyphenolic structures. This scoring function would systematically capture specific interactions within a queried protein binding site, considering distances between specific atom types. The scoring mechanism would be informed by existing polyphenol-protein interactions observed in crystal structures deposited in the PDB. Such an approach promises to enhance the precision and reliability of scoring in the context of polyphenol-protein interactions, thereby contributing to the advancement of rational natural-drug design strategies.

## Methods

### Mining publicly available databases

To identify protein structures within the PDB that bind polyphenols, we initiated the process by retrieving a comprehensive list of polyphenols from the Phenol-Explorer online database [26–28], which encompasses approximately 500 polyphenols and around 380 metabolites identified in biofluids following the consumption of polyphenol-rich sources. To compile a thorough catalog of polyphenol-protein interactions, we utilized the list of corresponding Simplified Molecular Input Line Entry System (SMILES) strings for polyphenols (including their metabolites) from the PhenolExplorer to query the entire PDB.

**Fig. 2:**
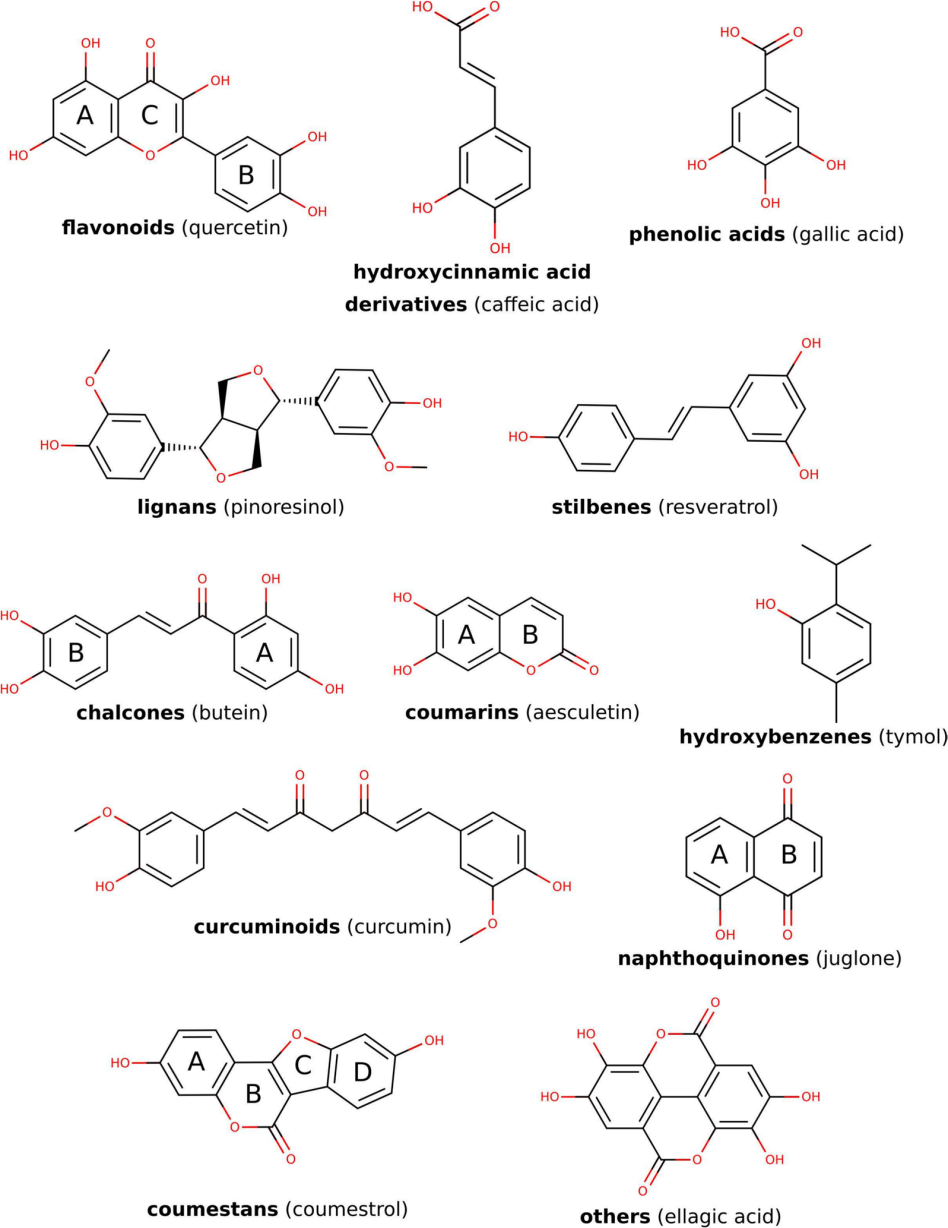
Our classification of polyphenols known to bind to proteins sourced from the PDB, with each class represented by a prototypical compound

Employing OpenBabel [29] each polyphenol SMILES string was systematically compared to the entirety of ligands deposited in the PDB (PDB database obtained on September 1st 2023), generating Tanimoto-expressed similarities based on FP2 fingerprints. We then extracted each PDB structure containing a ligand that exhibited a Tanimoto coefficient of 0.90 or higher. Subsequently, based on its molecular structure, each polyphenol was manually classified into one of the 12 distinct classes, as depicted in Fig. 2.

Following the procedure outlined for identifying ligands with Tanimoto coefficients of $\ge$ 0.90, a manual curation step was implemented. Structures deemed excessively simple, including phenol and benzoic acid, were removed. On the other hand, certain structures that did not fit the strict definition of polyphenols were retained. Notably, cinnamic acid was retained due to its widespread occurrence, recognized significance in plant-based medicine, and due to its role as a parent compound for other essential hydroxycinnamic derivatives such as caffeic acid [30, 31]. Similarly, specific monoterpenes like thymol or carvacrol were also retained for identical reasons. Compounds featuring aromatic ether moieties in lieu of hydroxy groups were also preserved.

The final database comprises of 939 entries from the PDB first biological assemblies (Supporting Information Table S1). Notably, each alternative conformation, when present, is considered a separate entry, resulting in a total of 1431 structures. Within these protein structures, 193 unique polyphenolic ligands have been identified. We emphasize that polyphenols containing covalent interactions with the protein targets were also retained in our study, as covalently bound polyphenols still maintain noncovalent interactions, which typically facilitate initial recognition and binding events [32].

### Interaction identification and analysis

#### Interaction analysis of variable types using PLIP

Each polyphenol-ligand complex from the PDB underwent analyses using the automated version of the Protein-Ligand Interaction Profiler (PLIP) algorithm [33, 34]. PLIP employs a meticulous approach to identify major noncovalent interactions at the single-atom level between small molecules and proteins. The algorithm detects seven interaction types: hydrogen bonds, hydrophobic contacts, $\uppi$-stacking, $\uppi$-cation interactions, salt bridges, water bridges, and metal complexes. It is worth noting that PLIP also identifies halogen bonds; however, they are not expected to be present in polyphenols and are not discussed in this paper.

Before identifying interactions, the input structure undergoes hydrogenation, and the ligand is extracted along with its binding site. Subsequently, the algorithm characterizes ligand atoms and functional groups by discerning hydrophobic regions and identifying acceptor/donor functional groups crucial for hydrogen bonds. Moreover, PLIP identifies aromatic rings and charge centers, essential for the formation of $\uppi$-stacking, $\uppi$-cation interactions, or salt bridges.

The interaction profile of each polyphenol-protein complex will be discussed based on the classification of the polyphenol itself (e.g., flavonoid, stilbene, etc.), presented in Fig. 2. This approach streamlines the identification of possible characteristic interactions that each polyphenolic class can establish with proteins.

Additionally, PLIP was utilized to generate time-dependent interaction contact maps through an in-house Python script that executed the PLIP analysis on each frame of the MD simulations.

#### Atom type classification and radial distribution analysis of polyphenol-protein interactions

To facilitate the future development of a polyphenol-specific scoring function, each heavy atom and polar hydrogen of the polyphenol and protein was also categorized into specific types, reflecting their topology and hybridization states (e.g., C.3-sp^3^-hybridized carbon, O.2-sp^2^-hybridized oxygen, etc.). For each polyphenolic atom, we tallied the number of ligand and receptor atom types in contact with each other (e.g., the hydroxyl O.3 of the polyphenol and the N.am of the Gln sidechain). Close intermolecular interactions between specific atom types that occur more frequently than expected in a random distribution are likely to be energetically favorable and, therefore, contribute positively to the binding affinity within the scoring function [35]. The key advantage of such knowledge-based potentials, to be developed in the future, lies in their ability to circumvent the need to balance multiple opposing contributions to the binding, such as desolvation and entropy, as these factors are treated implicitly.

The atom types used for this purpose are defined in Supporting Information Table S2 and are based on Tripos SYBYL mol2 atom types, with additional discrimination based on the location of the atom within the binding site (e.g., protein backbone, sidechain, or cofactor/ion/water) [36]. The interactions were evaluated using pair distribution function $g_{i,j}(r)$ with normalization based on the total number of pair observations and the volume of shells corresponding to each bin of the histogram:

1 $$\begin{aligned} g_{i,j}(r) = \frac{p_{i,j}(r)}{4 \pi r^2bN} - g_{ab}(r), \end{aligned}$$

where $p_{i,j}$ is the occurrence of atom type pairs *i* and *j* within each histogram bin, *N* is the total occurrence of protein-polyphenol atom pairs within a 7.5 Å distance, *r* is the average distance value for each histogram bin, and *b* is the width of the histogram interval. The term $g_{ab}(r)$ represents the normalized radial distribution between all polyphenol-protein atom pairs, independent of their type.

Normalizing with the shell volumes $4 \pi r^2b$ and with the radial distribution of all atom types $g_{ab}(r)$ eliminates the “non-interacting” background distribution from the protein-polyphenol systems, facilitating a faster convergence to zero at large distances [36–38]. The selected maximal distance of 7.5 Å aligns with the default cutoff of PLIP [33, 34].

### Molecular dynamics

To comprehensively understand the impact of polyphenol binding, it is essential to consider the conformational changes experienced by the protein targets. To attain an atomistic view of how polyphenols interact with proteins and influence their structural dynamics, we conducted MD simulations on a carefully selected subset of protein PDB structures bound to polyphenols.

We specifically observed how the binding patterns evolved during MD simulations, providing insights into the stability of polyphenolic poses within the protein binding site. This approach is particularly crucial for identifying water-mediated H-bond bridges, given their well-known transient nature [39–41].

The protein-ligand complexes were prepared for subsequent MD simulations using the Chemistry at Harvard Macromolecular Mechanics graphical user interface (CHARMM-GUI) [42], utilizing structures obtained from the PDB. Prior to commencing MD simulations, the complexes underwent solvation in rectangular TIP3P water boxes (with a 15 Å padding using periodic boundary conditions) including 0.15 M NaCl. To maintain system electro neutrality, the appropriate number of Na^+^ or Cl^−^ ions was added. Protonation states of ionizable amino-acid residues followed standard conventions in Chemistry at Harvard Macromolecular Mechanics (CHARMM), where Asp/Glu residues are negatively charged, Arg/Lys residues are positively charged, and His residues are singly protonated at the N1$\updelta$ atom. The CHARMM36 forcefield parameters were employed for proteins [43, 44], augmented by the CHARMM36-WYF set to enhance the description of $\uppi$-cation interactions [45]. Forcefield parameters for all polyphenols were developed using the automated ParamChem web server [46]. While acknowledging the limitations of automated methods in parameter determination for drug-like small molecules, our decision was informed by the low reported nonbonded penalties for most sterically accessible ligand atoms and by the relatively low penalties for bonded interactions corresponding to flexible moieties, thus providing confidence in the suitability of developed parameters for our study.

The coordinate files of polyphenols, proteins and water molecules were combined, and 50 steps of steepest descent and 50 steps of adopted basis Newton–Raphson energy minimization were carried out to remove any potential steric clashes that may occur, as well as to optimize the atomic coordinates of the complexes. The complex was then equilibrated using NAMD [47, 48] at 310.15 K using the HOOVER thermostat and an integration timestep of 1 fs during a brief MD simulation. The NVT ensemble’s (constant number of particles, volume and temperature) equilibration molecular dynamics took 0.125 ns to complete. This was followed by two independent production runs (the main and replica simulations) of 1 $\upmu\mathrm{s}$, again performed using NAMD. Production runs were carried out in the NPT ensemble, with the timestep of 2 fs and the HOOVER thermostat and barostat set to 310.15 K and 1 bar, respectively. Van der Waals interactions were cut off between 10 and 12 Å using the force switch method (VFSWIt). The electrostatic potential used the force shifting method (FSHIft) with a cutoff of 12 Å. The particle mesh Ewald summation [49] was applied to address long-range electrostatic interactions. Bonds involving hydrogen atoms were constrained using the SHAKE algorithm.

Root-mean-square deviations (RMSD) were calculated with the MDAnalysis Python library [50, 51], and direct, as well as water mediated H-bonds were analyzed implementing the recently developed Bridge2 software [52, 53]. Each simulated system was also carefully visually inspected in order to confirm the accuracy of the predictions.

### Hydrogen-bond network analysis via Bridge2

All graph calculations were performed using Bridge/Bridge2, a graph-based algorithm with a user-friendly graphical interface that efficiently computes both direct and water-mediated H-bond interaction networks [52, 53]. H-bonds were identified using geometric criteria, which included either the donor-acceptor distance (from PDB structures) or, if hydrogen atom coordinates were available from MD simulations, a combination of distance and H-bond angle criteria. The H-bond angles are computed using an optimized implementation of the Einstein summation convention, applied to the position vectors of the donor, acceptor, and hydrogen atoms.

In H-bond graphs provided by Bridge/Bridge2, nodes represent protein groups involved in H-bonds, while edges denote either direct or water-mediated H-bonds. Bridge/Bridge2 is particularly efficient in calculating water-mediated bridges between protein groups. To identify potential H-bond donors and acceptors, the program uses a k-d tree approach, which scales as $n \times \log (n)$, where $n$ is the number of spatial data points, providing substantial efficiency compared to the naive method of calculating all pairwise distances, which scales as $n^2$.

We applied a distance criterion of $\le$ 3.5 Å between donor and acceptor atoms. For H-bonds identified from atomic-level MD simulations containing hydrogen atom coordinates, we also applied an additional H-bond angle cutoff of $\le$ 60°. Water-mediated bridges involving up to three water molecules were included in the H-bond graph analysis.

### Water density clustering using MADE (ProBiS H2O)

Water density clustering was performed using MADE software (ProBiS H2O). The MADE (Macromolecular Density and Structure Analysis) software basically supersedes previous ProBiS H2O (MD) approach. Implemented as a user-friendly PyMOL plugin, is a tool for identifying water/heteroatom conserved locations in proteins using experimental structural data, AlphaFold models or MD trajectories. The approach first performs structure alignment superimposed onto a query, where suitable protein chains are identified based on used alignment and superposition algorithm (e.g., PyMOL’s align and super, TM-align, DeepAlign, ProBiS, GANGSTA+). Then, 3D clustering follows using 3D-DBSCAN to locate dense regions where specific species (e.g., metal ions, water molecules, etc.) occur across the examined structures or trajectories. High conservation clusters signify biologically relevant sites. The last step is the prediction/identification of studied species conserved positions across MD trajectories or structural clusters with visualization in PyMOL. The approach is robust and can successfully generalize beyond waters and ions to other molecular species and can be used for water network analysis, dynamic binding events, and protein binding site elaboration.

For further structure validation, we examined the epicatechin-3-gallate bound to glutamate dehydrogenase protein conformation with the highest occupancy (main trajectory 0-600 ns; also found in a replica from 226 ns to the end). We collected 10 ns equidistant MD snapshots (took care to designate the chain on the protein and ligands, e.g., chain P, and set the HETEROATOM flag for corresponding atoms in the snapshot PDBs) and performed alignment using PyMOL’s align sequence-aware method on 61 snapshots alltogether. Then, all TIP3P waters were collected and subjected to 3D-DBSCAN using $\varepsilon = 0.9$ parameter and exploring all possible clusters. TIP3P water clusters with 16 or more molecules and conservation above 0.26 were visualized for the reader [22, 54].

## Results and discussion

### Diversity and characteristics of polyphenol-protein complexes in the extracted database

**Fig. 3:**
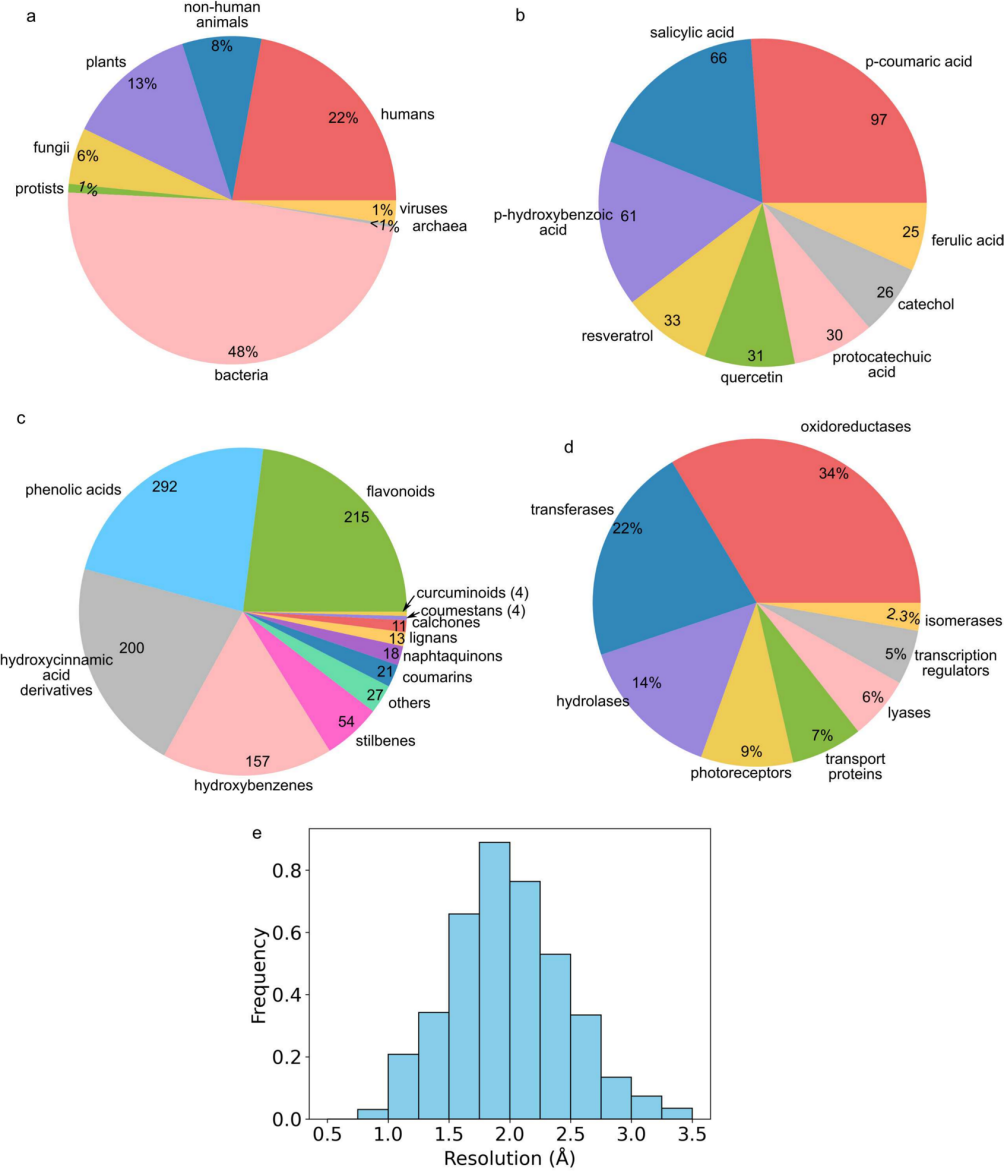
The main properties of the constructed protein-polyphenol database. **a** Classification of polyphenol-protein complexes into biological kingdoms. **b** The most represented polyphenols within the database. **c** Number of polyphenols in each class. **d** Classification of proteins containing polyphenolic ligands. **e** Distribution of polyphenol-protein complexes based on the resolution of the solved crystal structures. In all cases, alternative conformations are not counted separately

The extracted database encompasses proteins from all six biological kingdoms: animals, plants, fungi, protista, bacteria, and archaea, and also includes viral proteins (Fig. 3a). Bacterial proteins constitute the largest portion, representing 48% of the database, followed by human proteins at 22%. Non-human animals constitute approximately 8% to the database. The plant kingdom is represented by 13%, fungi by 6%, viruses by 2%, protists by 1%, and archaea by less than 1%.

The predominance of human and bacterial proteins highlights that the majority of research efforts related to polyphenols have been concentrated on their benefits in human health, as receptor/enzyme modulators or as antibacterial agents. The plant kingdom category contains a variety of enzymes involved in the synthesis of polyphenols, exemplified by the structure of chalcone synthases complexed with naringenin (PDB ID 7VF0). This enzyme catalyzes the condensation of one molecule of p-coumaroyl-CoA with three molecules of malonyl-CoA, forming naringenin chalcone, the precursor of all flavonoids [55].

The most frequently represented ligand in the database is p-coumaric acid, an isomer of hydroxycinnamic acid (Fig. 3b). This is followed by simpler structures such as salicylic acid and 4-hydroxybenzoic acid. More complex compounds like stilbene resveratrol and flavonoid quercetin are also prevalent. Hydroxycinnamic acid derivative ferulic acid follows, showcasing a diverse representation of polyphenols, ranging from low molecular mass structures to larger, more complex molecules.

The classification of ligands reveals that the majority belongs to the phenolic acids class, followed by flavonoids and hydroxycinnamic acid derivatives, hydroxybenzenes, stilbenes, and coumarins (Fig. 3c). Phenolic acids mostly include simple structures with a carboxylic group directly bound to a phenol ring, encompassing salicylic acid and gallate. Flavonoids cover various subclassifications such as flavanols, flavons, anthocyanidins, and isoflavonoids. Hydroxycinnamic acid derivatives feature typical structures like ferulic and caffeic acid, their esters (e.g., rosmarinic acid), and reduced derivatives like coniferaldehyde. Hydroxybenzenes describe simple structures, including all benzenediols and benzenetriols, along with typical essential oil constituents like eugenol and thymol. Other classes, such as stilbenes and lignans, maintain stricter definitions. 27 complexes containing polyphenols are classified under the “others” category due to the absence of an appropriate classification, with ellagic and mandelic acids forming notable examples.

The database is predominantly composed of enzymes, with oxidoreductases, transferases, and hydrolases forming the most represented classes (Fig. 3d). A relatively high number of structures also consists of photoreceptors, more specifically photoactive yellow protein, which contains p-coumaric acid as a chromophore [56].

Classifying crystal structures with a resolution of 2.0 Å or better as high resolution, approximately two-thirds of them meet this criterion (Fig. 3e). The importance of high resolution becomes evident when distinguishing ordered water molecules involved in ligand binding from free water molecules not engaged in binding interactions. High-resolution structures can facilitate the precise identification of individual ordered water molecules [57, 58]. In contrast, low-resolution structures may lack the detailed information necessary to discern fine hydrogen bonds and interactions between water molecules and surrounding atoms. The influence of resolution on the number of identified waters is well-established [20, 59].

On average, we found that structures with a resolution lower or equal to 2.0 Å exhibit approximately 1.9 water molecules in proximity to polyphenolic ligands, while strctures with resolution larger than 2.5 Å have only 0.6 water molecule on average around the ligand. The average number of detected bridging waters across all structures is 1.7. This underscores the significance of resolution in elucidating the water-mediated interactions crucial for understanding polyphenol-protein complexes, which we further explore in this work.

### Atom type preferences of polyphenols in binding to proteins and metals

We analyzed 272 protein-polyphenol atom pairs within a cutoff distance of 7.5 Å, utilizing SYBYL mol2 types (Supporting Information Table S2). Among these pairs, 94 occurred more than 1000 times (Supporting Information Table S3). The most prevalent pairs, constituting 43% of all cases, involved aromatic ligand carbons (C.ar) and various protein atoms, including sp3-hybridized side chain carbons (C.3$_{\hbox {sc}}$), aromatic side chain carbons (C.2$_{\hbox {sc}}$), and backbone atoms (C.3$_{\hbox {bk}}$, N.am$_{\hbox {bk}}$, O.2$_{\hbox {bk}}$, and C.2$_{\hbox {bk}}$). However, it is crucial to note that the abundance of these atom types is at the source of the high occurrence of these pairs, potentially reflecting general atom type prevalence rather than specific interactions.

**Fig. 4:**
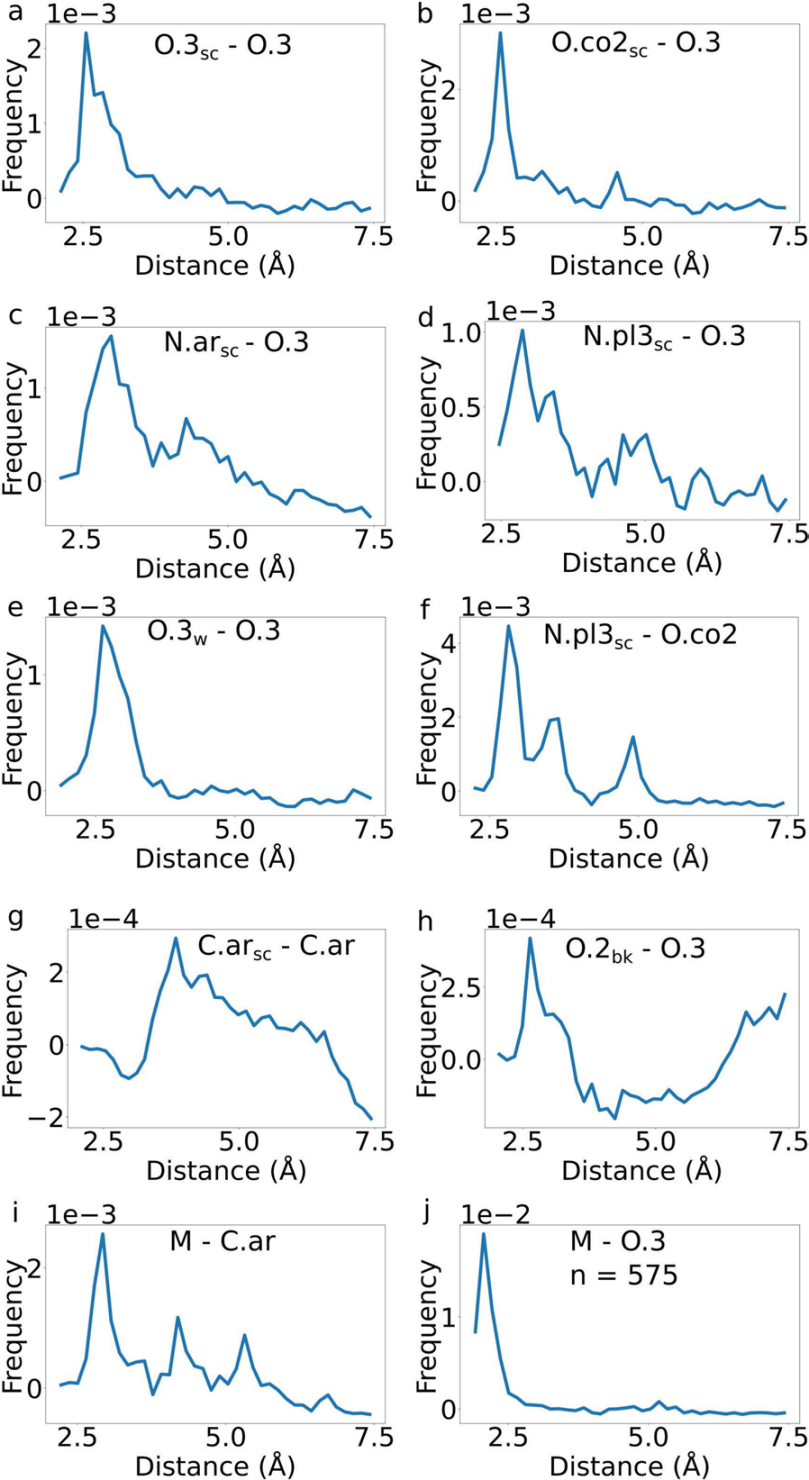
Radial pair distribution functions (RDFs) illustrating the spatial relationships between selected pairs of protein and polyphenolic atoms. The RDFs depict the distance distributions between: **a** O.3$_{\hbox {sc}}$ and O.3, **b** O.co2$_{\hbox {sc}}$ and O.3, **c** N.ar$_{\hbox {sc}}$ and O3, **d** N.pl3$_{\hbox {sc}}$ and O.3, **e** O.3$_{\hbox {w}}$ and O3, **f** N.pl3$_{\hbox {sc}}$ and O.co2, **g** C.ar$_{\hbox {sc}}$ and Car, **h** O.2$_{\hbox {bk}}$ and O.3, (**i**) metal ions (M) and C.ar, and (**j**) metal ions (M) and O.3 atom types. In all cases, the left-hand atom-type corresponds to a protein atom, while the right-hand atom corresponds to a polyphenolic atom. Each pair is present more than 1000 times, except for the M - O.3 pair, which is present 575 times. RDFs of other atom pairs that are present more than 1000 times are displayed in Fig. S1

The sp^3^-hybridized hydroxy groups (O.3) of polyphenols play a crucial role in their binding to proteins (Fig. 1). Strong H-bond interactions were observed between polyphenolic O.3 and side chain oxygens (O.3$_{\hbox {sc}}$) of Ser, Thr, and Tyr residues, denoted by a distinct peak in the radial distribution function (RDF) at approximately 2.8 Å (Fig. 4a) [60]. Additionally, H-bond and polar interactions were identified between polyphenolic O.3 and side chain carboxylate oxygens (O.co2) of Asp and Glu residues, as evidenced by the two peaks in the RDF at around 2.8 Å and 4.5 Å, respectively (Fig. 4b). Somewhat similar interaction motifs were detected between polyphenolic O.3 and side chain nitrogens (N.ar$_{\hbox {sc}}$ and N.3$_{\hbox {sc}}$) of Asn, Gln, and Arg residues (Fig. 4c and d). Moreover, polyphenolic catechols formed water bridges with water oxygens at around 2.8 Å, agreeing with the expectations of H-bond interactions [61] (Fig. 4e). Consistent interaction profiles were obtained when reversing the atom types between protein atoms and ligand atoms, affirming the reciprocity of these interactions (Fig. S1).

A distinctive three-peak profile is evident in instances where the side chain of arginine (N.pl^3^) interacts with the carboxylic acid group (O.co2) of polyphenolic compounds, exemplified by hydroxycinnamic and rosmarinic acids (Fig. 4f). The peaks, situated at around 2.7, 3.5, and 4.8 Å, likely signify different types of interactions: a peak at 2.7 Å could correspond to strong hydrogen bonds, the peak at 3.5 Å to weak hydrogen bonds, and the peak at 4.8 Å to salt bridges, demonstrating the multifaceted nature of interactions involved in polyphenol-protein binding [62].

The aromatic rings of polyphenols exhibit interactions with the aromatic side chains of His, Phe, Trp, and Tyr residues (Fig. 1), involving $\uppi$-stacking, $\uppi$-cation, and hydrophobic interactions (Fig. 4g). The distance distribution between the aromatic carbons of the polyphenols (C.ar) and the side chains (C.ar$_{\hbox {sc}}$) displayed a broad peak ranging from around 3.0 to 6.5 Å, indicating a spectrum of possible distances for $\uppi$-stacking interactions. Weaker and less specific interactions were also observed between aromatic carbons and sp^3^ or sp^2^ carbons, which can correspond to hydrophobic interactions (Fig. S1), contributing to the overall diversity and adaptability of polyphenol-protein interactions.

Certain protein backbone atoms, specifically the amide oxygen (O.2$_{\hbox {bk}}$) or nitrogen (N.am$_{\hbox {bk}}$), displayed a notable preference for binding to specific polyphenolic atoms, such as O.3 or carboxylic oxygens (O.co2), at approximately 2.7 Å. The observed proximity of these atoms suggests the formation of strong H-bonds between them (Fig. 4h, Supporting Information Fig. S1).

The most frequent interaction between metals and polyphenols primarily involved the aromatic carbons (C.ar) of the ligands, although this was largely influenced by the abundance of these ligand atoms. The RDF between metals and C.ar displayed three peaks: a prominent one at around 2.7 Å and two smaller ones at around 4.5 and 5.5 Å, respectively. These peaks suggest the attraction of metal ions to the $\uppi$-systems of the benzene rings and the shift of $\uppi$-carbons towards metal ions, particularly due to the binding of catecholic OH groups to metals (Fig. 4i). A more representative interaction between metals and polyphenols focused on the hydroxyl oxygens (O.3) of the ligands, occurring in 575 cases. The distance distribution between metals and O.3 exhibited a sharp peak at around 2.4 Å, followed by a rapid drop to zero (Fig. 4j), indicating a strong preference for metal-O.3 coordination. This preference highlights the significance of hydroxyl oxygens in mediating metal-ligand interactions within polyphenol-protein complexes.

All pair-pair RDF profiles that occur more than 1000 times are displayed in Supporting Information Fig. S1.

#### Interaction analysis across polyphenol classes

**Fig. 5:**
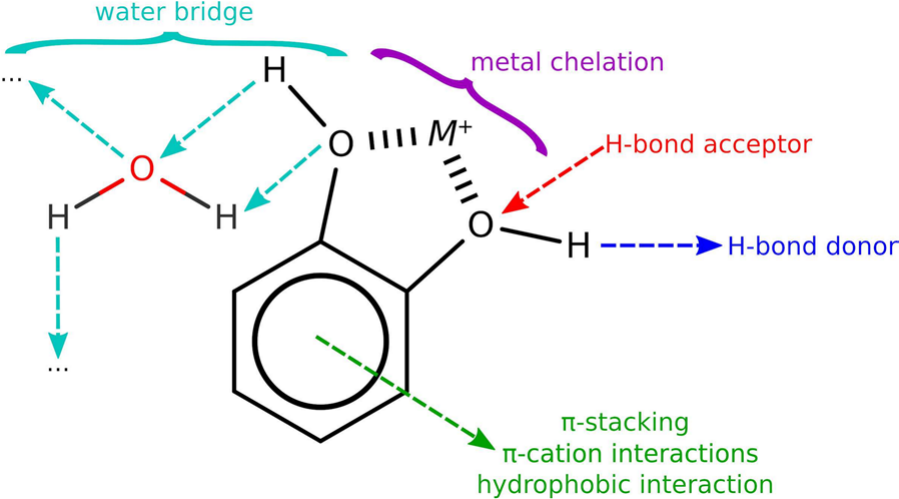
Representative noncovalent interactions commonly formed by standard polyphenolic compounds

The non-covalent interactions between polyphenols and proteins primarily involve hydrogen bonds, hydrophobic interactions, metal coordination, π-stacking, π-cation interactions, and salt bridges (Fig. 5, Tables S4-S15). Hydrogen bonds are typically formed between the amino, amide, and hydroxyl groups of amino acid residues and the hydroxyl groups of polyphenols. The phenolic hydroxyl group acts as both a hydrogen bond donor (via the H-atom) and acceptor (via the O-atom). Keto moieties, found in certain flavonoids (e.g., flavonones, flavanones), chalcones, coumarins, coumestans, curcuminoids, and naphthoquinones, also contribute as H-bond acceptors. Naturally, glycosyl moieties in glycosylated polyphenols are common contributors of hydrogen bonds.

**Fig. 6:**
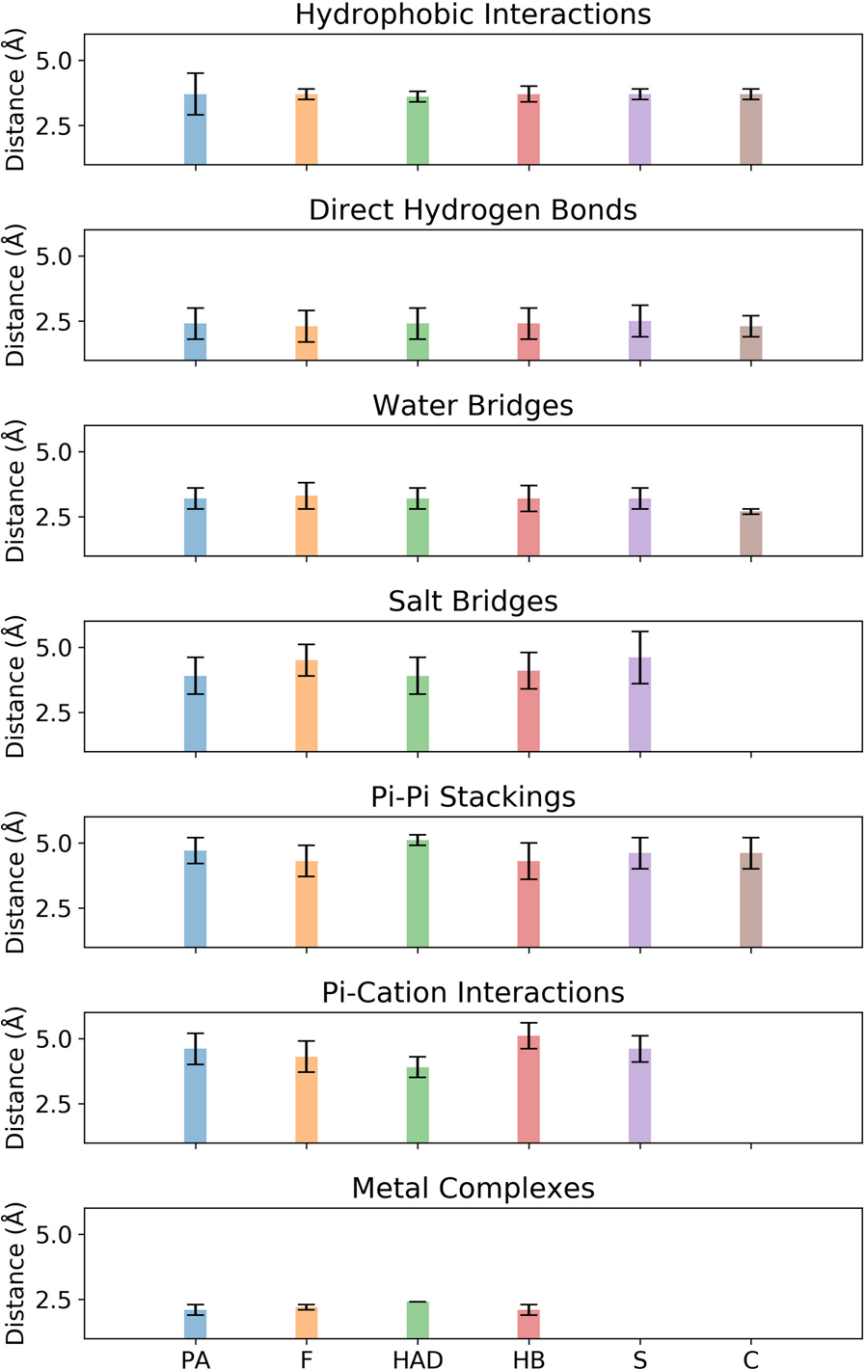
Overview of the average interaction distances and their standard deviations based on polyphenol classes. *PA* phenolic acids, *F* flavonoids, *HAD* hydroxycinnamic acid derivatives, *HB* hydroxybenzenes, *S* stilbenes, *C* coumarins

Direct H-bonds commonly form between these groups and amino acid side chains or backbone atoms, with average donor-acceptor distances ranging from approximately 3.1–3.4 Å and donor-hydrogen-acceptor angles around 135–145°. The average distances and standard deviations of direct H-bonds exhibit slight variations among different polyphenol classes, which suggests a consistent distribution and a pivotal role in complex stability (Fig. 6). Tyrosine and serine residues frequently participate in hydrogen bonding with polyphenols.

**Fig. 7:**
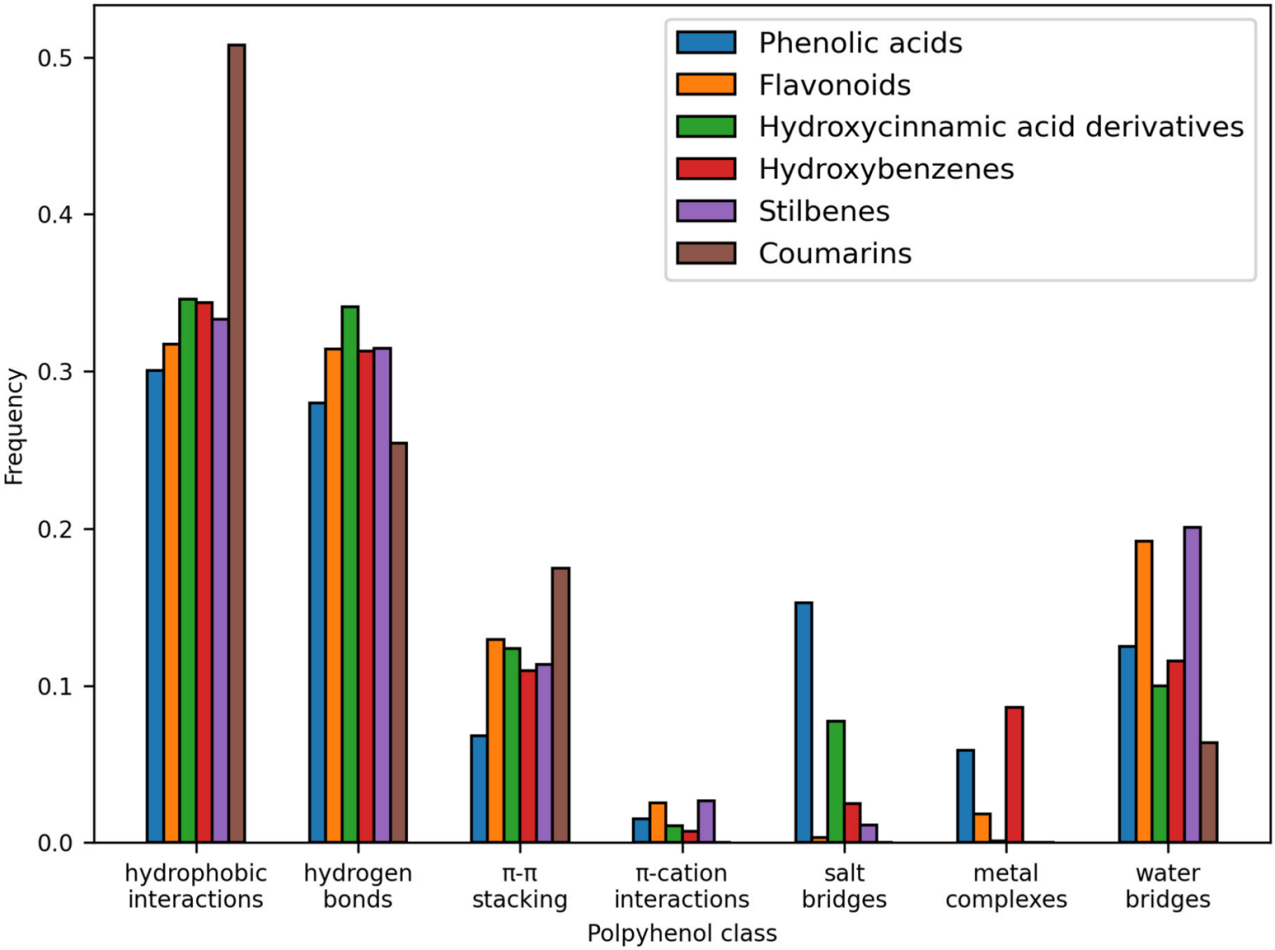
Relative frequency distributions of non-covalent interactions identified with PLIP, categorized by polyphenol classes with high representation in the PDB

Water-mediated H-bonds are prevalent across all classes, especially in compounds with a larger number of hydroxyl groups, like stilbenes and flavonoids (Fig. 7, Tables S5 and S8), enhancing their interaction networks within protein binding sites. The average distances of water bridges are generally similar across most polyphenol classes, although they are notably lower in coumarins (Fig. 6, Table S9), which is likely an artifact of low statistical sampling rather than specific structural properties.

Polyphenols, particularly those with gallol or catechol groups, are known for their strong metal-chelating properties, effectively coordinating metal ions such as iron and copper within metalloprotein binding sites [63]. Metal coordination generally involves the binding of polyphenol oxygen atoms (from hydroxyl or carboxyl groups) to metal ions. Phenolic acids and hydroxybenzenes frequently coordinate with metals, especially iron, forming complexes with average ligand-metal distances of around 2.1 Å (Fig. 6, Tables S4 and S7). Flavonoids also participate in metal coordination, binding ions such as Mn$^{2+}$, Ni$^{2+}$, Zn$^{2+}$, Mg$^{2+}$, or Fe$^{2+}$, with average distances of 2.2 Å and coordination numbers typically ranging from four to six, often adopting octahedral or trigonal bipyramidal geometries.

Stilbenes and coumarins generally do not engage in metal coordination within the observed PDB structures. The variability in metal coordination observed across different polyphenol classes may be attributed to differences in sampling size rather than inherent variations in metal-binding potential.

All classes of polyphenols form hydrophobic interactions with proteins, primarily through their aromatic rings and, in some cases, aliphatic linkers. Notably, cinnamic acid derivatives, stilbenes, lignans, chalcones, and curcuminoids possess significant aliphatic linkers connecting their aromatic rings, which enhance hydrophobic interactions with proteins (Tables S3, S8, S11, S13, S14). The average distance for these interactions is around 3.6$-$3.8 Å, once again indicating an overall stable interaction pattern across all classes (Fig. 6). Leucine and phenylalanine are the most commonly involved amino acid residues, interacting with polyphenols in a consistent manner across classes.

The aromatic systems of polyphenols are capable of forming geometrically varied π-stacking interactions with the side chains of phenylalanine, tyrosine, tryptophan, and histidine. These interactions typically involve either face-to-face (parallel) or edge-to-face (T-shaped) configurations. Among the more represented polyphenol classes-flavonoids, hydroxybenzenes, and stilbenes-both T-type and P-type π-stacking interactions are observed in roughly equal proportions (Table S5, S7, S8).

In contrast, phenolic acids and cinnamic acid derivatives generally adopt T-shaped stacking, with slightly larger distances of 4.7$-$5.1 Å and angles near 77–79° (Fig. 6, Tables S4 and S6). The increased propensity for T-stacking configurations observed for phenolic acids and hydroxycinnamic acid derivatives is likely due to the electron-withdrawing effects of their carboxyl groups, which alter the electronic properties of the aromatic ring. T-type stacking configurations generally have longer distances compared to P-type, contributing to the variation seen in average distances across these interactions (Fig. 6). The observed variations in average distances and standard deviations appear to be influenced by the inherent structural characteristics of each polyphenol class and reflect differences in their ability to participate in π-stacking.

$\uppi$-cation interactions involve the attraction between the electron-rich aromatic rings of polyphenols and positively charged side chains of lysine, arginine, and histidine. Flavonoids and stilbenes display relatively more $\uppi$-cation interactions compared to other classes, primarily with lysine and arginine residues, facilitated by their multiple aromatic rings and electron-rich systems (Fig. 7, Tables S5 and S8).

Salt bridges are formed only by polyphenols containing a carboxylate group capable of ionic interactions with positively charged residues lysine and arginine. Phenolic acids and cinnamic acid derivatives frequently form salt bridges due to their carboxylic acid moieties, with average heavy atom distances around 3.9 Å (Fig. 6, Tables S4 and S6). Hydroxybenzenes possessing a carboxyl group, such as hydroxyphenylacetic acids, also engage in salt bridge interactions. In contrast, flavonoids, stilbenes, and coumarins generally lack charged groups in their aglycone forms and thus rarely form salt bridges unless modified or conjugated with additional acidic groups.

For detailed discussions and notable examples of polyphenol-protein complexes with high representation in the PDB, please refer to the Supporting Information Section S1 and corresponding Fig. S2–S7. There we also provide the analysis of less represented classes (coumestans, lignans, naphthoquinones, curcuminoids, chalcones, and nonclassified polyphenols) in Section S2 and Fig. S8. These compounds engage in similar non-covalent interactions; however, caution is advised due to low data availability, when drawing definitive conclusions.

### Assessing the impact of crystallographic resolution and resolved waters on hydrogen bond networks

#### MD simulations of epicatechin-3-gallate binding to glutamate dehydrogenase solved at a low resolution: the role of water molecules and flexible binding modes

We employed MD simulations to explore the potential existence of water-mediated H-bonds in a low-resolution structure of the open conformation of bovine glutamate dehydrogenase (GDH, PDB ID 6DHL, resolution of 3.624 Å) bound to the flavonoid epicatechin-3-gallate (PDB ID: XEG) [64]. GDH represents an enzyme that catalyzes the oxidative deamination of glutamate to 2-oxoglutarate using NAD(P)$^{+}$ as a cofactor. It plays a crucial role in amino-acid metabolism and cellular energy production. Additionally, GDH is implicated in the regulation of insulin secretion by pancreatic beta-cells, and mutations in GDH can lead to hyperinsulinism/hyperammonemia syndrome, a rare genetic disorder affecting glucose and ammonia levels in the blood [64].

This structure was selected as it represents one of the lowest-resolution protein-polyphenol complex in the PDB. Our objective was to assess whether MD simulations could elucidate potential water-mediated H-bond network within the protein binding site, since the experimental crystal structure does not contain resolved water molecules.

In the initial crystal structure, epicatechin-3-gallate binds to the same allosteric site as the regulator ADP, with the interaction primarily driven by polar contacts (Fig. S9). Due to the absence of experimentally resolved water molecules and the likely dynamic nature of the binding mode, we used MD simulations to gain further insights into the interaction landscape of this complex, specifically focusing on the role of water molecules and the flexibility of the binding modes.

RMSD analysis of the main trajectory revealed two significant conformational transitions (Fig. S9a). Initially, the protein remained in a stable conformation for up to 0.5 µs, after which it transitioned to a new conformation around 0.75 µs and remained stable until the end of the simulation. Qualitatively, similar behavior was observed in the replica trajectory (Fig. S9b). By analysing the structural changes in the helical regions of XEG bound to GDH, we revealed two distinct conformations with notable transitions and moderate helical displacements while preserving the integrity of the binding site as elaborated in SI - Section S3, and Fig. S10.

To further characterize the distinct conformational states sampled during the simulations, we conducted hierarchical clustering based on RMSD matrices derived from 1,000 snapshots of both the main trajectory and the replica. In the main trajectory, the clustering analysis identified three conformational ensembles: the first spanning from 0 to 598 snapshots, the second from 599 to 745 snapshots, and the third from 746 to 1,000 snapshots (Fig. S9a). The replica trajectory showed two conformational states: the first spanning from 0 to 226 snapshots, and the second continuing until the end of the simulation (Fig. S9b). These observations indicate that the system samples distinct conformational ensembles, with the replica trajectory eventually settling into the same ensemble observed in the main trajectory.

**Fig. 8:**
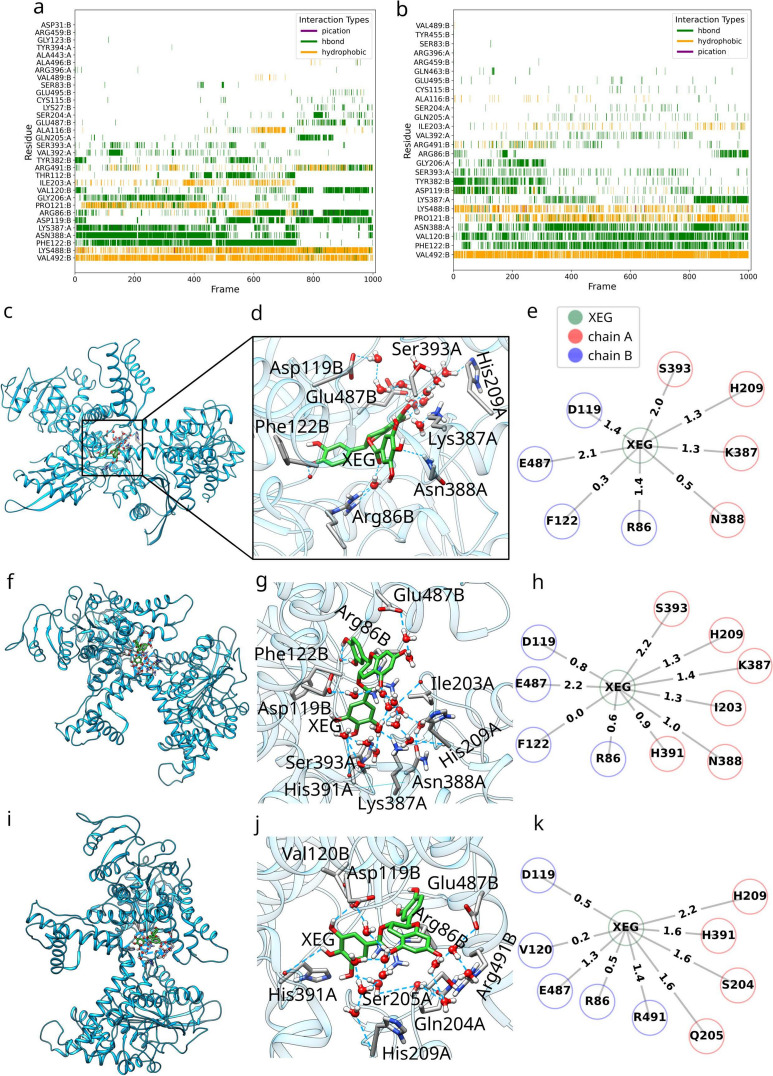
Interaction contact maps between glutamate dehydrogenase residues and XEG for the main **a** and replica **b** simulations, depicting the presence of hydrophobic (yellow), hydrogen bonds (green), and rare π-cation (purple) interactions. **c–k** Dynamic binding of epicatechin-3-gallate (XEG) to glutamate dehydrogenase (GDH). Analysis of the water-mediated binding for the first cluster (snapshots 0-598) simulation, showing the **c** entire medoid (409) snapshot, **d** zoomed-in binding site, and **e** the Bridge2 output of water-mediated H-bonding interactions. **f–h** panels corresponding to the second cluster (snapshots 599-745) and **i–k** panels corresponding to the third cluster (snapshots 746-1000). Blue cartoons in panels represent the backbone of GDH and sticks with grey carbons the amino-acid residues forming H-bonding interactions (cyan lines) with XEG. Sticks with green carbons represent the XEG ligand.The numbers on the edges represent the average number of water molecules bridging the H-bonding interaction

In addition to RMSD and clustering analyses, we performed a time-dependent analysis of protein-ligand interactions to investigate the evolution of key interactions during the simulation. Interaction fingerprints plotted over time demonstrated distinct interaction profiles that correlated with the observed conformational changes. In the main trajectory, an initial interaction pattern was observed up to approximately frame 500. This was followed by a transition that was completed around frame 750, correlating with the shift to a new conformational ensemble (Fig. 8a). In the replica trajectory, the initial stabilization phase involved consistent interactions with Asp119 and Tyr382, after which the system adopted a conformational ensemble similar to the beginning of the main trajectory (Fig. 8b), characterized by interactions involving Val120, Pro121, Phe122, Asn388, Lys488, and Val492.

Utilizing Bridge2 [52, 53], we examined the water-mediated hydrogen bond formation between XEG and the protein residues in the initial and subsequent time-frames based on the above-described clustering analysis throughout the MD simulation. Our analysis revealed the entry of numerous water molecules into the binding site, participating in dynamic H-bond networks involved in ligand binding, with observable changes over the course of the simulation.

Based on Bridge2 calculations, we observe in the initial crystal structure that the ligand XEG establishes direct H-bonding interactions with the side-chain atoms of Arg387A, Ser393A, Arg396A, Arg459B, Lys488B, and Arg491B (Fig. S9c-e). Additionally, two H-bonds are formed with backbone atoms of Cys115B and Val120B. During main MD simulations the three cluster-based water networks (snapshots at 0-598, 599-745, and 746-1,000 ps) are overall similar at high occupancy (more than 75%), with slight differences in the residues involved and the complexity of the water-mediated interactions. A similar water network was also identified throughout the entire simulation using the MADE approach as TIP3P waters persisted as conserved clusters at key bridging locations between XEG and also Asp119, Glu487, His85, Arg86, Lys387, Asn388, His209 and Ser393 (Section S4 and Fig. S11).

The water-mediated hydrogen bond networks across the simulations (Fig. 8c–k and Fig. S9f,g) reveal a dynamic and adaptable system, with a core set of residues-Asp119 and RArg86 from chain B and His209 from chain A-consistently participating in interactions. The initial cluster (frames 0-598, (Fig. 8c–e) contains the fewest residues, while the second cluster (599-745, (Fig. 8f–h) adds His391 and Ile203 from chain A, expanding the network. The third cluster (746-1000, (Fig. 8i–k) demonstrates significant rearrangements, losing interactions with F122 (chain B) and Ser393, Lys387, Asn388 (chain A) but gaining new ones with Val120, Arg491 (chain B) and His391, Ser204, Gln205 (chain A). Replica simulations show further evolution, with the first replica network (0-226, Fig. S9f) incorporating additional residues such as His85 and Tyr382 (chain B) and His195, His391, Ser204, Ser393 (chain A), resembling the second cluster of the main simulation. The second replica network (227-1000, Fig. S9g) adds Pro121 and Phe122 (chain B) and Gln205 (chain A), while losing His391 and Asn388, reflecting continued structural adaptation. These observations highlight a possibility of a highly dynamic water-mediated network, with residues and water molecules rearranging to accommodate structural and environmental demands.

Overall, this analysis underscores the crucial role of MD simulations in revealing binding water molecules. Our MD simulations shed light on the indispensable involvement of water molecules in the binding of polyphenols to proteins, a fact frequently disregarded in *in silico* studies, especially when dealing with low-resolution X-ray or cryo-EM structures. Additionally, they underscore that the crystal structure does not necessarily grasp the most representative pose of the protein-polyphenol complex, revealing the flexible nature of polyphenol binding observed in MD simulation.

Despite inherent limitations and uncertainties associated with MD simulations, such as the choice of force field, solvent model, simulation time, and sampling method, our consistent observation of dynamic and flexible polyphenol binding (Fig. 1), mediated by several water molecules, reinforces the robustness of these findings [16].

#### Influence of crystallographic resolution on water binding dynamics: comparative analysis of transthyretin-resveratrol complexes

We performed a comparative analysis of two TTR-resveratrol structures, one resolved at high resolution (PDB ID 7Q9O, R = 1.35 Å) and the other at lower resolution (PDB ID 1DVS, R = 2.00 Å), using 1 µs MD simulations to evaluate how crystallographic resolution impacts structural completeness. This analysis focused particularly on water-mediated hydrogen-bond networks and assessed the ability of MD simulations to compensate for any missing hydration details in lower-resolution structures.

**Fig. 9:**
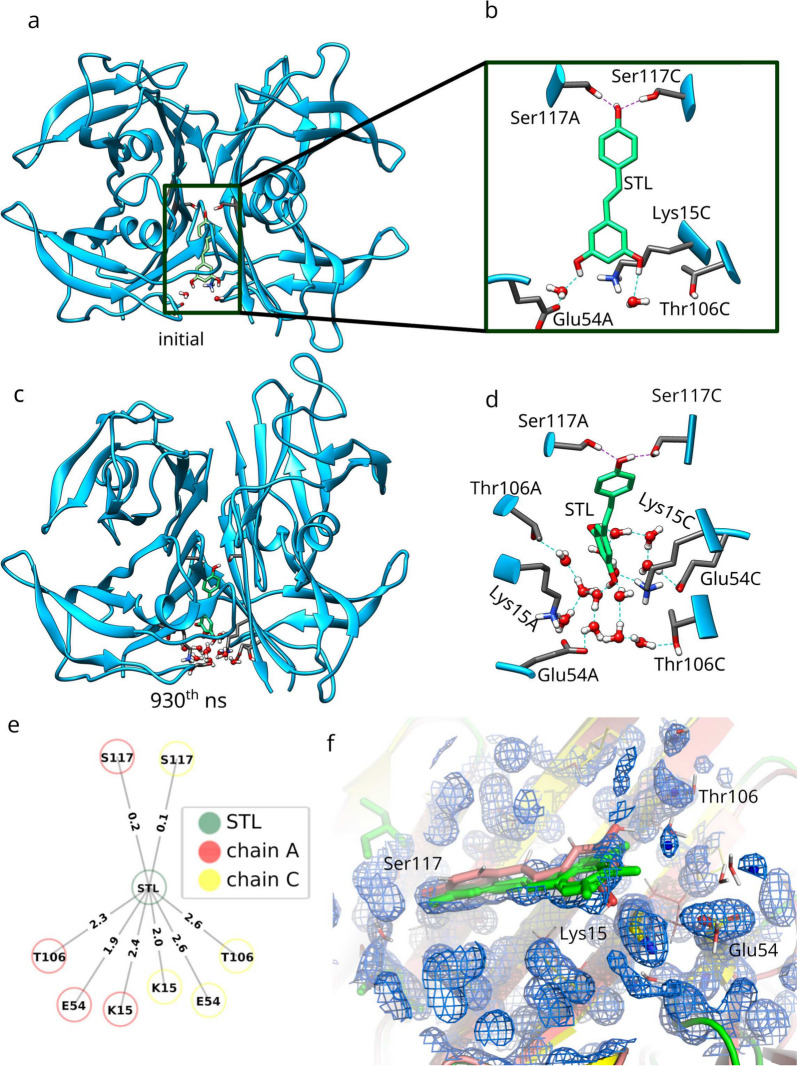
Water-mediated H-bonds were observed during the MD simulations of a TTR-resveratrol structure. **a**, **b** The lower-resolution crystal structure (1DVS) contains two water molecules within the binding site. One forms a water-mediated H-bond to Glu54A. **c**, **d** During MD simulations, an extensive water-mediated H-bond network was formed within the binding site, including residues from chains A and C. A frame from a 930$^{th}$ ns is chosen for visualization. **e** Bridge2 output of the resveratrol water-mediated H-bonding. Values on the edges represent the average number of bridging water molecules during the main MD simulation of the low resolution structure. Corresponding figures from remaining simulations are deposited in Supporting Information Fig. S11i-k. Blue cartoons represent the backbone of H-bonding amino-acid residues, and sticks with grey carbons their side-chains. Resveratrol (STL) is presented by sticks of green carbons, and waters by balls-and-sticks representation (red oxygens, white hydrogens). Direct H-bonds are shown with purple dotted lines, and water mediated ones with cyan dotted lines. **f** 7Q9O in yellow-colored cartoon model with green stick model ligand is overlaid with 2fo-fc electron density map in light-blue mesh. Crystal-modelled waters that were fitted to the electron density are emphasized by dark-blue spheres. Our MD snapshot is superposed (rose-colored cartoon model with light-pink stick model ligand) with MD TIP3 waters of the inspected snapshot in element-colored stick model (red oxygen and white hydrogens). It can clearly be observed that all modelled crystal water locations are also occupied by MD TIP3 waters

In the high-resolution structure (PDB ID 7Q9O, R = 1.35 Å), six water oxygen atoms are identified within three hydration layers around the resveratrol ligand, whereas in the lower-resolution structure (PDB ID 1DVS, R = 2.00 Å), only two such water oxygens are present (Fig. 9a, b). In the lower-resolution structure, one of the water molecules bridges further toward the side chain atoms of Glu54A.

Despite relatively high RMSD values in certain regions during the MD simulations, likely due to the inherent flexibility of the loop regions and peripheral domains (Fig. S12a-d), the core structure and ligand-binding interactions remained stable throughout the simulations (Fig. 9c–e). Time-dependent analyses of H-bond networks and key interaction patterns further confirmed this stability (Fig. S12e-k). Interaction contact maps for both high- and low-resolution starting structures across main and replica simulations revealed consistent and conserved interaction profiles over time.

This highlights the robustness of the TTR-resveratrol complex in maintaining critical ligand interactions, even amidst structural flexibility or when derived from lower-quality experimental data. Specifically, in both high- and low-resolution starting structures, the two direct hydrogen bonds with Ser117 and hydrophobic interactions with residues such as Leu108 and Ala110 were preserved, while additional water molecules entered the binding site during the simulations (Fig. 9c–e, S11e-k). Using a hydrogen-bond cutoff occupancy of $\ge$50%, the same symmetrical water-mediated network, involving Ser117, Thr106, Glu54, and Lys15, was consistently observed across all main and replica simulations, with minimal variation in the average number of bridging water molecules (Fig. 9e and S12i-k).

It is important to note that our pose selection positions the catechol moiety of resveratrol facing outward from the protein, a deliberate choice based on the existence of multiple poses documented for this complex (Fig. 9a, b) [65].

Moreover, a back-mapping approach was employed to analyze the observed water molecules in the high-resolution electron density map of the complex (PDB ID: 7Q9O). By superposing MD snapshots with the electron density map, it was possible to validate the placement of discussed water molecules in relation to the crystal structure. The electron density map, carved within 5 Å of the resveratrol ligand, revealed that the modelled crystal water locations were consistently occupied by waters from the MD simulation, underscoring the relevance of these molecules in the binding and stabilization of the complex. These findings elaborate on the functional importance of water molecules in mediating interactions between transthyretin and resveratrol, adding to a cohesive picture of their structural and dynamic contributions to complex stability.

In conclusion, our results demonstrate that starting from a lower-resolution structure, MD simulations are capable of reproducing the same water-mediated bonding patterns observed with high-resolution structures. This highlights the ability of MD simulations to provide reliable and reproducible insights into protein-ligand dynamics and molecular interactions, even when initial crystallographic data are of lower quality. It reinforces the value of MD simulations as a robust tool for investigating biomolecular systems under varying experimental conditions.

### MD simulation of the binding of quercetin and isoquercetin to sirtuin 6: the effect of glycosylation on dynamic polyphenol binding

Glycosylation plays a pivotal role in modulating the binding of polyphenols to proteins, influencing their biological activities and bioavailability [66, 67]. It serves as a natural mechanism for plants to regulate the activity and availability of polyphenols in response to environmental changes or stress conditions. Given the rapid metabolism of polyphenols upon their absorption, understanding the impact of glycosylation on polyphenol binding becomes crucial. Moreover, glycosylation emerges as a valuable biotechnological tool for modifying the properties and functions of polyphenols [68].

In general, glycosylation tends to diminish the binding affinity of polyphenols to proteins through a reduction in hydrophobicity and an increase in steric hindrance [69, 70]. Nevertheless, glycosylation may, in certain cases, elevate the binding affinity of specific polyphenols to particular proteins. This enhancement can occur through increased solubility, improved stability, heightened selectivity, and the formation of specific interactions with amino-acid residues within the protein structure [68].

Sirtuin 6 (SIRT6) stands out as a critical NAD$^{+}$-dependent protein deacylase homodimeric enzyme, playing a crucial role in metabolic regulation and maintaining chromatin homeostasis. Its activation has been linked to the protection against a spectrum of metabolic and age-related diseases, while its inhibition is associated with anticarcinogenic effects. The activation of SIRT6 has been attributed to quercetin, a polyphenol that binds to the isoform-specific acyl-binding channel of the protein. The complex formed between SIRT6 and quercetin (PDB ID 6QCD) represents a rare instance where the structures of both the polyphenol itself and its direct glycosylated derivative, isoquercetin (PDB ID 6QCE), are available in the PDB [71]. Isoquercetin, a glycosylated derivative of quercetin, has demonstrated a heightened selectivity as an activator of SIRT6 due to the sugar moiety preventing its binding to alternative sites in remaining SIRTs. Notably, in the crystal structure, the aglyconic part of isoquercetin retains an identical binding pose as the parent molecule, while the glycoside is oriented towards the cofactor in the isoquercetin (HW2) molecule, forming an H-bond with the ribose moiety [71].

To explore the influence of glycosylation on polyphenol behavior and activity, MD simulations again emerge as a vital tool. By comparing the structural dynamics of aglycon and glycon structures through MD simulations, we sought to elucidate how glycosylation affects the binding of polyphenols. In our simulations, both dimers of SIRT6 were considered, with each dimere binding the corresponding flavonoid.

Specific RMSD values (Fig. S13a-d) for this simulated system appear large due to the inclusion of the entire dimer in the simulation, with structural contributions from 81 residues in β-sheets, 166 in α-helices, and 324 in loop regions. The N-terminal and C-terminal ends also span 17 and 28 residues, respectively, which impacts RMSD values. Moreover, for protein-only RMSD plots, all backbone-C atoms were used, while other RMSD plots included all atoms. For example, the average RMSD for a 100 ns backbone calculation (main aglycone trajectory) was 4.58 Å ± 0.61, whereas considering only helical structures reduced the RMSD to 3.94 Å ± 0.52. Overall, we therefore consider the SIRT6 systems stable, and confirm this with the below described time-dependent interaction analyses.

Performing a time-dependent analysis of protein-ligand interactions, the simulations of the aglycone (quercetin) system showed notable consistency, with key hydrophobic residues such as Phe82, Phe86, Val115, and Phe86, maintaining high occupancies and substantial frame overlap across the main and replica simulations (Fig. S13e,f). Hydrogen bonding interactions, particularly with Pro62 and Leu186, were also present in both simulations, although their occupancies and timing displayed some variability. In contrast, the glycone (isoquercetin) simulations displayed greater variability. Interactions with residues like Val115, Ile185 and Leu186, but additional interactions, such as those involving Glu74, emerged in the glycone system (Fig. S12g,h). H-bonding of e.g. the main chain of Phe64 showed more fluctuations, likely due to the introduction of heightened flexibility by the glycone moiety.

**Fig. 10:**
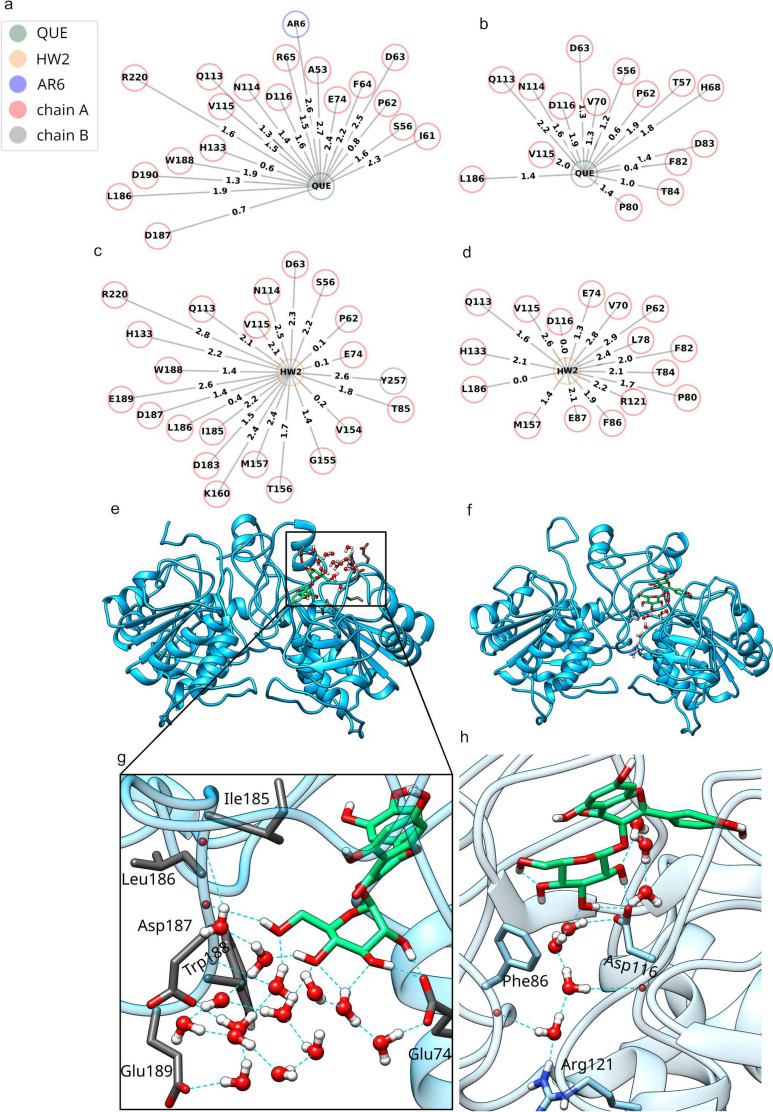
Water molecules mediate a large number of interactions between flavonoids and SIRT6 as observed during MD simulations. **a** Water-mediated H-bonds formed with quercetin (QUE) in the main MD simulation. **b** Water-mediated H-bonds formed with QUE in the replica MD simulation. **c** Water-mediated H-bonds formed with isoquercetin (HW2) in the main MD simulation. **d** Water-mediated H-bonds formed with HW2 in the replica MD simulation. **e**, **g** 3D view of the formation of a frequent (occupancy $\ge$ 50%) water-mediated H-bond network involving the sugar moiety of HW2 as observed in the main MD simulation. **f**, **h** 3D view of the formation of a frequent (occupancy $\ge$ 50%) water-mediated H-bond network involving the sugar moiety of HW2 as observed in the replica MD simulation

To complement the above analysis, Bridge2 was used to investigate water-mediated H-bonding networks in the last 400 ns of the simulation. This analysis revealed a high number of water-mediated interactions between the flavonoid ligands and the receptor, many of which showed frequent occupancy ($\ge$50%) (Fig. 10). While comparing the water-mediated H-bond networks formed in the main and replica simulations, we observed deviations from the strong correspondence observed in previous TTR-resveratrol simulations. This suggests a more pronounced transient nature of water-mediated H-bonds, resulting in rapid fluctuations of H-bonding patterns.

Interestingly, in simulations containing isoquercetin, we did not observe frequent interactions between the glycan moiety and the AR6 cofactor, as observed in the static crystal structure. However, our analysis revealed the formation of intricate water-mediated H-bond networks involving the polar sugar moiety, indicating the potential for glycosylation to introduce new interactions with proteins, primarily mediated by water molecules (Fig. 10e–h). This underscores the role of glycosylation in modulating protein-ligand interactions and emphasizes the significance of water-mediated H-bonds in fine tuning the protein interactions with polyphenols. For instance, in the main isoquercetin-SIRT6 MD simulation, we observed the formation of an extensive water-mediated H-bond network with the glycan moiety, encompassing amino-acid residues around Asp187 (Fig.  10c, e, g). In the corresponding replica MD simulation, a water network was formed between the glycan moiety and Phe86, Asp116, and Arg121 (Fig. 10d, f, h).

Here we, therefore, observe, that glycosylation can play a pivotal role in influencing the binding pose and orientation of the aglyconic polyphenol portion within the protein binding site, through altered formation of noncovalent interactions and especially through water-mediated networks by its polar glyconic part.

## Conclusion

Our study was initiated by analyzing polyphenol-protein interactions within the entire Protein Data Bank (PDB), which revealed a diverse array of polyphenolic structures encompassing smaller hydroxybenzenes and phenolic acids, to larger and distinct flavonoids, stilbenes, and lignans (Fig. 2). Across all these classes, polyphenols engage in a variety of noncovalent interactions with their protein targets - predominantly in hydrophobic interactions with aromatic rings or linkers, as well as in direct or water-mediated H-bonds with aromatic hydroxyl groups or, when present, carboxylic acid moieties (Fig. 7). Additionally, negatively charged carboxylic acids often form salt bridges. Frequent interactions also include $\uppi$-stacking and $\uppi$-cation interactions formed with aromatic rings. The catechol moiety of numerous polyphenols further facilitates metal complexation (Fig. 2). While similar overall interaction patterns are present among different polyphenolic classes, exceptions exist (Fig. 6); for instance, salt-bridge interactions are specific for charged moieties such as phenolic acids and hydroxycinnamic acid derivatives. Water-mediated hydrogen bonds are notably prevalent in larger polyphenols with abundant aromatic hydroxyl groups arranged in a planar and rigid structure (flavonoids, stilbenes). Another exception is the occurrence of $\uppi$-stacking interactions, which are less frequent in phenolic acids compared to remaining polyphenols, likely due to the electron-withdrawing effects of the carboxylic acid moiety (Fig. 6).

In the second part of our study, we explored various polyphenol-protein complexes using MD simulations to uncover the intricate dynamics of their interactions. Our exploration revealed dynamic binding patterns characterized by the initial influx of water molecules into the binding site, prominently observed in the XEG-GDH complex, as well as in the binding of QUE and HW2 to SIRT6. These findings underscore the limitations of static crystal structures in capturing the most representative poses of protein-polyphenol complexes. Notably, water-mediated interactions emerge as crucial in polyphenol-protein binding due to the presence of multiple polyphenoloic polar groups, rendering the overall interaction patterns highly dynamic. Water molecules actively participate in mediating transient H-bonds between the ligand and protein residues via established water networks. The latter possibly contributes to the remarkable variability in the binding pattern of polyphenols, as well as to their promiscuous binding. The observed flexibility therefore advocates water-mediated H-bond and conserved water molecule analysis in polyphenol-protein interaction study.

Conversely, the comparison between high and low-resolution crystal structures of TTR-resveratrol, coupled with extensive MD simulations, highlights the robustness of carefully curated MD simulations. These simulations reveal consistent dynamics, despite the difference in resolution of the initial structural data.

Our investigations into the influence of glycosylation on polyphenol binding hint to its role in modulating interactions with proteins. Glycosylation, functioning as a natural polyphenolic regulatory mechanism in plants, can impact the overall binding of the aglyconic part of the polyphenol by facilitating the formation of intricate water-interaction networks between the glyconic part of the polyphenol and the protein, either promoting or abolishing the activity.

In contrast to synthetic drugs, which frequently exhibit stable and specific binding modes [72], polyphenol binding seems to lack the typical stability seen in ligand-protein complexes representing synthetic actives. Instead, the dynamic nature of polyphenol interactions, primarily driven by the propensity to form water-mediated interactions, underscores the intricate interplay among the ligand, protein, and solvent environments. This emphasizes the necessity for comprehensive dynamical studies aimed at elucidating the molecular mechanisms underlying polyphenol-protein recognition.

In essence, our comprehensive analyses contribute to a deeper understanding of the nuanced interplay between polyphenols and proteins. This knowledge not only enhances our grasp of the molecular mechanisms, but also provides a foundation for future studies aimed at harnessing the therapeutic potential of polyphenols through informed drug design.

## Supplementary Information

Supplementary material 1. Section S1: Binding Patterns of Polyphenols with High Representation in the PDB. Section S2: Binding Patterns of Polyphenols with with Low Representation in the PDB. Section S3: Helical Regions Around the XEG Binding Site. Section S4: Density-Based Clustering Analysis of Waters of the GDH-XEG System using MADE. Table S1: Polyphenols identified from the Protein Data Bank. Table S2: Atom types used in this work. Table S3: Number of atom pairs derived from the protein-polyphenol complexes. Tables S4-S15: Overview of the main interaction properties for each polyphenolic class. Figure S1: Normalized radial distributions for all protein atom – polyphenol atom pairs exhibiting more than 1000 occurrences. Figures S2-S8: Examples of non-covalent interactions based on polyphenol classes. Figure S9: RMSDs, structural binding analysis and Bridge2 outputs of the glutamate dehydrogenase-epicatechin-3-gallate MD simulations. Figure S10: Glutamate dehydrogenase conformations overlap. Figure S11: Density-Based Clustering Analysis of Waters of the GDH-XEG System using MADE. Figuere S12: RMSDs, time-dependent interaction contact maps and Bridge2 outputs for the transthyretin-resveratrol MD simulations. Figure S13: RMSD and time-dependant interaction contact maps for the SIRT6 MD simulations. MADE report for the GDH-XEG system.

## Data Availability

No datasets were generated or analysed during the current study.
