# Supplementary material for: Unveiling polyphenol-protein interactions: a comprehensive computational analysis
Source: J Cheminform. 2025 Apr 10;17:50. doi: 10.1186/s13321-025-00997-3 (PMC11983793; doi:10.1186/s13321-025-00997-3)
Supplement: Supplementary file 1 — Supplementary material 1. Section S1: Binding Patterns of Polyphenols with High Representation in the PDB. Section S2: Binding Patterns of Polyphenols with with Low Representation in the PDB. Section S3: Helical Regions Around the XEG Binding Site. Section S4: Density-Based Clustering Analysis of Waters of the GDH-XEG System using MADE. Table S1: Polyphenols identified from the Protein Data Bank. Table S2: Atom types used in this work. Table S3: Number of atom pairs derived from the protein-polyphenol complexes. Tables S4-S15: Overview of the main interaction properties for each polyphenolic class. Figure S1: Normalized radial distributions for all protein atom – polyphenol atom pairs exhibiting more than 1000 occurrences. Figures S2-S8: Examples of non-covalent interactions based on polyphenol classes. Figure S9: RMSDs, structural binding analysis and Bridge2 outputs of the glutamate dehydrogenase-epicatechin-3-gallate MD simulations. Figure S10: Glutamate dehydrogenase conformations overlap. Figure S11: Density-Based Clustering Analysis of Waters of the GDH-XEG System using MADE. Figuere S12: RMSDs, time-dependent interaction contact maps and Bridge2 outputs for the transthyretin-resveratrol MD simulations. Figure S13: RMSD and time-dependant interaction contact maps for the SIRT6 MD simulations. MADE report for the GDH-XEG system. [file 13321_2025_997_MOESM1_ESM.docx]

**Supporting Information**

**for**

**Unveiling Polyphenol-Protein Interactions: A Comprehensive Computational Analysis**

Samo Lešnik^1,2^, Marko Jukič^1,3^, Urban Bren^1,2,3,*^

^1^Laboratory of Physical Chemistry and Chemical Thermodynamics, Faculty of Chemistry and Chemical Engineering, University of Maribor, Smetanova 17, SI-2000 Maribor, Slovenia

^2^IOS, Institute of Environmental Protection and Sensors, Beloruska 7, SI-2000 Maribor, Slovenia

^3^Faculty of Mathematics, Natural Sciences and Information Technologies, University of Primorska, Glagoljaška 8, SI-6000 Koper, Slovenia

# S1. Binding Patterns of Polyphenols with High Representation in the PDB

## Phenolic acids

Phenolic acids, a prominent class of polyphenols, are characterized by a phenolic ring directly linked to a carboxylic acid moiety. This class is the most abundant in our database and is widely distributed in plants. Known for their antioxidant and health-promoting properties, phenolic acids play a crucial role in various pharmacological effects. Gallic acid serves as a representative example of a hydroxybenzoic acid, a subclass of phenolic acids distinguished by three hydroxyl groups on the phenolic ring. Overall, phenolic acids exhibit diverse pharmacological effects, encompassing anti-inflammatory, antimicrobial, anticancer, neuroprotective, and cardioprotective activities. For example, gallic acid has demonstrated its capability to inhibit the growth of various cancer cells, as well as to provide protective effects against oxidative stress and neurodegeneration [1–4].

Phenolic acids can engage in hydrophobic interactions, a non-specific bonding between non-polar or weakly polar groups. In particular, the carbons of the aromatic ring of phenolic acids can establish extensive hydrophobic contacts with the protein receptor. Leucine represents a common amino-acid residue involved in these interactions, accounting for 21% of cases (Supporting Information Table S4). On average, the distance between the carbons of the aromatic ring and the interacting receptor is 3.7 Å.

Phenolic acids exhibit H-bonding interactions with proteins, representing a specific interaction where a hydrogen atom, covalently bound to an electronegative atom (donor), interacts with another electronegative atom (acceptor) at a specific distance and angle. The carboxylic and hydroxyl groups within phenolic acids are responsible for forming both direct and water-mediated H-bonds with protein atoms. Notably, 75% of direct H-bonds involve protein side chains, with nearly a quarter occurring with tyrosine (Supporting Information Table S4). On average, the distance between the donor and acceptor heavy atoms (DA) is 3.1 Å, while the predicted distance between the donor hydrogen and acceptor (HA) is 2.4 Å. The average donor-H-donor-acceptor (HDA) angle is 141°. H-bonds are formed with both the carboxylic and hydroxyl moieties of phenolic acids in approximately equal shares. About one-third of these interactions involve water-mediated H-bonds, often with arginine (Supporting Information Table S4). The average distances are 3.3 Å between the acceptor and water heavy atoms (AW), 3.2 Å between the donor and water (DW), with the average donor-donor hydrogen-water oxygen (DHW) angle at 135°. Moreover, the average angle formed around acceptor-water oxygen-donor hydrogen (AWH) is 96°.

Phenolic acids also engage in salt bridge interactions with proteins, a strong non-covalent interaction combining H-bonding and ionic bonding with an energy contribution of around -3 kcal/mol [5–8]. Salt bridges are formed between oppositely charged groups, specifically between the carboxylate group of phenolic acids and the positively charged side chains of lysine and arginine. The majority of salt bridges, approximately 81%, are formed with arginine, while the remaining interactions involve lysine (Supporting Information Table S4). The average length of the salt bridge interaction, measured between heavy atoms, is 3.9 Å.

Phenolic acids can participate in π-stacking interactions with proteins, an attractive force between aromatic rings dependent both on their relative orientation and distance. However, these interactions are less common for phenolic acids compared to other polyphenols. This lower frequency may be attributed to the electron-withdrawing effects of the carboxylate group, diminishing the electron density of the aromatic ring and potentially reducing electrostatic attraction between the rings. Additionally, the negative charge and steric effect of the large carboxylate group might hinder the optimal alignment of aromatic rings in stacking interactions. Phenylalanine is the primary amino-acid residue involved in these interactions (Supporting Information Table S4). The prevalent T-type stacking configuration is observed in 72% of cases, with an overall average distance between ring the centers at 4.7 Å (3.8 Å for P-stacks and 4.9 Å for T-stacks). The average offset of the phenolic ring center in both configurations is approximately 1.1 Å. In T-stacks, the average angle between ring planes is 79°, while for P-stacks, it is 7°.

In addition to the already described interactions, phenolic acids also demonstrate π-cation interactions with proteins. These interactions entail attractive forces between the aromatic rings of phenolic acids and positively charged groups of proteins. Although relatively infrequent, we identified 15 cases across 10 protein structures (Supporting Information Table S4). Lysine is the primary amino-acid residue involved in these interactions, while histidine and arginine also contribute. The average distance of these π-cation interactions is approximately 4.6 Å, with an offset of around 1.2 Å.

Metal coordination is a type of interaction where a metal ion binds to one or more polyphenolic atoms acting as donors. Given their structural characteristics, phenolic acids demonstrate a remarkable ability to form complexes with metal ions. This complexation can occur both with the aromatic hydroxy moieties and with the oxygen atoms of the carboxylic group. The prevalent geometry observed in these interactions is typically trigonal bipyramidal, followed by square pyramidal arrangements (Supporting Information Table S4). The average distance between the ligand atom and the metal center measures approximately 2.1 Å. While iron ions predominantly partake in these coordination events, there are instances where other metal ions also engage in the complexation process.

The *Pseudomonas putidate* PcaY_PP chemoreceptor (PDB ID 6S33) represents an exemplary illustration of a high-resolution structure (1.56 Å) engaged in the binding of the phenolic acid protocatechuate (3,4-dihydrobenzoic acid, PDB ID DHB) (Figure S2). This receptor, integral to the chemosensory system of *Pseudomonas putida*—a soil bacterium renowned for its ability to degrade diverse pollutants—offers a comprehensive example of the manifold noncovalent interactions facilitated by phenolic acids. Within the structure, protocatechuate binds at the homodimer interface, establishing a salt bridge interaction between its carboxylic moiety and the side-chain of Arg71A. Moreover, the carboxylic moiety forms direct H-bond interactions with Asn75A, while the catechol hydroxyl groups engage in H-bonding with Ser78A, Ser73B, and Gln142B. Furthermore, a water-mediated H-bond emerges between one of the catechol hydroxyl groups and Ser77B. The aromatic ring of protocatechuate forms also hydrophobic interactions with Ala74A and Ile70B.

## Flavonoids

Flavonoids, constituting a large and diverse class of plant secondary metabolites, exhibit various biological activities and health benefits. Characterized by a C6-C3-C6 skeleton, these compounds consist of two aromatic rings (A and B) connected by a three-carbon chain forming a third ring (C) (Figure 3) with different degrees of oxidation and substitution [9]. This structural configuration defines the distinct features and functional properties associated with flavonoids, contributing significantly to the chemical diversity found in plant-derived compounds.

Flavonoids exhibit diversity in the chemical structure of their rings and chain, including variations in the number and position of hydroxyl groups, the degree of oxidation, and the presence of double bonds. This structural heterogeneity allows for the classification of flavonoids into distinct subclasses, such as anthocyanidins, flavonols, flavanones, flavan-3-ols, flavanonols, flavones, and isoflavonoids [10].

For example, flavanones, prevalent in citrus fruits, exhibit antimicrobial and antiviral effects [11]. On the other hand, flavan-3-ols, prominent in tea catechins, contribute to cardioprotective and anticarcinogenic effects [12]. Isoflavonoids, primarily found in legumes, exhibit estrogenic and anticarcinogenic effects [13, 14].

Quercetin, kaempferol, apigenin, luteolin, genistein, and naringenin stand out as noteworthy flavonoids frequently identified as ligands in the PDB database (Supporting Information Table S1) [15–22]. These flavonoids have demonstrated interactions with diverse proteins engaged in signaling pathways, enzyme catalysis, gene regulation, and cellular structure. Given their versatile roles, flavonoids, including the listed compounds, have gained significant attention in medicinal chemistry and pharmacology.

The aromatic ring carbons of flavonoids can form significant hydrophobic interactions with the protein receptor, predominantly establishing contacts with leucine residues in about 21% of cases (Supporting Information Table S5). The average distance of these interactions is approximately 3.7 Å.

Additionally, flavonoids exhibit H-bonding with proteins. The hydroxyl groups on the benzopyrone core or the phenyl ring of flavonoids typically act as acceptors, with the chromene oxygen also serving as an acceptor. Moreover, methoxy groups present in certain flavonoids, such as isorhamnetin, are observed to form H-bond interactions with proteins. Flavonoids can establish both direct and water-mediated bonds with protein atoms. The majority of direct H-bonds involve protein side chains (75%), with a significant portion formed with serine residues (Supporting Information Table S5). Lysine is the most common positively charged residue involved, while aspartate represents the most common negatively charged residue. When H-bonds are formed with main chain atoms, valine is the most common interacting residue, followed by glycine. The average HA distance in side-chain interactions is 2.6 Å, with an average DA distance of 3.3 Å. The average HDA angle is 135°.

Flavonoids also frequently engage in water-mediated H-bonds with proteins, and no strong specific amino acid preferences are observed for these interactions (Supporting Information Table S5). The average AW and DW distances are both 3.3 Å. The average DHW angle is 135°, and the average AWH angle is 92°.

π-stacking interactions are more frequent for flavonoids than for phenolic acids, which could be explained by the higher electron density of the aromatic rings. π-stacking interactions are mostly formed with phenylalanine, but can also include tyrosine, histidine, or rarely tryptophan (Supporting Information Table S5). In most cases, face-to-face stacking is observed (58%), with an average distance between ring centers of 3.9 Å and an average angle between ring planes of 11°. T-type stacking is also observed (42%), with an average distance of 5.0 Å and an average angle of 79°. All three flavonoid ring moieties (A, B, and C) can form π-stacking interactions.

π-cation interactions are also relatively more prevalent among flavonoids compared to phenolic acids. These interactions predominantly occur with lysine and arginine side chains (Supporting Information Table S5). The phenyl ring, as well as both rings of the benzopyrone moiety in flavonoids represent the π-system engaged in these interactions. On average, π-cation interactions exhibit a distance of 4.3 Å, with an average offset of 1.3 Å between the center of charge and the ring center.

Flavonoids can also engage in interactions with metal ions, particularly through their catecholic moieties. These metal complexes are predominantly formed with Mn^2+^, but also involve Ni^2+^, Zn^2+^, Mg^2+^, or Fe^2+^ (Supporting Information Table S5). The average distance observed in metal coordination is 2.2 Å, and the coordination number typically ranges between four and six, with a prevailing tendency towards octahedral arrangements.

Most flavonoids lack a charged carboxylate group in their aglycone part, limiting their potential to engage in salt bridge interactions with proteins. However, in two instances (five alternative conformations, Supporting Information Table S5), this interaction is observed when the protein binds baicalein, a flavonoid conjugated with β-D-glucopyranosiduronic acid. This is found in human-5’-nucleotidase (PDB ID: 4H2B) and the toxic ricin A (PDB ID: 4Q2V), where the sugar-acid moieties form salt bridge interactions with the side chains of arginines.

Luteolin, through its interactions with transthyretin as observed in the exemplary high-resolution structure (PDB ID 4DEW), has the potential to stabilize the protein's structure, thereby impeding the formation of amyloids [23]. The high-resolution structure of transthyretin bound to luteolin (Figure S3) reveals a diverse array of noncovalent interactions. Hydrophobic interactions are notably formed with the carbons of the A and C rings. Additionally, direct H-bonds are established between the hydroxyl groups of ring B and residues Ser117B/D and Thr119D, while ring A forms H-bonding interactions with Lys15B. Moreover, water-mediated H-bonds are observed involving the B and C rings with amino-acid residues Leu17D, Leu110B and Lys15B. Finally, a π-cation interaction is identified between ring A and Lys15D.

Furthermore, in the following example, we present a high-resolution structure depicting a complex formed between the 8-C-glucoside luteolin derivative orientin and influenza A endonuclease (PDB ID 7NUG) (Figure S3b), a target for antiviral drugs [24]. Notably, only polar interactions are observed in this structure. Specifically, a water-mediated H-bond network is formed, involving the sugar moiety of the flavonoid orientin and side chains of Tyr130 and Arg124, as well as the main chain of Arg196. Additionally, influenza A endonucleases typically chelate either Mn^2+^ or Mg^2+^ ions within the catalytic site. In the depicted structure, the catecholic moiety of ring B of orientin is observed to bind two metal ions (Mn^2+^ or Mg^2+^).

## Cinnamic acid and Derivatives

Cinnamic acid and its derivatives represent important phenolic compounds that are widely distributed in plants and exhibit various biological activities [25]. These compounds are interesting from the perspective of medicinal chemistry and pharmacology because they can modulate different molecular targets and pathways involved in inflammation, oxidative stress, cancer, diabetes, viral infection, and neurodegeneration [26, 27]. Cinnamic acid and derivatives are characterized by a phenylpropenoic acid structure, which consists of a phenyl ring and a propenoic acid group. They can be found in various plants, including cinnamon, coffee, berries, and grapes.

Caffeic acid is a hydroxycinnamic acid that has two hydroxyl groups on the phenyl ring. It exhibits antioxidant, anti-inflammatory, anticancer, antiviral, and neuroprotective properties. Additionally, it may modulate glucose metabolism [28].

Chlorogenic acid, an ester of caffeic acid and quinic acid, stands as one of the most abundant polyphenols in coffee, showcasing antioxidant, anti-inflammatory, antidiabetic, and antimicrobial activities [29]. Its potential extends to mitigating photoaging and photodamage of the skin by inhibiting matrix metalloproteinases and alleviating oxidative stress [30]. In a similar ester category, rosmarinic acid, formed from caffeic acid and 3,4-dihydroxyphenyllactic acid, is prevalent in plants of the mint family such as rosemary, basil, oregano, and sage [31]. Renowned for its antioxidant, anti-inflammatory, antiviral, antibacterial, and anti-allergic properties, rosmarinic acid is believed to modulate immune system function and provide protection against neurodegeneration [32, 33].

Cinnamic acid and its derivatives can engage in salt bridge interactions with proteins. This interaction predominantly involves the carboxylic acid group of cinnamic acid derivatives and the positively charged side chains of lysine or arginine (Supporting Information Table S6). In this context, the carboxylic acid is tethered to an alkyl chain, resulting in a slightly elevated pKa (approximately 4.4) compared to aromatic phenolic acids. The average distance between the heavy atoms in these salt bridge interactions measures around 3.9 Å.

The ring system and the C3 skeleton, which connects the ring and the carboxylic moiety of cinnamic acid derivatives, engage in hydrophobic interactions with the protein. These interactions exhibit an average distance of 3.6 Å, and phenylalanine emerges as the most frequently interacting amino-acid residue (Supporting Information Table S6).

Cinnamic acid and its derivatives can form H-bonding interactions involving the hydroxyl groups of the phenyl ring and the acidic carboxyl group. Additionally, methoxy groups present in certain cinnamic acid derivatives, such as ferulic acid, have been observed to form H-bonds with the protein. These derivatives can engage in both direct and water-mediated hydrogen bonds with protein atoms. Most direct hydrogen bonds (62%) occur with protein side chains, with tyrosine being the most frequently interacting residue (Supporting Information Table S6). Lysine and aspartate emerge as the most common positively and negatively charged interacting residues, respectively. In cases where hydrogen bonds involve protein main chain atoms, cysteine represents the most common interacting residue. The average hydrogen bond HA and DA distances are 2.6 and 3.1 Å, respectively, while the average HDA angle is 142°.

Cinnamic acid derivatives also frequently participate in water-mediated H-bonds, with no strong specific amino-acid preferences observed (Supporting Information Table S6). The average AW and DW distances are 3.2 and 3.4 Å, respectively. The average DHW angle is 135°, and the average AWH angle is 96°.

π-stacking interactions with cinnamic acid derivatives occur with a similar frequency like in other polyphenols. These interactions predominantly adopt the T-stacking configuration, primarily involving phenylalanine residues (Supporting Information Table S6). The average distance between the ring centers is 5.1 Å, and the average angle between the ring planes is 77°.

Cinnamic acid derivatives exhibit a limited number of π-cation interactions, with only nine instances observed (Supporting Information Table S6). These interactions are equally distributed among histidine, lysine, and arginine residues. Moreover, interactions with metal ions are nearly nonexistent.

As an example, the human protein kinase CK2 alpha, recognized for its antiapoptotic and cancer-sustaining properties (PDB ID 6HOP), provides a high-resolution structure (1.55 Å) illustrating the binding of a cinnamic acid derivative, ferulic acid (PDB ID FER) [34]. In this interaction, the acidic moiety of the ligand establishes a salt bridge with a lysine residue (Lys68) (Figure S4). Additionally, the acidic moiety forms a direct H-bond with the backbone of Trp176 and a water-mediated H-bond with the backbone of Asp175. Moreover, the catechol group engages in two hydrogen bond interactions with the backbone of Val116. The nonpolar portion of the ligand (aromatic C-atoms and the linker) participates in hydrophobic interactions with several lipophilic residues.

## Hydroxybenzenes

Hydroxybenzenes constitute a class of aromatic compounds characterized by the presence of one or more hydroxyl groups attached to a benzene ring. This category encompasses simple structures like benzenediols (catechol, resorcinol, hydroquinone) and benzenetriols (pyrogallol, hydroxyquinol, phloroglucinol), which contain two or three hydroxyl groups, respectively. Additionally, it includes more intricate molecules like eugenol and thymol, prominent components of essential oils extracted from various plants. The biological and chemical properties of hydroxybenzenes vary depending on factors such as the number, position, and substitution pattern of the hydroxyl groups. For instance, eugenol exhibits antiseptic and analgesic effects, while thymol demonstrates antifungal and antioxidant activities [35].

The aromatic ring of hydroxybenzenes can engage in hydrophobic interactions with diverse receptors, typically at an average distance of 3.7 Å. These interactions predominantly occur with phenylalanine residues (Supporting Information Table S7).

Hydrogen bonds are established with the aromatic hydroxyl groups of hydroxybenzenes. The calculated overall average HA distance is 2.4 Å, while the average DA distance is 3.2 Å (Supporting Information Table S7). These direct H-bonding interactions exhibit an average angle of 140°. In approximately 58% of cases, the hydrogen bond forms with the protein side chain, and no distinct amino-acid residue preference is observed for this interaction. Moreover, water-mediated hydrogen bonds are frequent, sharing similar characteristics, with average AW and DW distances to the water molecule of 3.2 Å. The angles DHW and AWD measure 141° and 91°, respectively.

Salt bridges can be established with 3-hydroxyphenylacetic acid (PDB ID 3HP) and 2-(3,4-dihydroxyphenyl)acetic acid (PDB ID DHY), both of which feature an aliphatic carboxyl moiety, as observed in multiple PDB structures (Supporting Information Table S1). These salt bridges consistently form with arginine, showcasing an average distance of 4.1 Å (Supporting Information Table S7).

With hydroxybenzenes, both P and T types of π-stacking interactions are almost equally prevalent (Supporting Information Table S7). Phenylalanine is the most common partner in T-type stacking, while histidine predominates in P-stacking. P-stacking displays an average distance between ring centers of 3.7 Å and an angle of 9°, whereas T-stacking exhibits a somewhat longer distance of 4.9 Å, coupled with an average angle of 79°. π-cation interactions are infrequent, with only five instances observed, formed between the aromatic ring and histidine or arginine residues.

Conversely, interactions with metal ions are prevalent, occurring in nearly all cases with iron and maintaining an average distance of 2.1 Å (Supporting Information Table S7). The coordination number varies between 4 and 6, with trigonal bipyramidal geometry representing the prevailing configuration. Hydroxybenzenes, being smaller and less sterically hindered than many other polyphenols, may facilitate their approach to and interaction with metal ions.

Catechol, an exemplary model polyphenol, effectively inhibits the urease enzyme in *Sporosarcina pasteurii* by binding to the interface between urease chains (PDB ID 5G4H) [36]. Catechol forms multiple hydrophobic interactions with chain C as well as an H-bond network including two waters that connects the ligand to the backbone of Ala336 on chain A (Figure S5).

## Stilbenes

Stilbenes constitute a class of polyphenolic compounds characterized by a shared 1,2-diphenylethylene backbone. These compounds are ubiquitously present in plants and boast diverse biological activities, encompassing antioxidant, anti-inflammatory, antitumor, antibacterial, and antiviral effects [37, 38]. Among the natural stilbenes, resveratrol and pterostilbene represent two of the most studied compounds due to their potential health benefits.

Resveratrol (3,5,4'-trihydroxy-trans-stilbene) is a stilbene monomer found in grapes, peanuts, berries, and various other plants. Numerous studies have underscored its ability to modulate several signaling pathways related to aging, inflammation, oxidative stress, apoptosis, autophagy, and metabolism [39]. Additionally, resveratrol has demonstrated significant chemopreventive and chemotherapeutic effects against various types of cancers by targeting multiple molecular pathways.

Pterostilbene (3,5-dimethoxy-4'-hydroxy-trans-stilbene) stands as a methylated derivative of resveratrol and is primarily present in blueberries and grapes. Its enhanced bioavailability and stability compared to resveratrol are attributed to the increased lipophilicity and resistance to metabolic oxidation [38].

Resveratrol is prominently featured in the PDB across a range of protein structures. In addition to resveratrol and its direct metabolites, the PDB database also includes structures with pterostilbene, piceatannol, and combretastatin A4 (Supporting Information Table S1). Stilbenes, featuring aromatic OH groups, exhibit a propensity for forming direct hydrogen-bonding interactions with no discernible preference for any residue type (Supporting Information Table S8). The average HA distance is 2.5 Å, while the average DA distance is 3.2 Å, forming at an average HDA angle of 134°. These hydrogen bonds are established predominantly with side-chain atoms (63% of cases), while the remaining were formed with backbone protein atoms.

Stilbenes demonstrate a relatively higher frequency of water-mediated hydrogen bonds compared to other polyphenol classes. This heightened occurrence can be attributed to the abundance of aromatic OH groups in stilbenes, arranged in a planar and rigid structure that aptly fits into protein grooves, interacting with numerous water molecules. The average AW distance measures at 3.4 Å, while the DW distance is 3.2 Å, with the average AHD of 139° and the average DHW angle of 94° (Supporting Information Table S8). Notably, there are no specific residue preferences observed for water-mediated hydrogen bonds.

Salt bridges involving stilbenes are uncommon, with sulfate resveratrol metabolites being the exclusive participants. These metabolites, featuring a negatively charged sulfate moiety, establish such rare salt bridges, and lysines represents the preferred interacting amino-acid residues (Supporting Information Table S8).

The aromatic and linker segments of stilbenes demonstrate the capability to engage in hydrophobic interactions, typically at an average distance of 3.7 Å (Supporting Information Table S8). This interaction is most frequently observed with phenylalanine or leucine residues.

π-stacking interactions with stilbenes are relatively infrequent, exhibiting no distinct preference for either T- or P-type stacking geometries (Supporting Information Table S8). Phenylalanine emerges as the predominant stacking partner. For T-stacking, the average distance and angle measure 5.1 Å and 75°, respectively; conversely, P-stacking maintains an average distance of 4.0 Å and an angle of 11°. π-cation interactions are even less prevalent, occurring in merely six unique PDB complexes and forming bonds with histidine or lysine. These interactions maintain an average distance of 4.6 Å. Notably, no complexes involving metal ions are observed with stilbenes.

NAD(P)H dehydrogenase, quinone 2 (NQO2), an enzyme facilitating the reduction of quinones to hydroquinones with flavin mononucleotide as a cofactor, plays a crucial role in diverse biological processes, including antioxidant defense, cell cycle regulation, and drug metabolism [40]. The crystal structure of FMN quinone reductase 2 in complex with resveratrol at high resolution exemplifies the binding within the interface of two chains (Figure S6). A water-mediated H-bond network is established, connecting the aromatic OH group of resveratrol to the side-chain of Thr71A and the backbones of Leu120A and Gln112A. Direct H-bonds are also observed between the backbone of Gly174A and the side-chain of Asn161B with resveratrol, while Phe178A engages in a π-stacking interaction with the resorcinol moiety. Additionally, numerous hydrophobic interactions occur with the aromatic rings.

## Coumarins

Coumarins, derived from 1-benzopyran-2-one, represent a class of natural compounds extensively found in various medicinal plants [41, 42]. They manifest a diverse range of pharmacological activities, including anti-inflammatory, anticoagulant, anticancer, antibacterial, antimalarial, antifungal, antiviral, neuroprotective, and antidiabetic effects. Aesculetin, scopoletin, and umbelliferone, among several coumarins, have emerged as pivotal derivatives that gained significant attention in medicinal chemistry and pharmacology.

In general, coumarins exhibit the capability to engage in hydrophobic interactions due to their condensed two-ring aromatic system. The lactone moiety, as well as aromatic hydroxyl groups attached to the ring system, allows for the formation of both direct and water-mediated H-bonds. For instance, in the case of umbelliferone (7-hydroxycoumarin), the ring system is also involved in T-type and P-type π-stacking interactions (Supporting Information Table S9).

The recently published structure of the COVID-19 nonstructural protein 3 (PDB id 5RT5), crucial for virus replication and inhibited by umbelliferone, provides instances of the aforementioned coumarin interactions [43] (Figure S7).

# S2. Binding Patterns of Polyphenols with with Low Representation in the PDB

## Coumestans

Coumestans constitute a class of phytochemicals, featuring an oxygen heterocyclic four-ring system that combines a coumarin moiety and a benzofuran moiety linked by a C=C bond [44]. These compounds are widespread in various plants, particularly legumes, and exhibit estrogenic and other biological activities. Among them, coumestrol stands out as one of the most abundant and extensively studied coumestans, commonly isolated from soybeans or alfalfa sprouts. Coumestrol functions as a phytoestrogen capable of binding to both estrogen receptor alpha and estrogen receptor beta [45]. Additionally, it inhibits the activity of aromatase and 3α-hydroxysteroid dehydrogenase, two enzymes pivotal in the biosynthesis of steroid hormones [46].

Within PDB structures, coumestrol is found in co-crystallized forms, binding to estrogen receptors, 17beta-hydroxysteroid dehydrogenase, and protein kinase CK2 (PDB ID 6HNW, Supporting Information Table S1, Figure S8a) [47]. The expansive aromatic condensed planar four-ring system facilitates numerous hydrophobic interactions (Supporting Information Table S10). OH and lactone moieties exhibit the ability to form direct or water-mediated H-bonds. π-stacking interactions, occurring in both P and T types, are evident with the ring system, while π-cation interactions are not observed.

## Lignans

Lignans, characterized by a common 1,4-diarylbutan skeleton, represent a class of natural compounds extensively distributed in plants, showcasing diverse biological activities [48]. These compounds have garnered significant attention due to their potential health benefits, particularly as phytoestrogens capable of modulating estrogen receptor signaling and hormone metabolism [48, 49].

Pinoresinol, found abundantly in plants like sesame seeds, Brassica vegetables, and olive oil, stands out as one of the most prevalent lignans [49, 50]. Its reported pharmacological effects encompass a spectrum of activities, including antioxidant, anti-inflammatory, anti-diabetic, anti-obesity, anti-cancer, and neuroprotective properties [51–54]. The mechanisms underlying pinoresinol's actions involve the modulation of multiple signaling pathways, including NF-κB and Nrf2 [54].

PDB structures encompass a diverse array of lignans, featuring well-known entities such as pinoresinol, (-)3,4-divanillyltetrahydrofuran, secoisolariciresinol, arctigenin, lariciresinol, and matairesinol (Supporting Information Table S1). These compounds exhibit variations in the linker section of the molecule; for instance, pinoresinol incorporates a tetrahydrofuran group, while secoisolariciresinol includes a more flexible butane-1,4-diol group. Additionally, distinctions arise in the degree of hydroxylation and methylation.

Overall, the butane linkers, along with the two aromatic rings, establish hydrophobic interactions with the receptor (Supporting Information Table S11). Direct or water-mediated hydrogen bonds emerge between aromatic OH and ether groups, and when present, linker OH groups participate in forming direct or water-mediated H-bonds. Moreover, both aromatic rings demonstrate the propensity to engage in π-stacking interactions, predominantly of the T-type.

An illustrative example found in the PDB is the complex of pinoresinol-lariciresinol reductases bound to lariciresinol (PDB ID 7CSE) (Figure S8) [55]. This enzyme plays a crucial role in lignan biosynthesis following the initial dimerization of two monolignols. Notably, direct or water-mediated hydrogen bonds are observed to form with the hydroxyl groups of the aromatic rings. Additionally, the aromatic rings exhibit T-type π-stacking interactions with phenylalanine. Moreover, the linker region of the ligand engages in hydrophobic interactions with the enzyme.

Naphthoquinones

Naphthoquinones are widely distributed in nature, prevalent in various plants [56]. Notably, juglone, a 1,4-naphthoquinone derivative found in plants of the Juglandaceae family such as walnut and hickory, has been extensively studied for its remarkable pharmacological properties [57]. Juglone exhibits a range of biological effects, including anti-inflammatory, antioxidant, antidiabetic, anticancer, and antitrypanosomal activities [57–59]. Juglone also represents a versatile scaffold for the synthesis of new derivatives with improved pharmacological profiles.

From the PDB, we obtained complexes featuring juglone, menadione, or plumbagin (Supporting Information Table S1). The condensed two-ring aromatic system of these compounds engages in hydrophobic interactions, π-stacking, salt-bridges or π-cation interactions (Supporting Information Table S12). Additionally, the quinone moiety, along with the aromatic hydroxyl group, can establish direct or water-mediated H-bonds as seen in the example of juglon bound to Ribonuclease P 1 (PDB ID 6BV9, (Figure S8c) [60].

## Curcuminoids

Curcuminoids, a class of plant-based antioxidants derived from turmeric *Curcuma longa*), a member of the ginger family, feature a chemical structure known as a linear [61]. This structure comprises two aromatic rings connected by a seven-carbon chain. Among the prominent curcuminoids is curcumin, which has gained significant attention for its potential therapeutic benefits against various diseases, including arthritis, cancer, diabetes, neurodegenerative disorders, and inflammatory conditions [62].

In examining the PDB, we note instances of curcumin binding to human dual specificity tyrosine-phosphorylation-regulated kinase 2 or transthyretin (Supporting Information Table S1). Interestingly, two tautomeric forms of curcumin are observed – both the keto and enol forms. At physiological conditions, curcumin predominantly exists in the enol form [63].

As anticipated, the extended linker chain and aromatic rings of curcuminoids engage in numerous hydrophobic interactions with protein receptors (Supporting Information Table S13). Hydrogen bonds can form with the hydroxyl, ether, and keto moieties present in the structure. Notably, in dual specificity tyrosine-phosphorylation-regulated kinase 2 (PDB ID 6HDR) [64], curcumin forms π-stacking interactions of the P-type with phenylalanine. Additionally, the high-resolution structure with transthyretin (PDB ID 4PME) [65], reveals, alongside hydrophobic contacts, an array of direct and water-mediated hydrogen bonds across the ligand (Figure S8d).

## Chalcones

Chalcones constitute a class of organic compounds characterized by a shared structural motif featuring two aromatic rings connected through a three-carbon α, β-unsaturated carbonyl system. Notably, chalcones serve as precursors for the synthesis of flavonoids and isoflavonoid [66].

Demonstrating an ability to modulate various molecular targets and signaling pathways implicated in inflammation, cancer, neurodegeneration, and metabolic disorders, chalcones have proven versatile in pharmacological applications. For instance, they can hinder NF-κB activation, impede glucose uptake, induce apoptosis, inhibit histone acetyltransferases, enhance SIRT1 activity, and impact amyloid-β permeability [67–69].

Recognized as a privileged scaffold for drug discovery, chalcones offer promising avenues for therapeutic development [66].

Notable examples include isoliquiritigenin, which inhibits NF-κB activation by suppressing IKK, ERK1/2, and p38 phosphorylation; [70] phloretin, capable of blocking glucose uptake by inhibiting GLUT1 [71]; and bavachalcone which can induce apoptosis in leukemia cells by activating caspases and PARP cleavage [72].

We identified 11 PDB complexes featuring a chalcone bound to a protein binding site, with isoliquiritigenin being the predominant ligand (Supporting Information Table S1). Additionally, representations of phloretin, bavachalcone, and butein were observed. A common occurrence involves hydrophobic interactions between the aromatic ring systems and/or linkers of chalcones and hydrophobic amino-acid residues (Supporting Information Table S14). Due to their relatively large structure, chalcones can accommodate numerous aromatic OH groups arranged at a distance, facilitating the formation of direct or water-mediated H-bonds. Moreover, interactions involving the C=O moiety of the linker are also observed. In most cases, the trans conformation of chalcones is more stable [66]; consistently, trans isomers are observed in all PDB structures featuring chalcones with a conjugated linker. Moreover, chalcones are noted for forming T-type π-stacking interactions with aromatic rings, primarily with phenylalanine or tyrosine residues, and π-cation interactions with arginine or histidine.

The high-resolution structure with PDB ID 1FP1 illustrates a typical chalcone, isoliquiritigenin, bound to the plant O-methyltransferase [73]. This complex reveals a distinctive water-mediated H-bond network, prominently involving His278A and Asn297A. Additionally, a direct hydrogen bond is established with the side chain of Thr32B, while a π-stacking interaction occurs with Phe135A (Figure S8e).

## Nonclassified polyphenols

Within the category of nonclassified polyphenols, ellagic acid and mandelic acid stand out as the most represented (Supporting Information Table S1). Ellagic acid, a dilactone hexahydroxydiphenic acid, features four condensed rings and has been documented for its diverse pharmacological activities, including antioxidant, anti-inflammatory, antimutagenic, and antiproliferative effects [74]. Moreover, it demonstrates hepatoprotective properties by modulating oxidative stress, inflammation, lipid metabolism, fibrosis, and carcinogenesis in the liver [75]. Ellagic acid has further exhibited antimutagenic activity by preventing DNA damage induced by various mutagens, such as aflatoxin B1, or by enhancing DNA repair mechanisms [75, 76].

On the other hand, mandelic acid, a chiral aromatic alpha hydroxy acid present in bitter almonds, is represented in the PDB in both enantiomeric forms (Supporting Information Table 1). Recognized for its antibacterial, anti-inflammatory, and antioxidant properties, mandelic acid also finds application in the treatment of conditions like acne, melasma, wrinkles, and other skin-related issues [77].

Both ellagic acid and mandelic acid exhibit the capacity to form numerous direct or water-mediated hydrogen bonds involving their polar oxygen groups (Supporting Information Table S15). Additionally, the carboxylic moiety of mandelic acid facilitates the formation of salt bridge interactions. Furthermore, there are instances where mandelic acid coordinates with magnesium or cobalt ions (e.g. PDB ID 3TTE, 3ZGJ). In the case of ellagic acid, its large ring system is involved in π-stacking interactions, predominantly of the P-type.

The high-resolution structure represented by PDB ID 1MDL features the bacterial mandelate racemase mutant enzyme cocrystallized with mandelic acid (Figure S8f) [78]. In this complex, the ligand engages in diverse interactions, including two hydrophobic bonds with aromatic amino-acid residues, three direct hydrogen bonds, and a water-mediated hydrogen bond with the mutated Arg166 residue. The benzene ring of the ligand forms a T-type π-stacking interaction with histidine 297, while its carboxilate moiety establishes a salt bridge with lysine 164. Additionally, the carboxylate moiety of mandelic acid participates in metal complexation with a Mg^2+^ ion.

# S3. Helical Regions Around the XEG Binding Site

We examined the structural changes in the helical regions of epicatechin-3-gallate bound to the glutamate dehydrogenase system (PDB ID: 6DHL, ligand XEG) [79]. Three medoid cluster structures were superimposed to reveal two distinct conformations, with a clear transition between them. Notably, after molecular dynamics simulations, conformational changes were observed in the α-helical regions (Figure S10). The XEG binding site is surrounded by loops, with three α-helices in direct contact with the ligand. The first helix (Ser100-Val118) was displaced by 2.02 Å, the second helix (Asp476-Glu495) by 1.61 Å, and the third helix (Pro202-Gly206) by 0.75 Å. Despite these displacements, the overall structure of the glutamate dehydrogenase system remains intact, with the binding site and ligand pose preserved, maintaining the volume and integrity of the ligand binding site.

# S4. Density-Based Clustering Analysis of Waters of the GDH-XEG System using MADE

To further support the stability of identified binding poses and water bridges, we performed a density-based clustering analysis of waters through time (MD trajectory) using MADE (or ProBiS H2O) method [80, 81]. As a proof-of-concept, the protein conformation with highest occupancy (epicatechin-3-gallate bound to glutamate dehydrogenase protein conformation - main trajectory 0-600 ns; also found in replica 226 ns to the end) was selected and 61 snapshots collected, aligned and TIP3P water molecules clustered using 3D-DBSCAN. We selected the binding site with XEG ligand (name, avg x, y, z, min x, max x, min y, max y, min z, max z; box 4.0 A around extremes): ['XEG.601.P', 3.86834, -1.83824, 4.91942, 0.682, 9.076, -8.25, 3.946, 2.08, 11.32] and identified 11 clusters with 16 or more TIP3P O-heteroatoms that span in conservation from 0.26 to 0.84. Conserved TIP3P (O) water clusters were identified at the key water-mediated hydrogen bonds, namely bridging ligand contacts towards Asp119, Glu487, Hsd85, Arg86, Lys387, Asn388, His209 and Ser393. In this manner we can observe the described water network even through complete conformation-sampling time.

**Table S1.** Polyphenols identified from the Protein Data Bank

| **PDB Ligand ID** | **Name** | **Class** | **Structure** | **Present in PDBS** |
| --- | --- | --- | --- | --- |
| 07L | 7-hydroxycoumarin | coumarin | 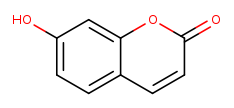 | 5LNI, 5LNJ, 8DQQ, 3U0F, 5RT5 |
| 0XE | Baicalin | flavonoid | 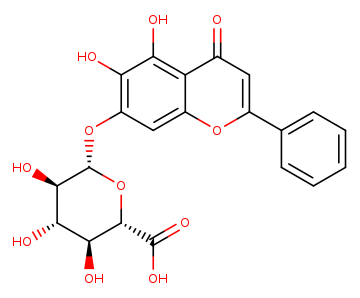 | 4H2B, 4Q2V |
| 0XR | ethyl caffeate | hydroxycinnamic acid derivative | 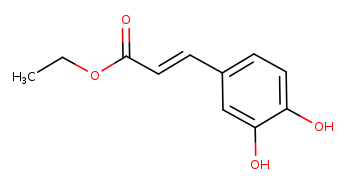 | 4PWG, 4GQQ |
| 13X | Phloroglucinol | hydroxybenzene | 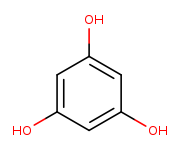 | 5MG5, 6BZA, 4HDK, 6ZZX |
| 15W | 7-hydroxy-4-methoxyflavone | flavonoid | 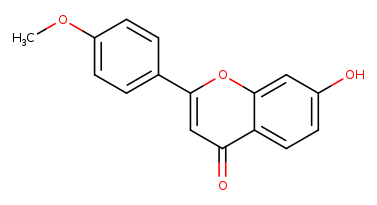 | 4HL5 |
| 15Z | 7,3,4-trihydroxyflavone | flavonoid | 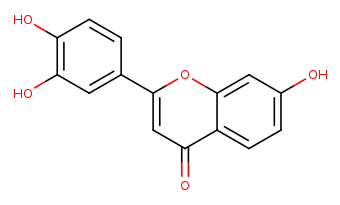 | 4HLF |
| 16S | 3,4-dihydroxyflavone | flavonoid | 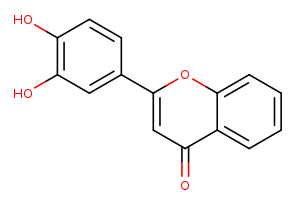 | 4HLM |
| 1HN | 1-hydroxy-2-naphthoic acid | other | 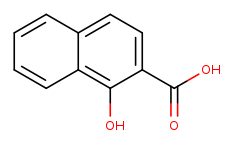 | 3NKT, 6ZGR |
| 28E | epicatechin | flavonoid | 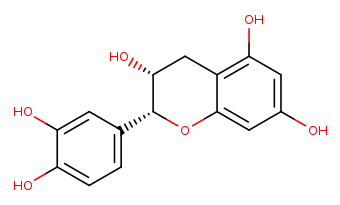 | 6AWV, 6AWT, 6AX0, 4MA6 |
| 2A7 | 5-Methoxyresorcinol | hydroxybenzene | 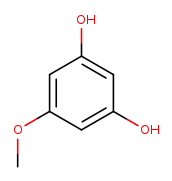 | 2YE5 |
| 2HC | o-coumaric acid | hydroxycinnamic acid derivative | 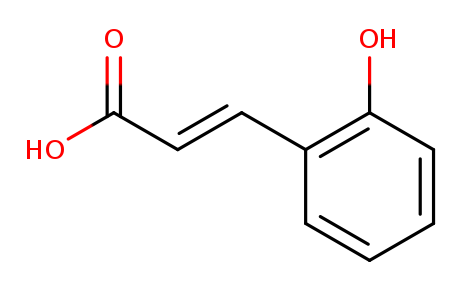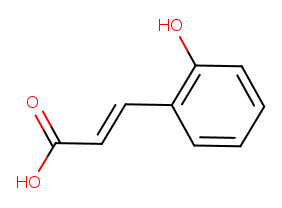 | 1V5Z, 5BNL |
| 34D | 3,5-dihydroxybenzoic acid | phenolic acid | 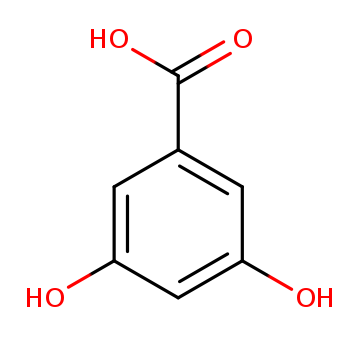 | 2BX7 |
| 3DM | syringol | hydroxybenzene | 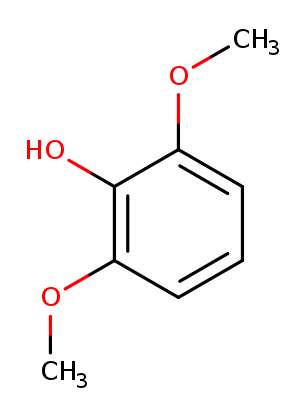 | 5OMU, 6HQQ, 6HQS, 6HQR, 6HQT, 3FU8, 3FU7, 5OY2, 3VXJ |
| 3HB | 3-hydroxybenzoic acid | phenolic acid | 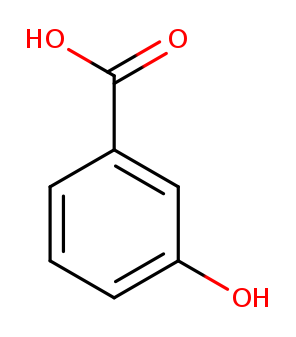 | 5HPI, 4BK1, 4Q6W, 4PAI, 2DKH, 3TUS, 3PCB, 7TRW, 5A3K, 7X2Y, 6SJ3, 6O9A, 5I7I |
| 3HP | 3-hydroxyphenylacetic acid | hydroxybenzene | 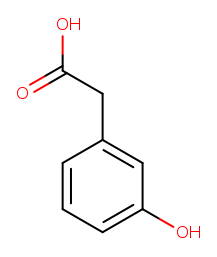 | 5RTG, 3PCE, 5IZZ |
| 3L9 | montbretin A | flavonoid/hydroxycinnamic acid derivative | 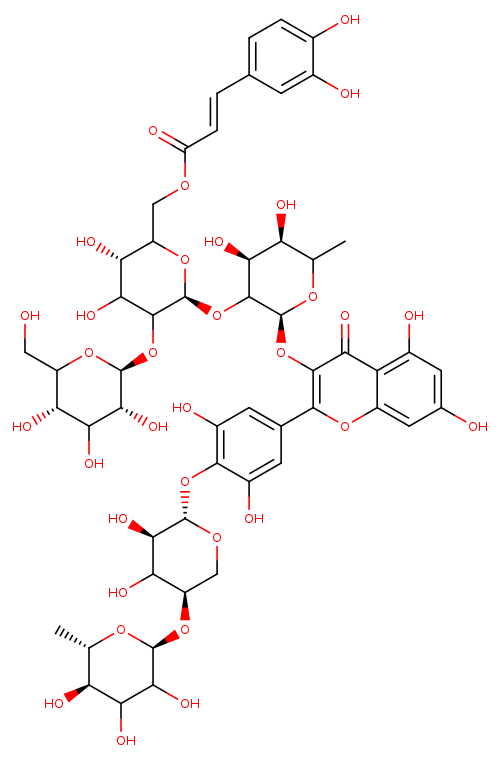 | 4W93 |
| 3RL | pterostilbene | stilbene | 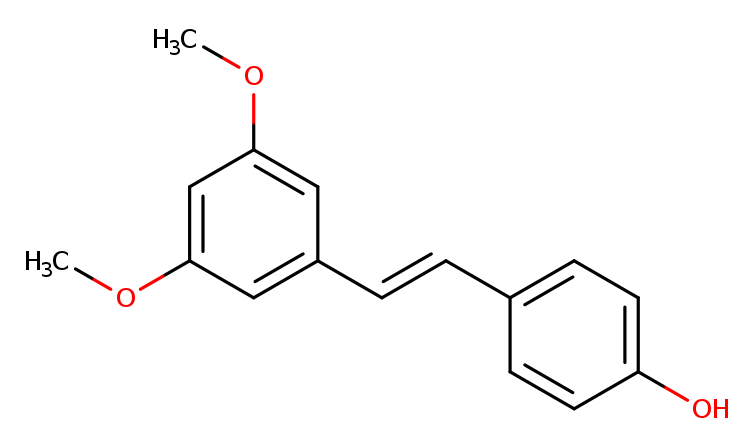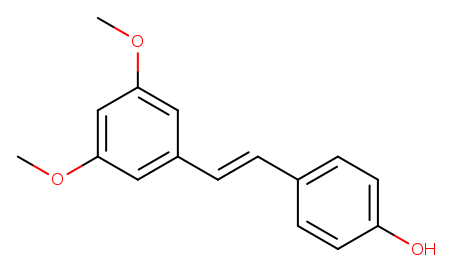 | 6JEN, 8IE7, 7WAS, 4WNS |
| 3WL | baicalein | flavonoid | 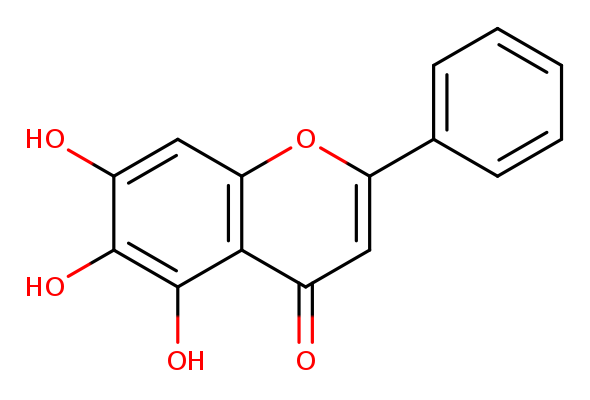 | 7XAX, 6M2N, 8A0Q, 4X2A |
| 47X | 7,3,4-trihydroxyisoflavone | flavonoid | 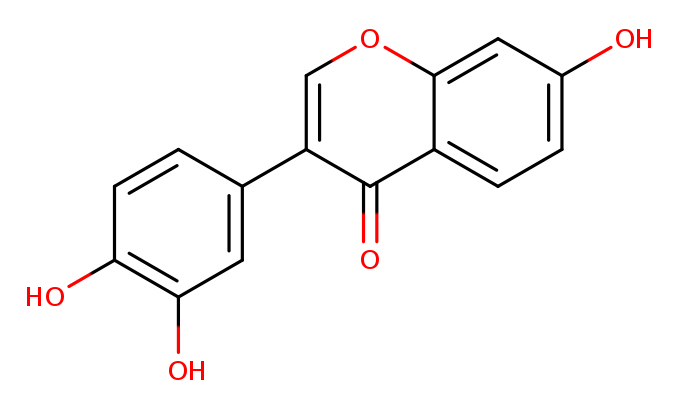 | 3L5R, 5AV0 |
| 4FE | isoferulic acid | hydroxycinnamic acid derivative | 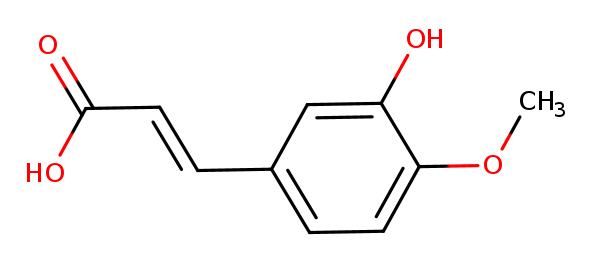 | 3CBG |
| 4HC | 4-hydroxycoumarin | coumarin | 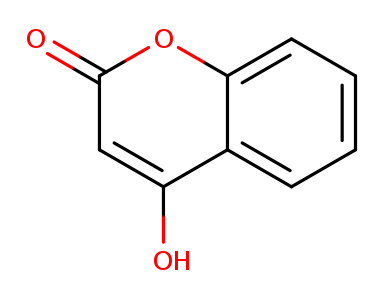 | 1V5Y, 6Y1U, 6Y1V |
| 4LV | α-methyl cinnamic acid | hydroxycinnamic acid derivative | 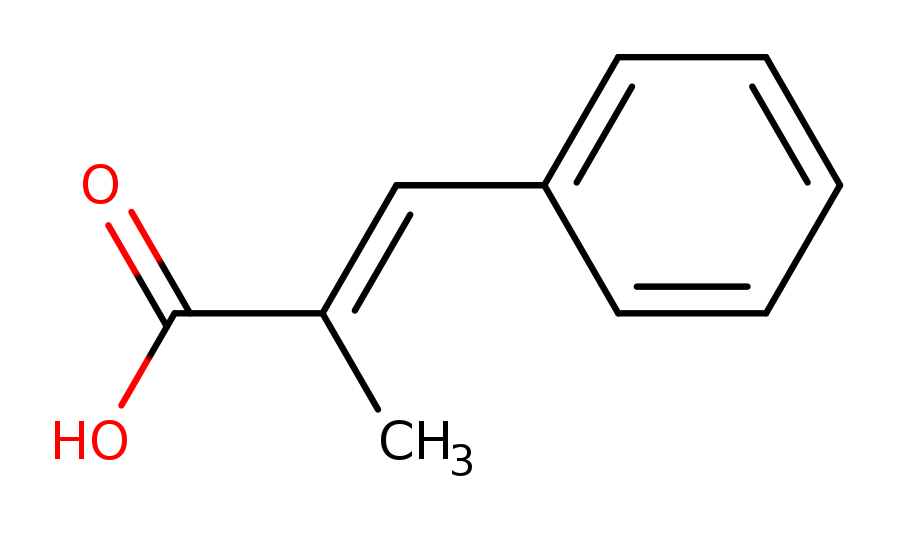 | 4ZA7 |
| 57D | chrysin | flavonoid | 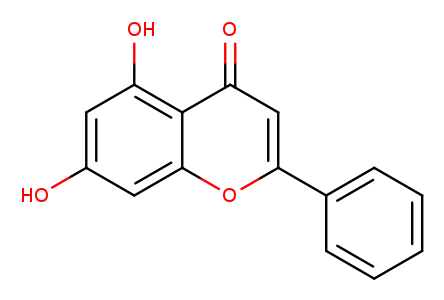 | 4DES, 3EBO, 7D38 |
| 5J7 | mini-montbretin A | flavonoid/hydroxycinnamic acid derivative | 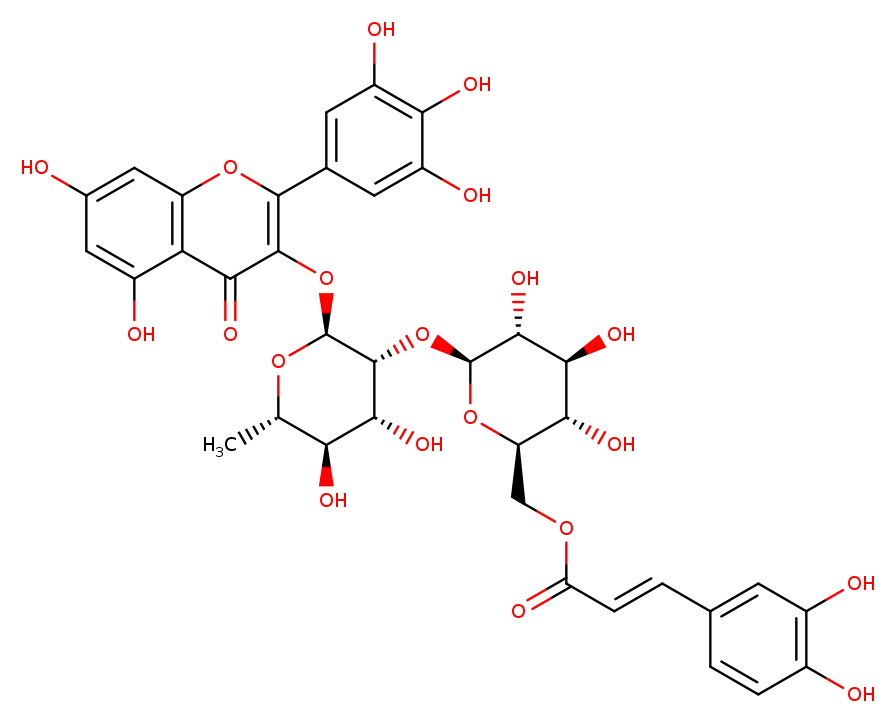 | 5E0F |
| 674 | 6-Hydroxydaidzein | flavonoid | 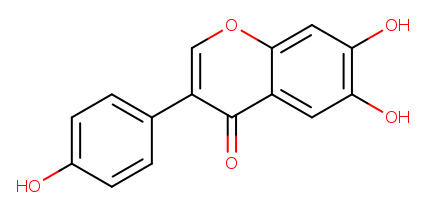 | 7EBW |
| 6B5 | luteolin-MeO | flavonoid | 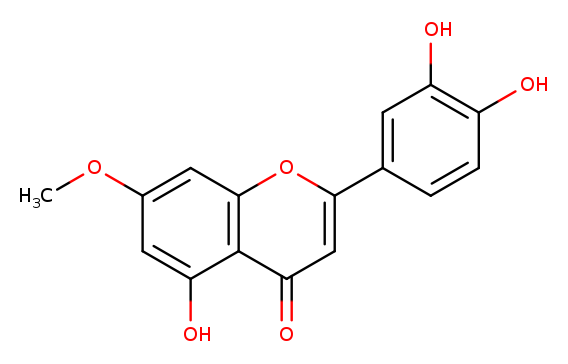 | 5IHH |
| 6BK | 5,3,4-Trihydroxyflavone | flavonoid | 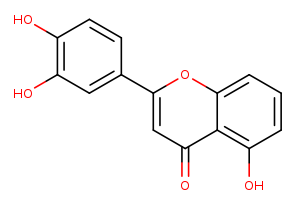 | 5IID |
| 6J6 | (2R)-2-(3,4-dihydroxyphenyl)-6-hydroxy-2,3-dihydrochromen-4-one | flavonoid | 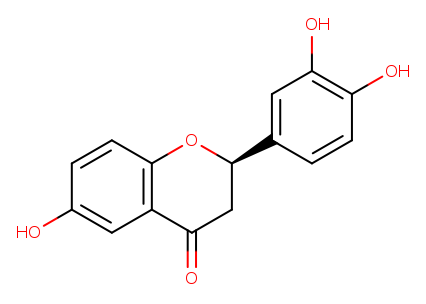 | 5L42 |
| 6JM | 3,6,3,4- tetrahydroflavone | flavonoid | 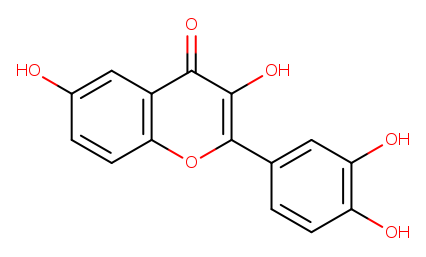 | 5JCJ |
| 6JP | hesperetin | flavonoid | 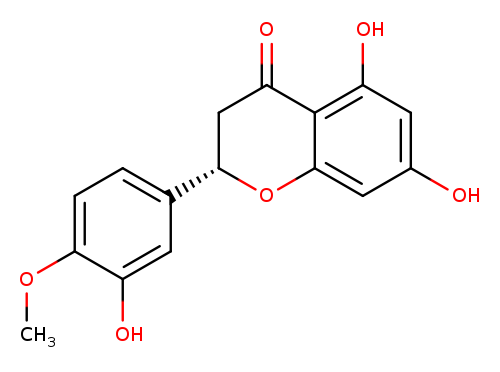 | 7POU, 5JDC |
| 7BA | combretastatin A4 | stilbene | 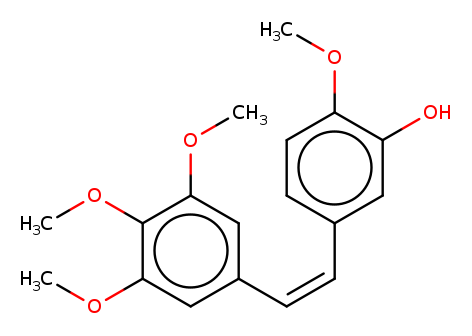 | 5LYJ |
| 7DV | methylhydroquinone | hydroxybenzene | 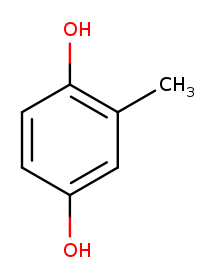 | 5M26 |
| 7WR | 3-O-methylgallate | phenolic acid | 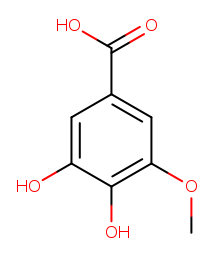 | 5X1K |
| 83X | phloracetophenone | hydroxybenzene | 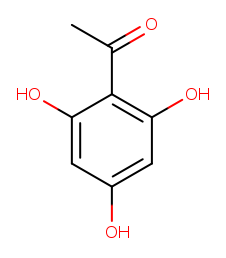 | 5XEY |
| 88R | 2-[(3,4-dimethoxyphenyl)methyl]propanedioic acid | hydroxycinnamic acid derivative | 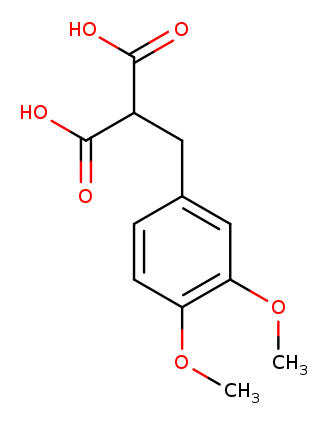 | 4AJJ, 4AJI |
| 8IB | 2-hydroxyterephthalic acid | phenolic acid | 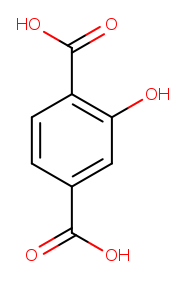 | 7Q06 |
| 8KZ | pinostilbene | stilbene | 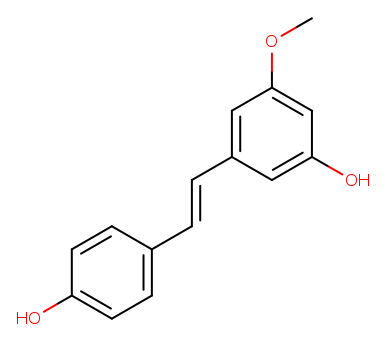 | 8IE6, 7WAR |
| 8MO | methoxsalen | coumarin | 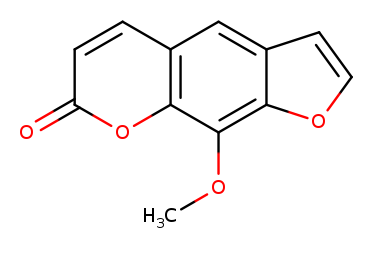 | 1Z11 |
| 90R | plumbagin | naphthoquinone | 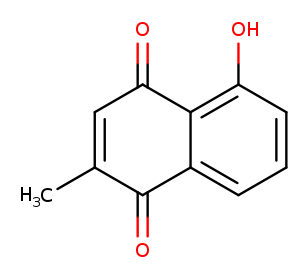 | 7CA1 |
| 9EQ | 1-[2,6-bis(oxidanyl)phenyl]ethanone | hydroxybenzene | 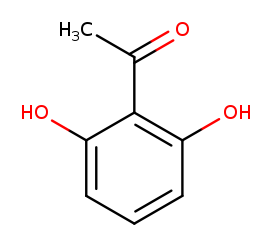 | 7NM0 |
| 9PS | 4-[(~{E})-2-naphthalen-2-ylethenyl]benzene-1,2-diol | other | 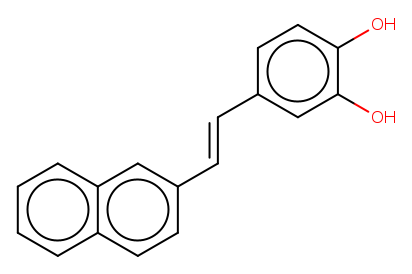 | 7Q9N |
| 9RJ | 4-[(~{E})-2-naphthalen-1-ylethenyl]benzene-1,2-diol | other | 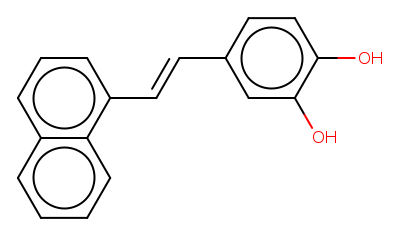 | 7Q9L |
| 9WZ | 2-hydroxy-4-(2-hydroxyethyl)phenyl beta-D-fructofuranoside | other | 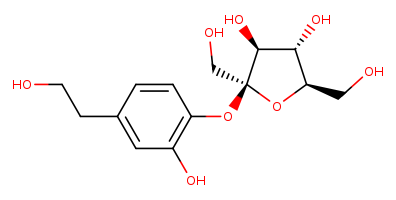 | 5O47 |
| AFE | afzelin | flavonoid | 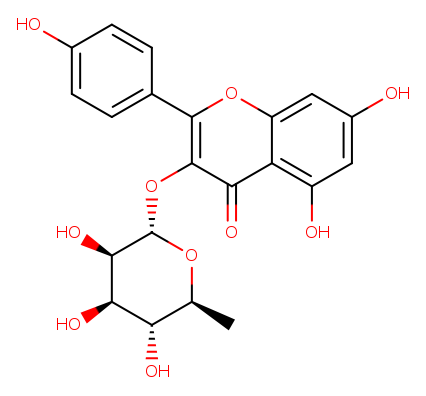 | 4EL9 |
| AGI | apigenin | flavonoid | 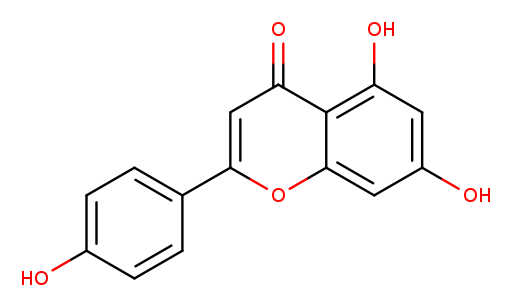 | 4HKK, 4DGM, 5I2H, 5AUV, 4DER, 7D3A, 3AMY, 4WO0, 3CF9, 5UQT |
| B0E | 1,3-bis(oxidanyl)benzo[c]chromen-6-one | coumarin | 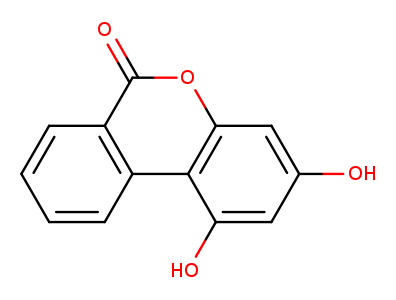 | 5OWE |
| B7S | 5,7-dihydroxychromone | other | 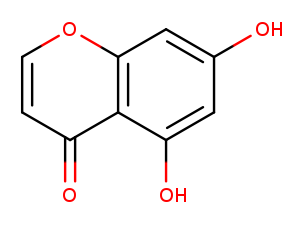 | 5WP1 |
| BHQ | 2,5-di-tert-butylhydroquinone | hydroxybenzene | 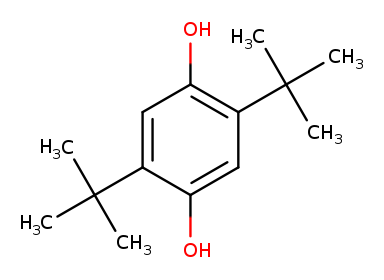 | 2AGV |
| BIK | 3,7-dihydroxynaphthalene-2-carboxylic acid | other | 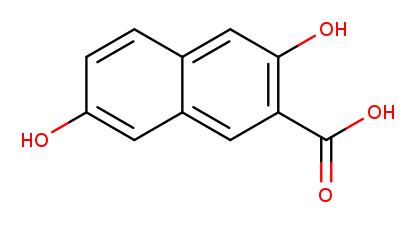 | 1U5A, 1U5C |
| BUN | butein | calchone | 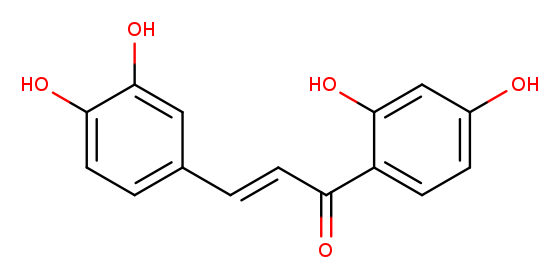 | 4RLW |
| BVL | (2E)-1-[2-hydroxy-4-methoxy-5-(3-methylbut-2-en-1-yl)phenyl]-3-(4-hydroxyphenyl)prop-2-en-1-one | calchone | 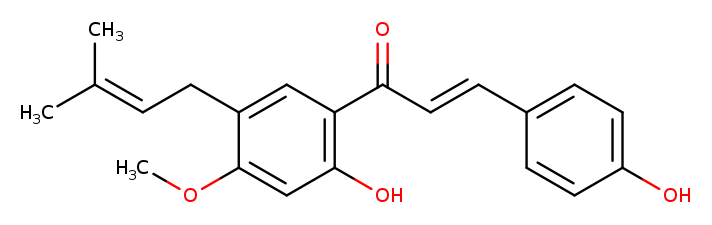 | 2ZBH |
| BZJ | 3-Hydroxy-2-naphthoic acid | other | 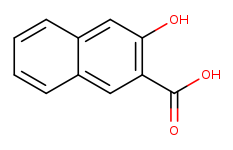 | 7NN4, 7YY7, 6AWW, 6SQ9 |
| CAQ | catechol | hydroxybenzene | 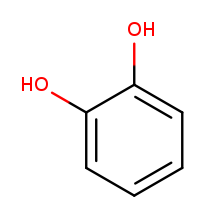 | 2BUY, 2BUQ, 4QON, 3HHY, 2PUM, 4ZXT, 5KBI, 3O4M, 6S2H, 1DLT, 5SWG, 7WKM, 7WKL, 1XEP, 3FW4, 4K7I, 7KK0, 7WJR, 3T67, 7WMB, 1KND, 7BP1, 5VXT, 3MI5, 5G4H, 4OOW |
| CC6 | datiscetin | flavonoid | 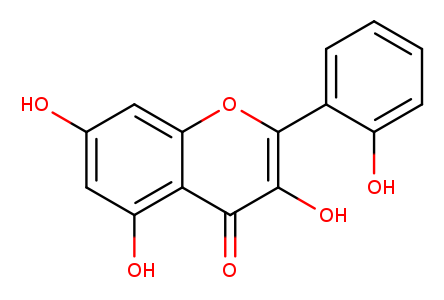 | 5JCX |
| CC9 | curcumin keto form | curcuminoid | 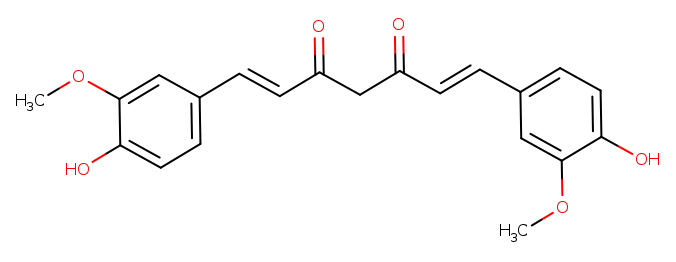 | 6HDR |
| CGG | chlorogenic acid | hydroxycinnamic acid derivative | 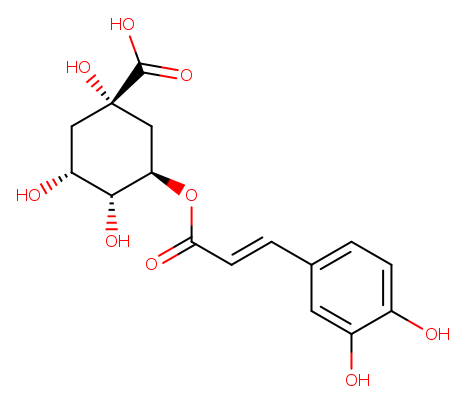 | 5MCB |
| CIY | coniferaldehyde | hydroxycinnamic acid derivative | 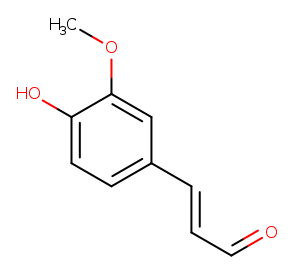 | 6KLJ, 3P9K, 4WGG, 4HFN, 6HOT, 6HOP, 5FXE |
| COU | coumarin | coumarin | 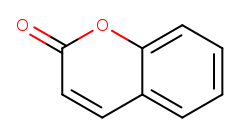 | 2PMJ, 2H90, 3L5M, 3L68, 3L66, 3CRB, 1Z10, 4UTI, 4UTL, 2PWB |
| CUE | coumestrol | coumestan | 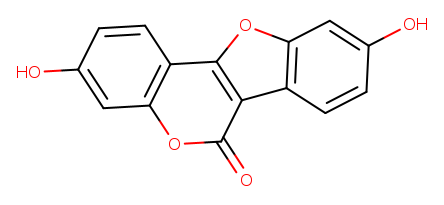 | 4FIZ, 3QWI, 5KR9, 6HNW |
| CUR | curcumin, enol form | curcuminoid | 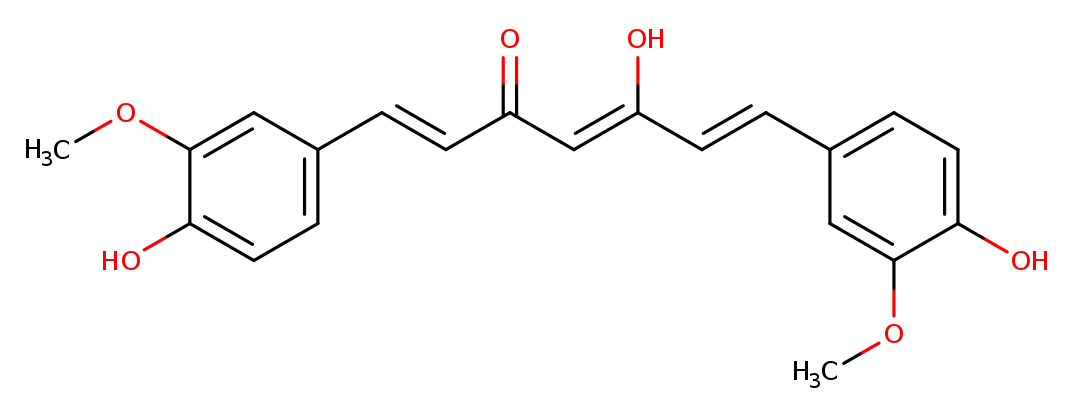 | 4PMF, 4PME, 5ZTN |
| CWE | naringenin | flavonoid | 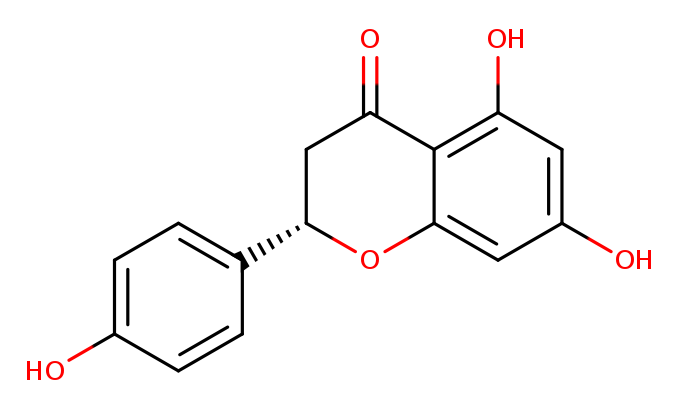 | 7DQM, 6G8H, 6F71, 6SRA |
| D2M | 4-(2,6-dimethoxyphenoxy)-2,6-dimethoxyphenol | hydroxybenzene | 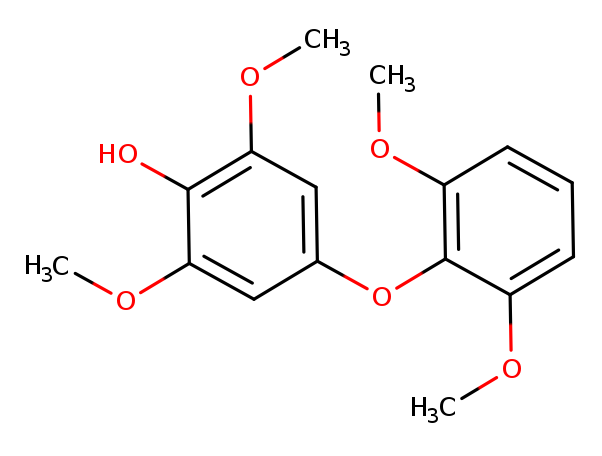 | 3FU7 |
| D75 | daidzein-7-O-glucuronide | flavonoid | 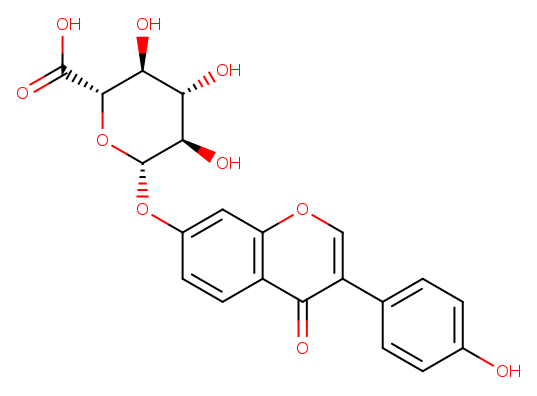 | 5AL8 |
| DBH | 2,3-dihydroxybenzoic acid | phenolic acid | 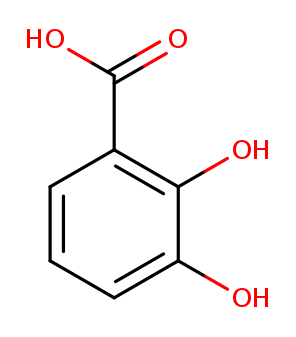 | 3TF6, 1MDB, 1MD9, 4E5L, 1L6M, 3T1D, 3I0A, 3BY0, 3K3L, 3U0D, 3CMP, 3SAO, 2WHY |
| DDC | 7-hydroxyflavanone | flavonoid | 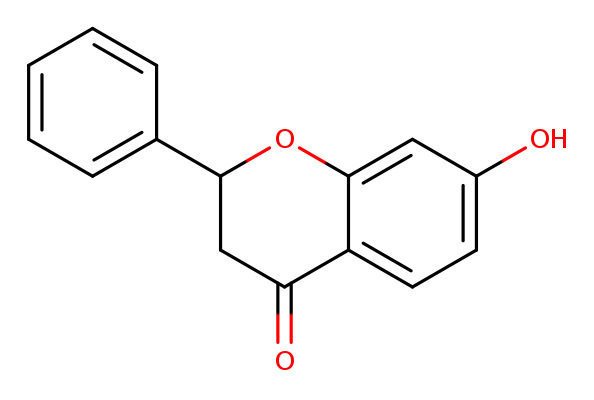 | 1FM8 |
| DFL | 4-Hydroxyflavanone | flavonoid | 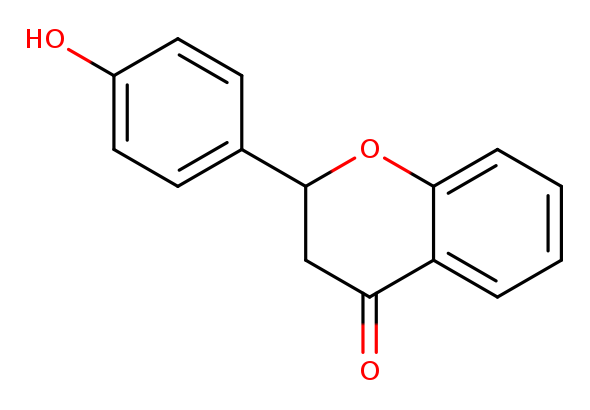 | 4KZQ, 1JEP |
| DFV | 5-deoxyflavanone | flavonoid | 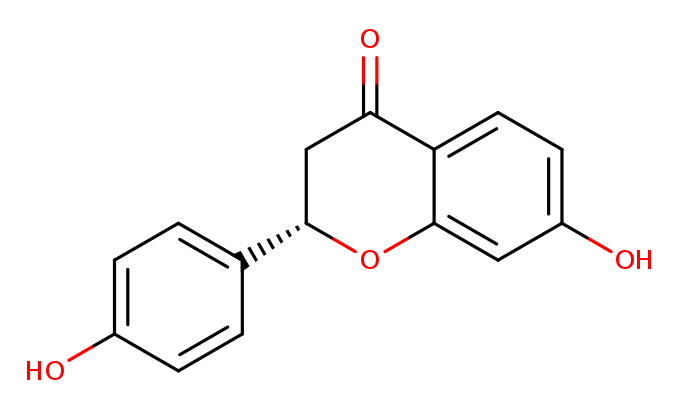 | 1FM7, 1JX1, 1JX0 |
| DHB | 3,4-dihydroxybenzoic acid | phenolic acid | 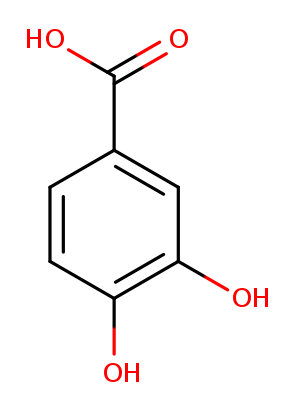 | 2BUV, 1N8Q, 6QHN, 1PHH, 4J0I, 3O5U, 4FHT, 1YKP, 1YKN, 3LMX, 3HKP, 4EYK, 2BSL, 3WRC, 3DX5, 6JU1, 3PCA, 7CVX, 1B4U, 1YKL, 5FAN, 4RGX, 4PAF, 3MV6, 2BV0, 7EQV, 1EOB, 5X1M, 5X1N, 6S33 |
| DHC | caffeic acid | hydroxycinnamic acid derivative | 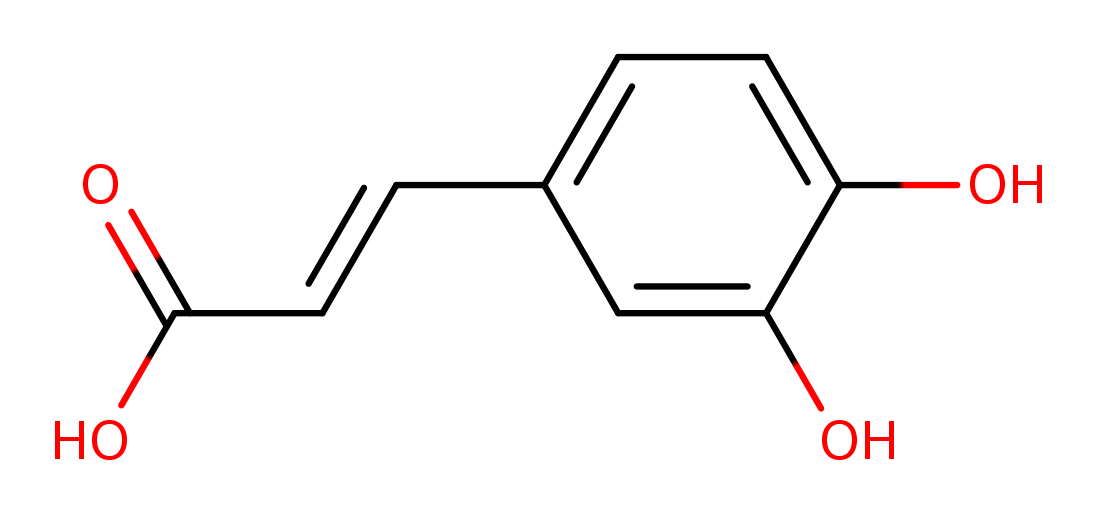 | 3HOF, 2O7D, 4FB4, 1KOU, 6YRI, 4YU7, 6AWU, 4EYQ, 5VFJ, 6I72, 7NRR, 7YEN, 3S2Z, 4N0S |
| DHY | 2-(3,4-dihydroxyphenyl)ethanoic acidhydroxybenzene | hydroxybenzene | 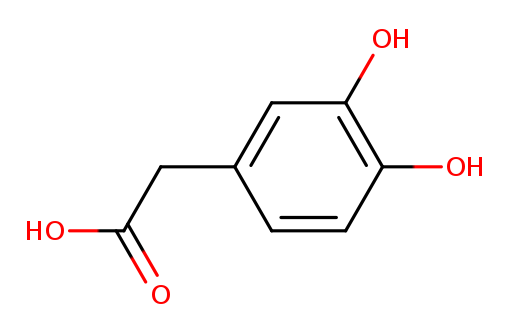 | 5BWH, 1Q0C, 1F1V, 4GHD, 3PCN, 4GHG, 3MFL, 4Z6O, 4Z6P, 4Z6Q, 1AI4 |
| DLM | delphinidin | flavonoid | 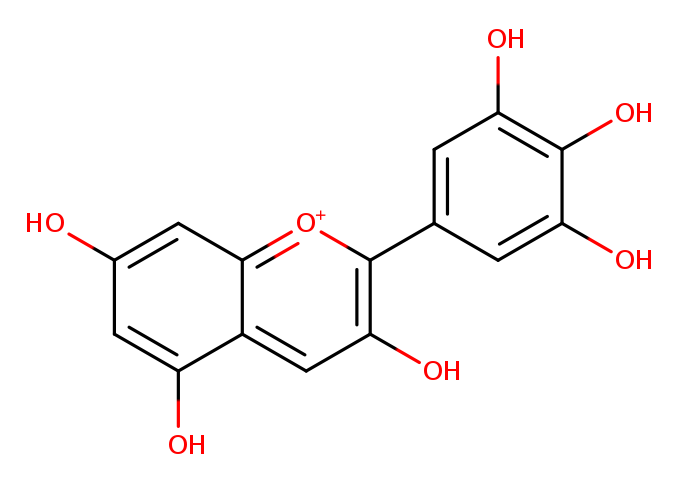 | 4REM |
| DNA | 1,4-dihydroxy-2-naphthoic acid | naphthoquinone | 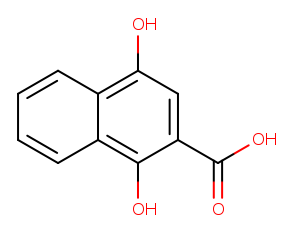 | 6O04, 6O0G, 6O0J, 6O0N, 3I64 |
| DOB | 2,4-dihydroxybenzoic acid | phenolic acid | 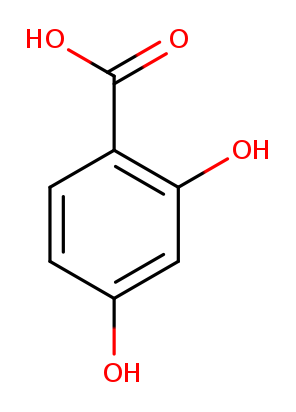 | 1PBB, 1DOD, 1DOE |
| DQH | taxifolin | flavonoid | 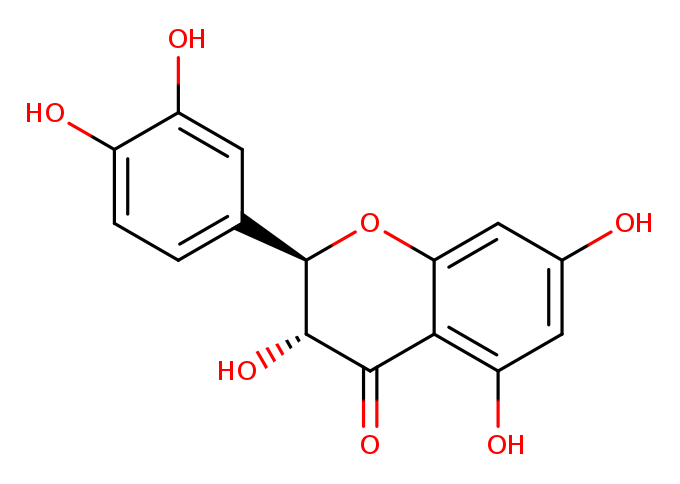 | 8B7Z, 8B7U, 2C29, 1GP5, 3TVQ |
| DZN | daidzin | flavonoid | 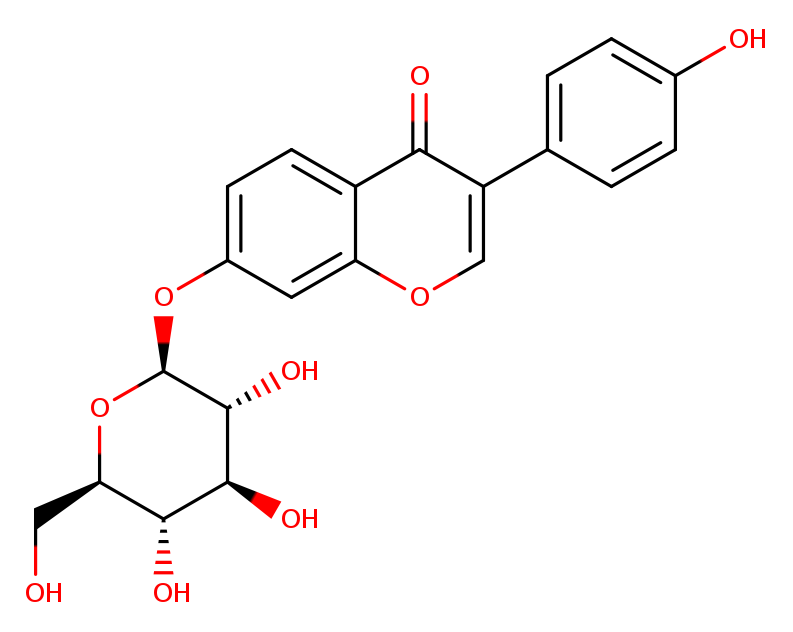 | 2VLE |
| E35 | ethyl 3,5-dihydroxybenzoate | hydroxybenzene | 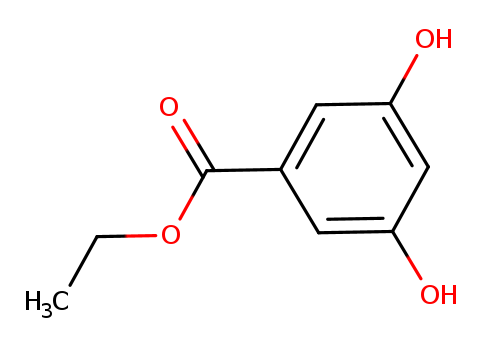 | 4J0J |
| EGT | epigallocatechin | flavonoid | 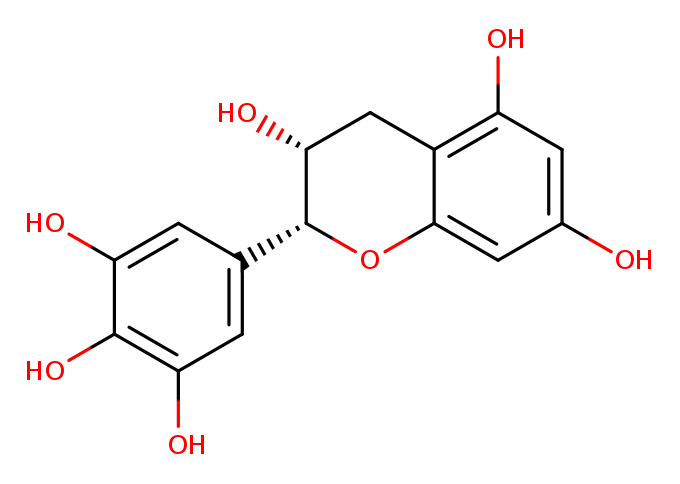 | 1JNQ |
| EOL | eugenol | hydroxybenzene | 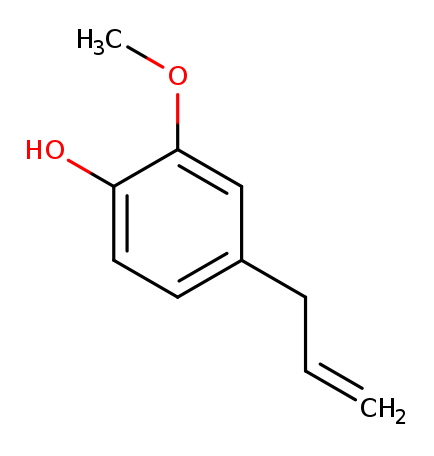 | 7LID, 7YWU, 7YWV, 3S0E |
| EQC | arctigenin | lignan | 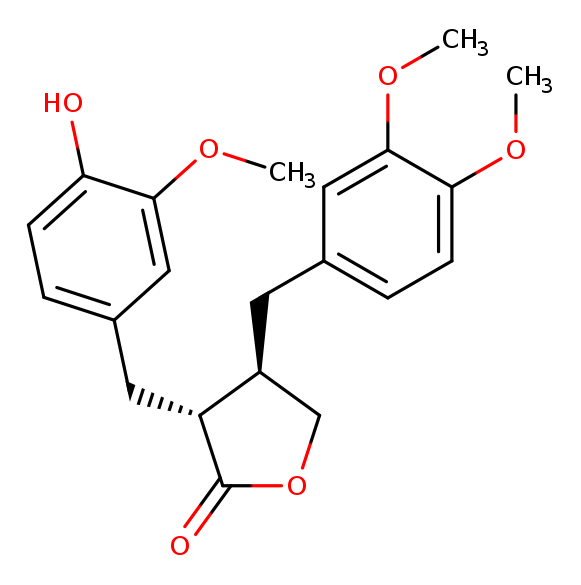 | 6LRM |
| ERD | eriodictyol | flavonoid | 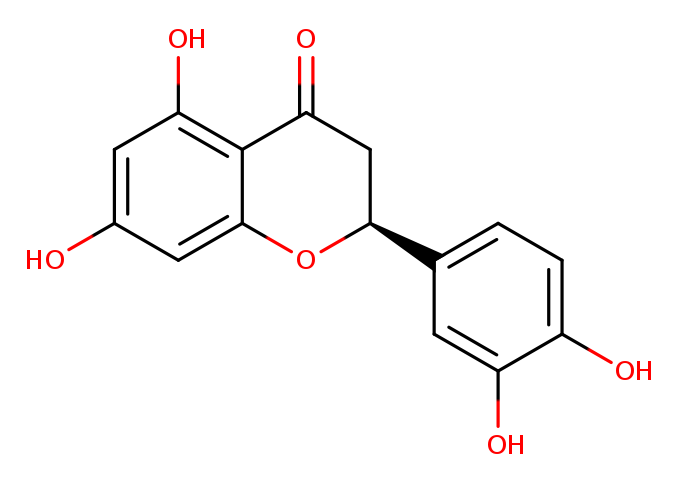 | 2NNL, 7YJB |
| EUG | isoeugenol | hydroxybenzene | 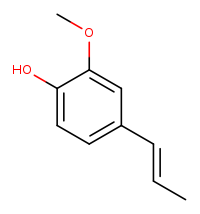 | 3REO, 1W1L, 2QU9, 3R5O, 1DZN, 1W1J, 1W1M, 2VAO, 1QLU, 1W1K |
| EZE | trans-oxyresveratrol | stilbene | 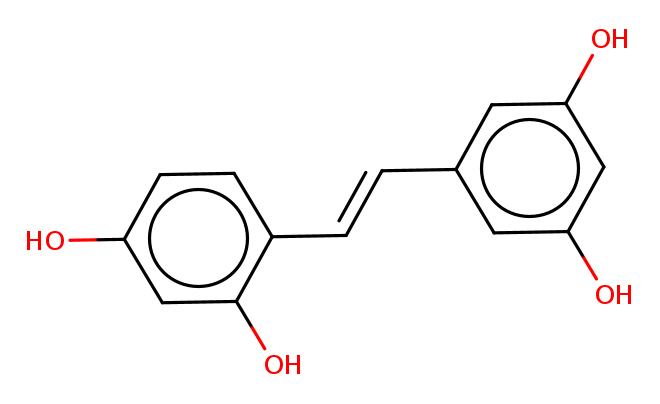 | 8IE5, 6GIC, 6SR9 |
| F94 | 7,3-dihydroxyflavone | flavonoid | 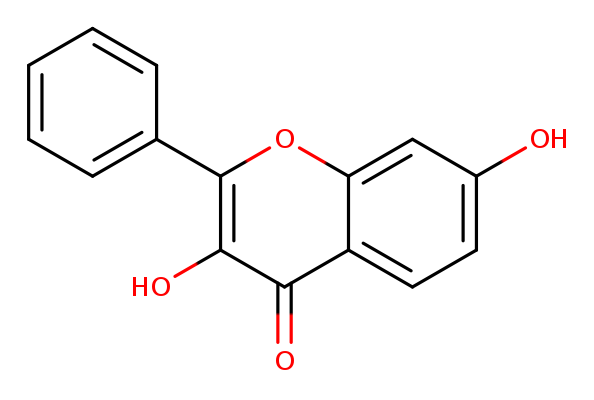 | 4HMH |
| FER | ferulic acid | hydroxycinnamic acid derivative | 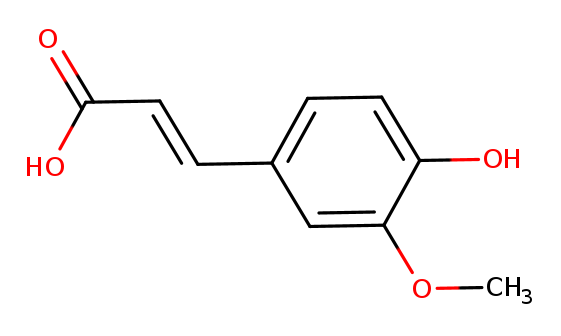 | 3NX2, 1GKL, 4PME, 6G21, 7ATJ, 3PFC, 2BNJ, 1JT2, 6YUX, 1GW2, 1GWT, 6ATJ, 7Z2U, 1KYZ, 7NTD, 1UWC, 3CBG, 2BJH, 5CXX, 4RGU, 4JB0, 2WTN, 6HOV, 6HOQ, 6HOP, 6MBY, 5X14 |
| FSE | fisetin | flavonoid | 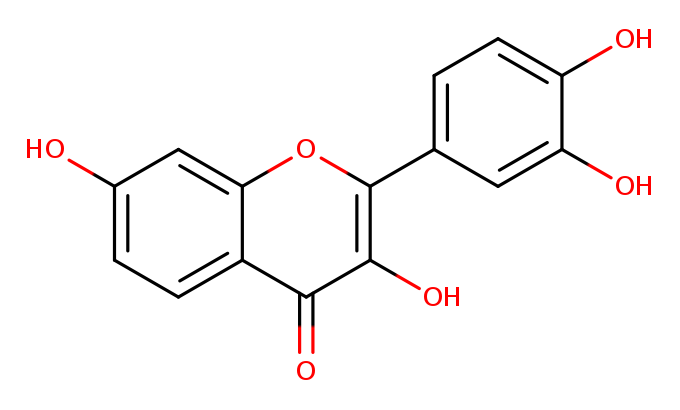 | 4RLT, 3P0H, 1XO2 |
| FX3 | 5-O-[(2E)-3-(4-hydroxy-3-methoxyphenyl)prop-2-enoyl]-alpha-L-ribofuranose | hydroxycinnamic acid derivative | 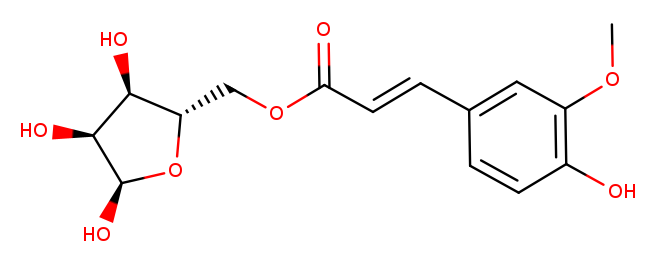 | 2VGD |
| G50 | phloretin | calchone | 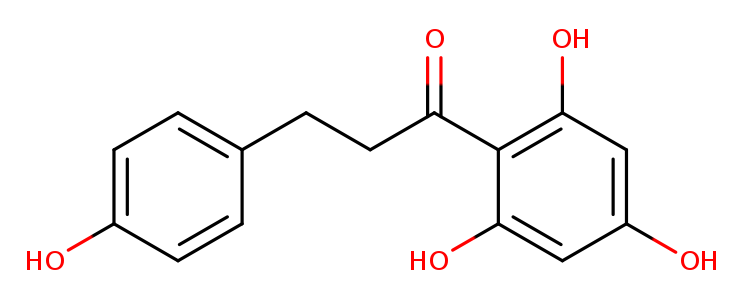 | 6L5S, 6L5R, 2UXI |
| G7G | genistein-7-O-glucuronide | flavonoid | 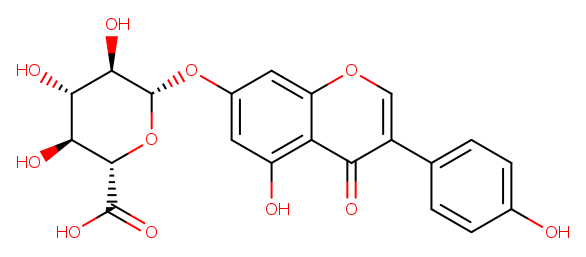 | 5AKV |
| GDE | gallic acid | phenolic acid | 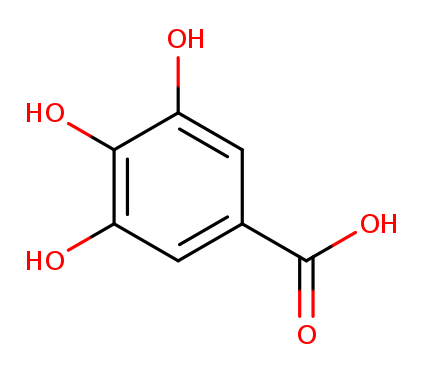 | 4IC0, 4J0H, 3WRB, 4Z5X, 3WKU, 3WPM, 3WR9, 3WR4, 3WR3, 7K4O |
| GEC | (+)-pinoresinol | lignan | 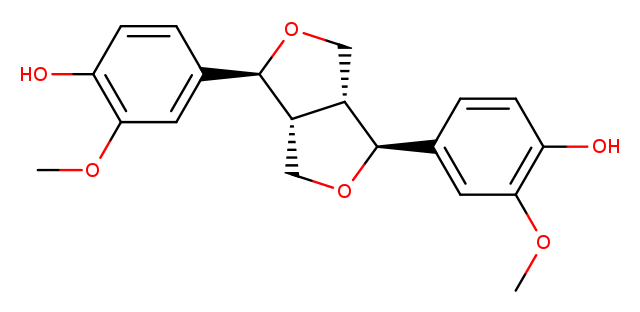 | 7CS4, 7CSB, 7CSH |
| GEF | (-)-pinoresinol | lignan | 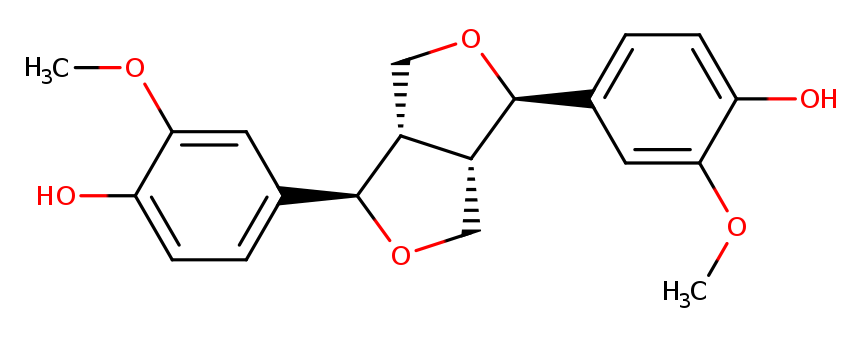 | 7CS5, 7CSC |
| GEN | genistein | flavonoid | 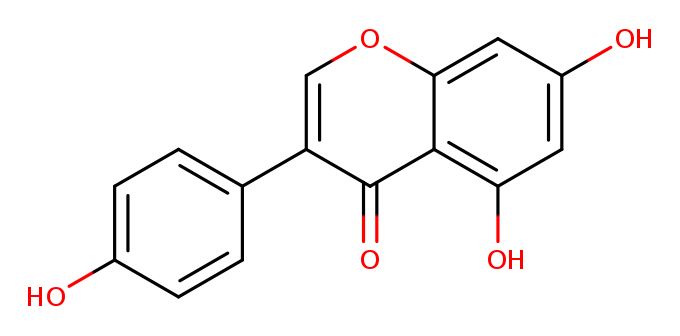 | 1X7J, 1X7R, 5AV4, 5AUZ, 8FOA, 3KGU, 3KGT, 2QA8, 1QKM, 6G8G, 4FJ1, 5KDA, 7NFB |
| GFR | (-)-lariciresinol | lignan | 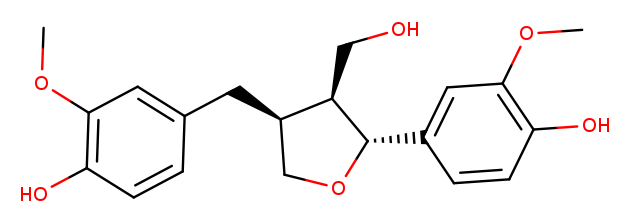 | 7CS6, 7CSE |
| GFU | (+)-lariciresinol | lignan | 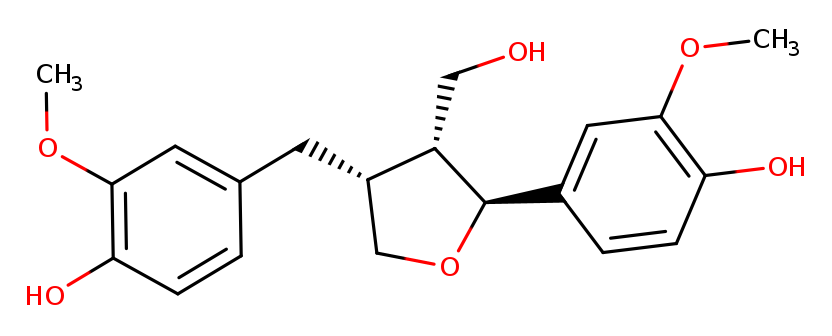 | 7CSD |
| GGE | (1S,2R)-1-(4-hydroxy-3-methoxyphenyl)-2-(2-methoxyphenoxy)propane-1,3-diol | other | 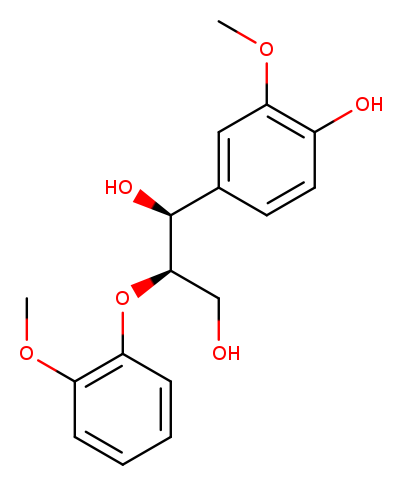 | 4YAI |
| GJK | feruloylmethane | hydroxycinnamic acid derivative | 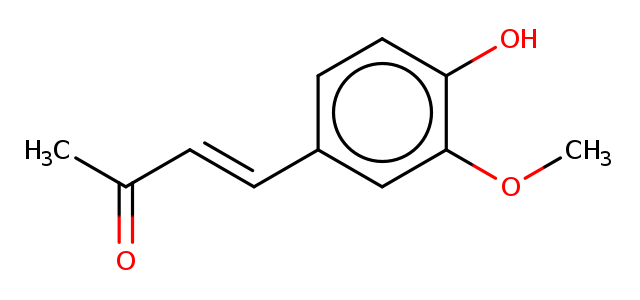 | 6HOR, 6HOP |
| GKP | chicoric acid | hydroxycinnamic acid derivative | 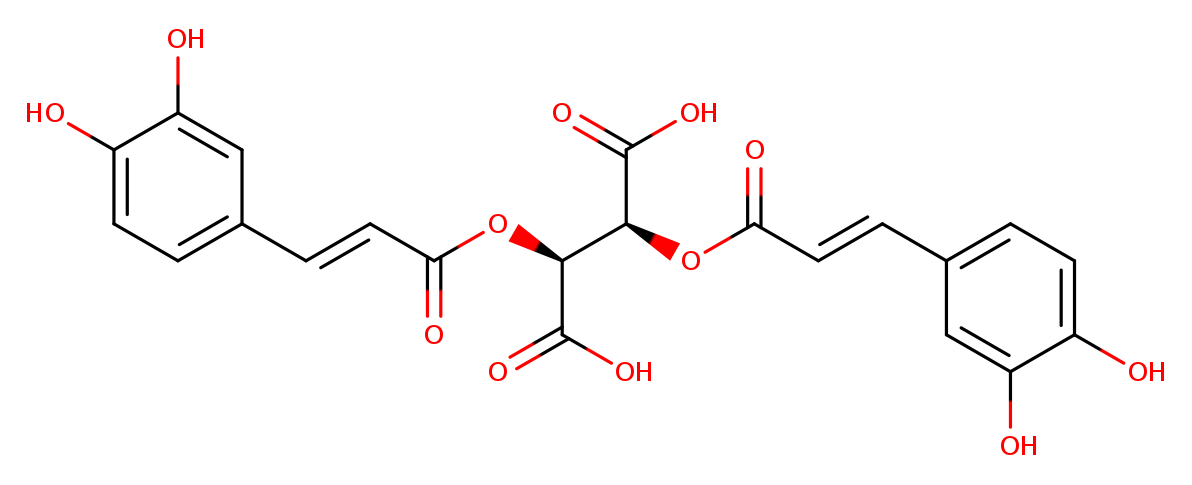 | 7UXZ, 6DIK |
| GO6 | (-)-secoisolariciresinol | lignan | 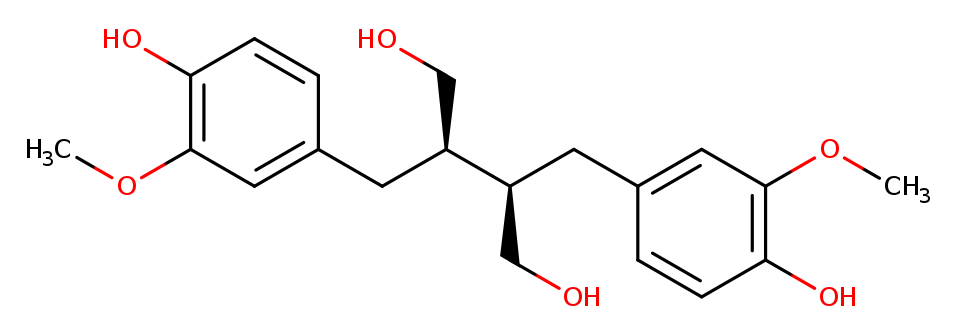 | 7CS8, 7CSF |
| GRE | 2,6-dihydroxybenzoic acid | phenolic acid |  | 4E3F, 2DVU |
| GTQ | 2,5-dihydroxybenzoic acid | phenolic acid |  | 3NW4, 3JUT, 4FAG, 7BPC, 4E3D, 3NL1 |
| H5A | trimethylgallic acid | phenolic acid |  | 5QG2, 5QGL |
| H6N | protocatechualdehyde | hydroxybenzene |  | 6I73 |
| H7Y | isoeugenol | hydroxybenzene |  | 7PBI, 5FXD |
| H9R | homoorientin | flavonoid |  | 7DNN |
| HC4 | p-coumaric acid | hydroxycinnamic acid derivative |  | 3PHY, 5J0A, 2O7B, 2O7F, 2J3J, 1GSX, 1GSW, 1GSV, 4I3J, 5MOV, 3UME, 3UMD, 6UN2, 3VE4, 3VE3, 1TS6, 1TS7, 1TS0, 1TS8, 1F98, 1F9I, 2QJ5, 2QJ7, 1OTD, 1OTE, 1OTB, 8DZX, 8DZY, 8DZU, 5GX9, 1T18, 1T19, 1T1A, 1T1B, 1T1C, 4F8J, 1S4R, 1S4S, 2PYP, 6MMD, 4EYO, 2I9V, 3UBA, 6UMY, 6VZK, 5VFM, 5Y8T, 1XFQ, 1XFN, 1NWZ, 4I3A, 2PHY, 2KX6, 8E1L, 4WL9, 1OTI, 3A5R, 3PYP, 1S1Z, 1S1Y, 1OTA, 1OT6, 1OT9, 2D01, 4QEM, 8BHH, 4HY8, 1UWN, 1D7E, 8E03, 8E02, 8E09, 1ODV, 1UWP, 6MKT, 4BBT, 4BBU, 4BBV, 1UGU, 4RGR, 6TC2, 7NR2, 8E1K, 2D02, 7SJJ, 2PYR, 4WLA, 4B9O, 2QWS, 7NSW, 7SPV, 7SPW, 7SPX, 4I38, 4I39, 4I3I, 1MZU, 4ALB, 2ZOI, 2ZOH, 5X13 |
| HCC | isoliquiritigenin | calchone |  | 5YX4, 1FP1, 4RLU, 6AJX, 6AJV |
| HCI | hydrocinnamic acid | hydroxycinnamic acid derivative |  | 1TOG, 4GM7, 1BXG, 6ZGS, 1V2F, 4E67, 1TOJ, 1TOI, 1AY8, 4USA, 5LTM, 5VFN, 3AYI, 7NTR, 7XRO, 7NRA, 1AHX |
| HER | europetin | flavonoid |  | 7DPU |
| HF6 | methyl 4-hydroxycinnamate | hydroxycinnamic acid derivative |  | 7DPS |
| HFC | esculetin | coumarin |  | 7DQI |
| HHF | 3,7-dihydroxyflavone | flavonoid |  | 4FJ0 |
| HMO | isoformononetin | flavonoid |  | 1FP2 |
| HQE | hydroquinone | hydroxybenzene |  | 4QOP, 3ZOF, 3ZOD, 5I3B, 7NNX, 7NMP, 7NIY, 5FSE, 4E3H, 8A18, 5I3A |
| HQN | hydroxyquinol | hydroxybenzene |  | 3O6J |
| HUL | hispidulin | flavonoid |  | 4XH6 |
| HW2 | isoquercetin | flavonoid |  | 6QCE |
| HWB | cyanidin | flavonoid |  | 6QCH |
| HWO | (2R)-7-[(2R,3R,4R,5S,6S)-6-(hydroxymethyl)-3-[(2R,3R,4R,5R,6S)-6-methyl-3,4,5-tris(oxidanyl)oxan-2-yl]oxy-4,5-bis(oxidanyl)oxan-2-yl]oxy-2-(4-methoxy-3-oxidanyl-phenyl)-5-oxidanyl-2,3-dihydrochromen-4-one | flavonoid |  | 7E45 |
| IJP | syringic acid | phenolic acid |  | 7Y8B |
| IPB | thymol | hydroxybenzene |  | 7T9C, 8BXV, 1E06, 7SJF |
| IRH | isorhamnetin | flavonoid |  | 7CBA, 6M8C |
| J6O | hyperoside | flavonoid |  | 7Z60 |
| J8D | diosmetin | flavonoid |  | 6M8D |
| J8G | rhamnetin | flavonoid |  | 6M8E |
| JUG | juglone | naphthoquinone |  | 6BV6, 6BV5, 6BV8, 6M0P, 3B7J, 6BV9 |
| JUV | 4-coumaroyl-(R)-3-(4-hydroxyphenyl)lactate | hydroxycinnamic acid derivative |  | 6MK2 |
| JXY | farrerol | flavonoid |  | 7YV4 |
| JZ3 | guaiacol | hydroxybenzene |  | 6HQK, 6HQM, 6HQL, 6HQO, 6HQN, 6HQP, 4QOQ, 4A6Z, 4A78, 6KRF, 5NCB, 4G05, 6YCJ, 6YCH, 6YCI, 3HT9 |
| KDH | epigallocatechin gallate | flavonoid |  | 3OOB, 2KDH, 4AWM, 3NG5, 7UPG, 6S2G |
| KKN | karrikin | other |  | 4JYM, 5DNU |
| KMC | galangin | flavonoid |  | 8A0O |
| KML | pinocembrin | flavonoid |  | 8A0R, 7YJ8, 7YJA |
| KMP | kaempherol | flavonoid |  | 5AV2, 4DET, 5AUX, 6M8B, 5AV3, 4REL, 3QWH, 1H1M, 7VEJ, 2C1Z |
| KXN | cianidanol | flavonoid |  | 3I52, 4C94, 4C9I |
| LU2 | luteolin | flavonoid |  | 4HKN, 4QYA, 6YA5, 4DGN, 5AUU, 6M8A, 4DEW, 3SZ1, 7D3B, 7EBV, 5II2, 5NDF, 4QXV |
| M5O | (-)-catechin | flavonoid |  | 8AGQ |
| MAX | matairesinol | lignan |  | 2BGM |
| MBD | 3-methylcatechol | hydroxybenzene |  | 3HJQ, 1KNF, 2WL9 |
| MCT | 4-methylcatechol | hydroxybenzene |  | 2EHZ, 3HJS, 3HPY, 1L4G, 3FW5, 1DMH, 4K7N, 5ZNH |
| MRI | morin | flavonoid |  | 5AUY, 8A0P, 7E4B, 6AE3, 6TT8 |
| MYC | myricetin | flavonoid |  | 3HBF, 7DPP, 2IOD, 5YUN, 6M88, 5HXC, 7B3E, 1E90, 4GQR, 7ELW, 5XI1, 6TTC, 3C1T, 2O63, 7E49 |
| MYF | tricetin | flavonoid |  | 2O65 |
| MYU | quercetagetin | flavonoid |  | 3V3V, 2O64 |
| NAR | naringenin | flavonoid |  | 4A87, 2UXU, 4DEU, 7BUR, 4D06, 1CGK, 2BRT, 6MS8, 1EYQ, 7VF0, 4EH3, 6G8H, 7VEZ, 5WKS, 5WKR, 7YJ9, 7YJ7 |
| NRA | norathyriol | other |  | 3SA0 |
| O9Q | 2-(3-methylphenyl)-5,7-bis(oxidanyl)chromen-4-one | flavonoid |  | 6Y55 |
| O9T | 2-(4-methylphenyl)-5,7-bis(oxidanyl)chromen-4-one | flavonoid |  | 6Y5C |
| OHP | 2-hydroxyphenylacetic acid | hydroxybenzene |  | 2INZ, 5RTW |
| P5G | anhydrosecoisolariciresinol | lignan |  | 6PYB |
| P5M | petunidin | flavonoid |  | 4REN |
| P5O | isorhapontigenin | stilbene |  | 8IE8 |
| PHB | 4-hydroxybenzoic acid | phenolic acid |  | 2BUW, 2BUR, 8AGA, 1G81, 1BKW, 6KCL, 6KCM, 6KCK, 1IUW, 1IUV, 1IUX, 1PXB, 1PDH, 3ZYB, 1G1B, 1CJ2, 1Q4S, 5D2A, 4LKE, 4LKD, 4LKF, 4OD5, 1CC4, 1PBE, 1BF3, 4EVS, 4EVQ, 4EY3, 3TYB, 1BGJ, 1BGN, 1DOB, 1DOC, 5IGA, 2PHH, 1PXA, 7CUO, 1K0I, 4F06, 3PCC, 5I5P, 1CJ3, 7DQB, 1TT8, 6AL9, 1JD3, 1PXC, 1CC6, 1YKJ, 3HGR, 3HGS, 1D7L, 5RTJ, 4E3G, 1CJ4, 3R3A, 3R36, 1FW9, 7ON9, 5W1E, 5M21 |
| PIT | piceatannol | stilbene |  | 7CCV, 5U97, 2JJ1, 4HD8 |
| PWH | caffeic acid 1,1-dimethylallyl ester | hydroxycinnamic acid derivative |  | 4PWH |
| PYG | pyrogallol | hydroxybenzene |  | 4QOM, 3HHX, 3O6R, 4V4D |
| QCT | quercitrin | flavonoid |  | 8DSE, 4GUE, 8DSH, 8EQ5, 5A4W |
| QPB | methyl (~{Z})-3-(4-hydroxyphenyl)-2-methyl-prop-2-enoate | hydroxycinnamic acid derivative |  | 6ZSW |
| QSO | biochanin A | flavonoid |  | 2QYO, 5JMM, 4FJ2 |
| QUE | quercetin | flavonoid |  | 3BPT, 2HCK, 3LJ0, 4LMU, 5FLI, 5FLJ, 8GQT, 3CF8, 4DFU, 4MRA, 4WNJ, 2O3P, 2MS6, 5AUW, 3BXX, 6M89, 3LM5, 1GP6, 6AWS, 6QCN, 6QCD, 7VUM, 6B1D, 2N6C, 1E8W, 2C9Z, 5XG4, 5A4V, 2UXH, 1H1I, 2JJ2, 6TTA, 6IJD, 3NVY |
| R0U | tectorigenin | flavonoid |  | 8ITA |
| R3S | resveratrol-3-O-sulfate | stilbene |  | 5AL0 |
| R3X | resveratrol-3-O-glucuronide | stilbene |  | 5AKS |
| R4G | resveratrol-4-O-glucuronide | stilbene |  | 5AKT |
| RCO | resorcinol | hydroxybenzene |  | 2OLY, 2OLZ, 3ZU1, 4QOO, 4DM3, 4E49, 2OMH, 2OMI, 2OM0, 2OM1, 6GSG, 3AQT, 1EVR, 1QIZ, 4AJX, 4Z10, 2W44 |
| RE2 | dihydroresveratrol | stilbene |  | 3FTX, 3FTU |
| REF | ellagic acid | other |  | 4YUA, 2ZJW, 6RWD |
| RMN | (R)-mandelic acid | other |  | 1MDL, 3ZGJ, 1MCZ, 5ZZX, 4P56 |
| ROA | rosmarinic acid | hydroxycinnamic acid derivative |  | 7BM3, 3QNL, 4PWI, 6MQD |
| RUT | rutin | flavonoid |  | 1RY8 |
| S5V | carvacrol | hydroxybenzene |  | 8BXW |
| SAK | sakuranetin | flavonoid |  | 3D04 |
| SAL | salicylic acid | phenolic acid |  | 3BPX, 3UNI, 3UNC, 3UNA, 3N8Y, 6WPG, 3REM, 3RET, 5EVY, 7LH2, 6L4M, 5X80, 3DEU, 1FO4, 7S9E, 7S9A, 7V75, 4HVR, 2I30, 5M78, 5SWR, 3ZMD, 3NJZ, 6EMM, 2Y7K, 2I2Z, 5F1A, 7EN1, 3B9M, 6UX1, 4EM2, 4EM0, 3KP6, 5S96, 4L39, 6TM4, 1WYG, 3W1W, 1PTH, 1FIQ, 2FN1, 4EQ4, 4EQL, 5U6M, 5U6N, 5OF1, 7EMY, 5WM2, 3TWP, 5YJS, 3AX7, 3AX9, 1JGS, 3GF2, 2Y7P, 2Y7W, 7BNI, 3HGX, 1M6E, 2E1Q, 6ZA5, 7KJL, 1Y7I, 6JQX, 3NVC, 4Y03, 6YMO, 6S37 |
| SL0 | 5,7-dihydroxy-2-(4-hydroxyphenyl)-4-oxo-4H-chromen-3-yl 3,4-di-O-acetyl-6-deoxy-alpha-L-mannopyranoside | flavonoid |  | 3UBD |
| SMN | (S)-mandelic acid | other |  | 1MDL, 3TTE, 6A0V, 5ZZR, 4P56 |
| SN1 | 2,4-Dihydroxy-Trans Cinnamic Acid | hydroxycinnamic acid derivative |  | 1K2I |
| SQH | 3,4,7,8-Tetrahydroxyflavone | flavonoid |  | 7C2Z, 7C6P |
| STL | resveratrol | stilbene |  | 6JEM, 7WAQ, 4QOH, 4QOJ, 5NZL, 8AKA, 1U0W, 3MNQ, 7K1C, 2L98, 8ABT, 4DPN, 2YDX, 5CR1, 7Q9O, 3CKL, 1Z1F, 1SG0, 4PP6, 1CGZ, 5J54, 7CCU, 7KD8, 4JAZ, 1DVS, 7CCW, 3FTS, 5U90, 5BTR, 4QER, 2JIZ, 7VB8, 4HDA, 4Q93 |
| SXX | sinapic acid | hydroxycinnamic acid derivative |  | 1WB4, 4Q8B |
| SZQ | trans-methylferulate | hydroxycinnamic acid derivative |  | 7B6B |
| TCA | cinnamic acid | hydroxycinnamic acid derivative |  | 4CQ5, 1BE8, 7AZ8, 1BE6, 2O78, 6R2P, 2XAJ, 3NZ4 |
| TO2 | (1R,2S)-1-(3,4-dimethoxyphenyl)-2-(2-methoxyphenoxy)propane-1,3-diol | other |  | 3TA4 |
| TWO | veratric acid | phenolic acid |  | 4EGN |
| USE | orientin | flavonoid |  | 7NUG, 7NUH |
| V33 | 2-(3-methoxyphenyl)ethanoic acid | hydroxybenzene |  | 5ST8 |
| V55 | p-vanillin | hydroxybenzene |  | 2VSU, 2VSS, 5OMR, 6XM9, 7R0S, 7R0U, 7XWV, 5J55, 4RGS, 6HOU, 6HOP, 6YCT, 6YCK, 6YCL, 6YCM, 5FXP |
| V5P | lignostilbene | stilbene |  | 6XM8 |
| VK3 | menadione | naphthoquinone |  | 6RVH, 8DQV, 4F8Y, 7UUR, 2QR2, 1TUV |
| VKK | p-coumaroyl glucose | hydroxycinnamic acid derivative |  | 7ONF |
| VNL | vanillate | phenolic acid |  | 2Y2X, 3SYS, 4EYG, 5Z7B, 1XLR, 6LG2, 4L6D, 2AHC, 5X1J |
| X8W | naringenin chalchone | chalchone |  | 4D06 |
| XEG | epigallo catechin gallate | flavonoid |  | 6QCJ, 6DHL |
| YRL | p-tyrosol | hydroxybenzene |  | 7OFS, 4P6T |
| YTX | homovanillic acid | hydroxybenzene |  | 5RSX, 5MOH |
| ZF1 | daidzein | flavonoid |  | 7EBU |
| ZYC | ethyl ferulate | hydroxycinnamic acid derivative |  | 3PFB, 3QM1, 7XRI |

**Table** **S2.** Atom typing used in this work. We only include atom types present in pairs observed at least 1000-times within the database, with the exception of the metal M type, which is observer 550 times.

| **Protein/cofactor/ions/water atom type** | **Description** | **Ligand atom type** | **Description** |
| --- | --- | --- | --- |
| C.3_bk | carbon sp^3^ on protein backbone | C3 | carbon sp^3^ on ligand |
| C.3_sc | carbon sp^3^ on protein sidechain | C2 | carbon sp^2^ on ligand |
| C.3_cf | carbon sp^3^ on a cofactor | C.ar | carbon on ligand aromatic ring |
| C.2_bk | carbon sp^2^ on protein backbone | O.3 | oxygen sp^3^ on ligand |
| C.2_sc | carbon sp^2^ on protein sidechain | O.2 | oxygen sp^2^ on ligand |
| C.2_cf | carbon sp^2^ on a cofactor | O.co2 | Oxygen in ligand in carboxylate |
| C.ar_sc | carbon in aromatic rings on protein sidechain | / | |
| C.ar_cf | carbon in aromatic rings on a cofactor |  |  |
| C.cat_sc | carbon in amidinium and guanidinium protein sidechains |  |  |
| O.3_sc | oxygen sp^3^ on protein sidechain |  |  |
| O.3_cf | oxygen sp^3^ on a cofactor |  |  |
| O.3_w | water oxygen |  |  |
| O.co2_sc | oxygen in protein sidechain in carboxylate |  |  |
| N.am_bk | nitrogen in amide in protein backbone |  |  |
| N.am_sc | nitrogen in amide in protein sidechain |  |  |
| N.3_sc | nitrogen sp^3^ on protein sidechain |  |  |
| N.2_sc | nitrogen sp^2^ on protein sidechain |  |  |
| N.pl3_sc | nitrogen on protein sidechain in amidinimum and guanidinium groups |  |  |
| N.pl3_cf | nitrogen on a cofactor in amidinimum and guanidinium groups |  |  |
| S.3_sc | sulfur sp^3^ on protein sidechain |  |  |
| M | Metal ion (Cu, Fe, Mg, Ni, Mn, K, Na, Zn, Ca) |  |  |

**Table S3.** Number of atom pairs derived from the protein-polyphenol complexes.

| **Protein atom type** | **Ligand atom type** | **Pair count** | **Protein atom type** | **Ligand atom type** | **Pair count** |
| --- | --- | --- | --- | --- | --- |
| C.3_sc | C.ar | 215856 | C.cat_sc | O.2 | 319 |
| C.ar_sc | C.ar | 118637 | C.ar_cf | O.2 | 292 |
| C.3_bk | C.ar | 73855 | O.2_cf | C.2 | 273 |
| N.am_bk | C.ar | 71050 | N.3_sc | O.co2 | 263 |
| C.2_bk | C.ar | 68976 | N.ar_cf | C.2 | 258 |
| O.2_bk | C.ar | 66058 | N.3_sc | O.2 | 234 |
| C.3_sc | O.3 | 62559 | N.ar_cf | C.3 | 233 |
| O.3_w | C.ar | 41757 | M | C.3 | 232 |
| C.3_sc | C.2 | 34114 | N.pl3_cf | O.3 | 226 |
| C.ar_sc | O.3 | 32626 | O.3_cf | C.3 | 225 |
| C.3_sc | C.3 | 27239 | N.2_cf | O.3 | 220 |
| C.2_sc | C.ar | 24614 | O.3_cf | O.2 | 207 |
| C.ar_sc | C.2 | 23032 | M | C.2 | 201 |
| C.3_bk | O.3 | 22815 | P.3_cf | C.ar | 197 |
| N.am_bk | O.3 | 22092 | O.3_cf | C.2 | 191 |
| C.2_bk | O.3 | 21379 | S.3_cf | C.ar | 191 |
| O.2_bk | O.3 | 21210 | O.2_cf | C.3 | 181 |
| O.3_sc | C.ar | 20881 | Cl_cf | C.ar | 178 |
| C.3_sc | O.co2 | 17737 | O.3_sc | C.1 | 177 |
| C.3_sc | O.2 | 17480 | O.2_cf | O.2 | 176 |
| C.ar_sc | C.3 | 15333 | O.2_cf | O.co2 | 161 |
| O.3_w | O.3 | 14339 | N.ar_cf | O.2 | 149 |
| O.co2_sc | C.ar | 13600 | N.2_cf | C.2 | 141 |
| C.3_bk | C.2 | 12814 | N.ar_cf | O.co2 | 136 |
| N.am_bk | C.2 | 12591 | O.co2_cf | O.3 | 134 |
| N.pl3_sc | C.ar | 12422 | N.pl3_cf | C.2 | 132 |
| O.2_bk | C.2 | 12172 | N.am_cf | O.3 | 118 |
| C.2_bk | C.2 | 12117 | N.4_sc | O.3 | 117 |
| C.ar_sc | O.2 | 9725 | N.2_cf | C.3 | 111 |
| C.3_bk | C.3 | 9270 | M | O.2 | 105 |
| N.am_bk | C.3 | 8904 | N.3_bk | C.2 | 104 |
| C.ar_sc | O.co2 | 8780 | N.2_cf | O.co2 | 104 |
| C.2_bk | C.3 | 8470 | M | O.co2 | 102 |
| O.2_bk | C.3 | 8421 | O.co2_cf | C.3 | 98 |
| C.2_sc | O.3 | 8216 | O.3_w | C.1 | 94 |
| N.ar_sc | C.ar | 7459 | P.3_cf | O.3 | 93 |
| S.3_sc | C.ar | 7004 | N.pl3_cf | C.3 | 87 |
| O.3_w | C.2 | 6969 | N.3_bk | O.3 | 87 |
| C.2_cf | C.ar | 6798 | N.pl3_cf | O.2 | 87 |
| C.3_bk | O.co2 | 6728 | O.3_cf | O.co2 | 85 |
| N.am_bk | O.co2 | 6505 | C.3_cf | C.1 | 81 |
| C.3_bk | O.2 | 6457 | N.3_cf | C.ar | 80 |
| N.am_bk | O.2 | 6395 | N.am_cf | C.2 | 77 |
| O.2_bk | O.2 | 6386 | N.pl3_cf | O.co2 | 75 |
| O.3_w | C.3 | 6325 | N.3_bk | C.3 | 72 |
| C.2_bk | O.co2 | 6294 | S.3_cf | O.3 | 71 |
| C.2_bk | O.2 | 6222 | N.am_cf | O.2 | 67 |
| O.2_sc | C.ar | 6171 | Cl_cf | O.3 | 64 |
| O.3_sc | O.3 | 6001 | S.3_cf | C.3 | 60 |
| O.2_bk | O.co2 | 5988 | N.3_bk | O.2 | 55 |
| N.am_sc | C.ar | 5887 | O.co2_cf | O.2 | 54 |
| C.3_cf | C.ar | 5428 | N.am_cf | C.3 | 53 |
| N.2_sc | C.ar | 5139 | N.2_cf | O.2 | 52 |
| O.co2_sc | O.3 | 4979 | O.co2_cf | C.2 | 52 |
| C.ar_cf | C.ar | 4694 | N.4_sc | C.3 | 47 |
| O.3_w | O.co2 | 4483 | S.3_sc | C.1 | 47 |
| C.2_sc | C.2 | 3834 | N.pl3_sc | C.1 | 46 |
| O.3_sc | C.2 | 3787 | C.2_sc | C.1 | 45 |
| C.cat_sc | C.ar | 3745 | C.3_sc | S.3 | 40 |
| O.3_w | O.2 | 3693 | O.2_w | C.ar | 39 |
| C.2_sc | C.3 | 3454 | N.3_bk | O.co2 | 38 |
| N.pl3_sc | O.3 | 3423 | P.3_cf | C.3 | 36 |
| N.pl3_sc | C.2 | 3392 | N.4_sc | O.2 | 36 |
| N.3_sc | C.ar | 3051 | N.4_sc | C.2 | 35 |
| N.ar_sc | O.3 | 2749 | N.2_bk | C.ar | 34 |
| O.3_sc | C.3 | 2713 | N.am_cf | O.co2 | 34 |
| O.co2_sc | C.2 | 2418 | O.co2_bk | C.ar | 27 |
| N.pl3_sc | O.co2 | 2414 | O.3_cf | C.1 | 27 |
| C.2_sc | O.co2 | 2364 | N.3_cf | O.3 | 27 |
| O.2_sc | O.3 | 2227 | P.3_cf | O.2 | 22 |
| O.3_sc | O.co2 | 2122 | N.am_sc | C.1 | 22 |
| N.am_sc | O.3 | 1987 | O.co2_bk | O.3 | 22 |
| N.ar_cf | C.ar | 1951 | S.3_cf | C.2 | 22 |
| M | C.ar | 1918 | N.2_bk | O.3 | 21 |
| S.3_sc | O.3 | 1853 | Cl_cf | C.3 | 19 |
| O.co2_sc | C.3 | 1850 | N.2_sc | C.1 | 18 |
| O.2_cf | C.ar | 1825 | O.2_w | C.2 | 18 |
| O.3_cf | C.ar | 1710 | N.3_bk | C.1 | 18 |
| N.2_sc | O.3 | 1652 | O.co2_bk | C.3 | 17 |
| C.2_sc | O.2 | 1645 | S.3_cf | O.2 | 17 |
| C.2_cf | O.3 | 1620 | O.2_w | C.3 | 17 |
| S.3_sc | C.2 | 1553 | N.4_sc | O.co2 | 16 |
| N.pl3_sc | C.3 | 1515 | P.3_cf | C.2 | 16 |
| O.3_sc | O.2 | 1383 | C.cat_sc | C.1 | 15 |
| C.3_cf | O.3 | 1373 | O.3_w | S.3 | 14 |
| C.ar_cf | O.3 | 1255 | O.3_bk | C.3 | 14 |
| O.co2_sc | O.2 | 1190 | O.co2_sc | C.1 | 13 |
| C.3_sc | C.1 | 1149 | O.2_sc | C.1 | 13 |
| C.cat_sc | C.2 | 1111 | Cl_cf | C.2 | 13 |
| N.pl3_sc | O.2 | 1104 | N.am_bk | S.3 | 12 |
| C.cat_sc | O.3 | 1077 | S.3_cf | O.co2 | 12 |
| N.3_sc | O.3 | 1072 | O.2_w | O.3 | 12 |
| C.2_cf | C.3 | 1063 | S.2_cf | C.3 | 12 |
| O.co2_sc | O.co2 | 1061 | Cl_cf | O.2 | 11 |
| N.am_sc | C.2 | 991 | N.3_cf | O.2 | 11 |
| O.2_sc | C.2 | 972 | O.3_bk | C.1 | 9 |
| S.3_sc | C.3 | 971 | O.co2_bk | C.2 | 8 |
| N.2_cf | C.ar | 957 | C.3_bk | S.3 | 8 |
| N.ar_sc | C.2 | 941 | C.2_bk | S.3 | 8 |
| N.pl3_cf | C.ar | 908 | O.2_bk | S.3 | 8 |
| N.2_sc | C.2 | 894 | N.ar_sc | C.1 | 8 |
| C.3_cf | C.3 | 878 | Se_cf | C.ar | 8 |
| O.2_sc | C.3 | 863 | P.3_cf | O.co2 | 6 |
| C.2_cf | C.2 | 836 | Se_cf | C.2 | 6 |
| N.ar_sc | C.3 | 811 | O.2_w | O.2 | 5 |
| N.am_sc | C.3 | 804 | N.ar_cf | C.1 | 4 |
| C.cat_sc | O.co2 | 767 | C.ar_cf | C.1 | 4 |
| N.2_sc | C.3 | 763 | N.4_sc | S.3 | 4 |
| C.3_cf | C.2 | 758 | O.co2_sc | S.3 | 4 |
| S.3_sc | O.2 | 714 | Cl_cf | O.co2 | 4 |
| C.ar_cf | C.3 | 676 | S.2_cf | O.3 | 4 |
| O.2_sc | O.co2 | 620 | O.2_cf | C.1 | 3 |
| N.am_sc | O.co2 | 602 | O.co2_bk | O.2 | 3 |
| C.ar_cf | C.2 | 579 | O.co2_bk | O.co2 | 3 |
| M | O.3 | 575 | O.3_sc | S.3 | 3 |
| N.ar_cf | O.3 | 563 | N.3_cf | C.3 | 3 |
| N.2_sc | O.co2 | 550 | S.O_sc | C.2 | 3 |
| O.3_cf | O.3 | 538 | O.3_bk | O.3 | 3 |
| O.co2_cf | C.ar | 532 | P.3_cf | C.1 | 2 |
| N.am_cf | C.ar | 511 | O.co2_bk | C.1 | 2 |
| N.ar_sc | O.2 | 475 | N.4_bk | O.3 | 2 |
| O.2_cf | O.3 | 474 | N.3_cf | C.2 | 2 |
| C.cat_sc | C.3 | 471 | S.3_cf | C.1 | 2 |
| C.3_cf | O.2 | 470 | N.2_bk | O.2 | 2 |
| N.ar_sc | O.co2 | 465 | Se_cf | O.2 | 2 |
| C.2_cf | O.2 | 464 | O.co2_w | O.3 | 2 |
| N.3_sc | C.3 | 437 | S.O_sc | C.ar | 2 |
| N.am_sc | O.2 | 430 | S.O_sc | O.co2 | 2 |
| C.2_cf | O.co2 | 425 | O.co2_cf | O.co2 | 2 |
| O.2_sc | O.2 | 423 | O.3_bk | C.ar | 2 |
| C.3_bk | C.1 | 412 | N.3_sc | C.1 | 1 |
| N.2_sc | O.2 | 378 | S.3_sc | S.3 | 1 |
| N.4_sc | C.ar | 377 | M | C.1 | 1 |
| C.2_bk | C.1 | 376 | O.co2_w | C.ar | 1 |
| O.2_bk | C.1 | 375 | O.co2_w | C.3 | 1 |
| N.am_bk | C.1 | 374 | N.4_bk | C.ar | 1 |
| N.3_sc | C.2 | 369 | S.O_sc | C.3 | 1 |
| N.3_bk | C.ar | 367 | O.2_w | O.co2 | 1 |
| C.ar_sc | C.1 | 351 | N.3_cf | O.co2 | 1 |
| C.ar_cf | O.co2 | 342 | O.3_bk | O.2 | 1 |
| C.3_cf | O.co2 | 335 | O.3_bk | C.2 | 1 |
| S.3_sc | O.co2 | 321 |  |  |  |

**Table S4.** Overview of the main properties of phenolic acid interactions.

| **Interaction type** | **Residues/metals involved** | **Average distance (Å)** | **Average angle (°)** | **Average offset (Å)** | **Geometry** |
| --- | --- | --- | --- | --- | --- |
| **Hydrophobic** | LEU: 231, ILE: 120, PHE: 118, TYR: 112, VAL: 105, TRP: 90, PRO: 82, ALA: 62, LYS: 33, THR: 32, ARG: 31, GLU: 16, GLN: 15, MET: 10, HIS: 7, ASP: 7, ASN: 3 | 3.7 ± 0.8 | N/A | N/A | N/A |
| **Direct hydrogen bond** | 'TYR': 214, 'SER': 131, 'THR': 96, 'ASN': 74, 'ALA': 66, 'GLN': 57, 'GLU': 53, 'GLY': 50, 'ARG': 47, 'LYS': 37, 'HIS': 30, 'LEU': 24, 'ASP': 18, 'MET': 14, 'VAL': 13, 'PRO': 9, 'TRP': 7, 'PHE': 7, 'CYS': 4, 'ILE': 1 | 3.1 ± 0.5  2.4 ± 0.6 | 141.4 ± 21.6 | N/A | N/A |
| **Water mediated H-bond** | ARG': 82, 'GLY': 23, 'THR': 19, 'LYS': 17, 'TYR': 16, 'ASP': 15, 'SER': 10, 'HIS': 9, 'ALA': 9, 'LEU': 8, 'ASN': 7, 'GLU': 6, 'MET': 6, 'GLN': 5, 'PHE': 5, 'VAL': 3, 'TRP': 2, 'PRO': 1 | HWO = 3.3 ± 0.5  3.2 ± 0.4 | 135.5 ± 22.4  95.5 ± 19.0 | N/A | N/A |
| **Salt bridge** | 'ARG': 141, 'LYS': 34 | 3.9 ± 0.7 | N/A | N/A | N/A |
| **π-π stacking** | PHE': 43, 'TYR': 21, 'HIS': 13, 'TRP': 12 | 4.7 ± 0.5 | T: 78.6 ± 8.1  P: 6.8 ± 3.4 | 1.1 ± 0.5 | 'T': 72  'P': 17 |
| **π-cation interaction** | LYS': 8, 'HIS': 4, 'ARG': 3 | 4.6 ± 0.6 | N/A | 1.2 ± 0.5 | N/A |
| **Metal complexation** | 'FE': 77, 'MN': 8, 'ZN': 5, 'MG': 3, 'NI': 1 | 2.1 ± 0.2 | N/A | N/A | 'trigonal.bipyramidal': 36, 'square.pyramidal': 22, 'linear': 16, 'tetrahedral': 10, 'octahedral': 8, 'square.planar': 2} |

**Table S5.** Overview of the main properties of flavonoid interactions.

| **Interaction type** | **Residues/metals involved** | **Average distance (Å)** | **Average angle (°)** | **Average offset (Å)** | **Geometry** |
| --- | --- | --- | --- | --- | --- |
| **Hydrophobic** | LEU': 332, 'ILE': 261, 'PHE': 216, 'TYR': 125, 'VAL': 117, 'LYS': 93, 'ALA': 79, 'THR': 53, 'TRP': 44, 'PRO': 43, 'GLU': 31, 'ARG': 18, 'GLN': 16, 'ASP': 15, 'MET': 11, 'ASN': 9, 'HIS': 8 | 3.7 ± 0.2 | N/A | N/A | N/A |
| **Direct hydrogen bond** | SER': 136, 'ASP': 126, 'GLU': 114, 'LYS': 106, 'ASN': 89, 'THR': 86, 'ARG': 81, 'VAL': 75, 'GLN': 63, 'TYR': 63, 'GLY': 50, 'HIS': 49, 'CYS': 28, 'PRO': 26, 'LEU': 22, 'PHE': 21, 'ALA': 21, 'ILE': 13, 'MET': 13, 'TRP': 2 | 3.3 ± 0.5  2.6 ± 0.6 | 134.8 ± 20.6 | N/A | N/A |
| **Water mediated H-bond** | THR': 45, 'SER': 39, 'ASP': 38, 'LYS': 34, 'TYR': 33, 'ARG': 31, 'GLU': 29, 'LEU': 22, 'ASN': 21, 'HIS': 21, 'GLN': 20, 'GLY': 19, 'VAL': 12, 'ALA': 10, 'ILE': 9, 'PHE': 4, 'PRO': 4, 'TRP': 4, 'MET': 2 | 3.3 ± 0.5  3.3 ± 0.5 | 134.9 ± 23.0  91.9 ± 16.0 | N/A | N/A |
| **Salt bridge** | ARG': 5 | 4.5 ± 0.6 | N/A | N/A | N/A |
| **π-π stacking** | 'PHE': 107, 'TYR': 59, 'HIS': 19, 'TRP': 13 | 4.3 ± 0.6 | T: 79.1 ± 8.5  P: 11.4 ± 6.1 | 1.2 ± 0.5 | 'T': 83  'P': 115 |
| **π-cation interaction** | 'LYS': 16, 'ARG': 13, 'HIS': 1 | 4.3 ± 0.6 | N/A | 1.3 ± 0.5 | N/A |
| **Metal complexation** | 'Mn': 19, 'Mg': 6, 'Ni': 4, 'Zn': 2, 'Fe': 1 | 2.2 ± 0.1 | N/A | N/A | 'octahedral': 23, 'square.pyramidal': 4, 'tetrahedral': 2, 'trigonal.bipyramidal': 2, 'square.planar': 1 |

**Table S6.** Overview of the main properties of cinnamic acid derivatives interactions.

| **Interaction type** | **Residues/metals involved** | **Average distance (Å)** | **Average angle (°)** | **Average offset (Å)** | **Geometry** |
| --- | --- | --- | --- | --- | --- |
| **Hydrophobic** | PHE': 457, 'VAL': 187, 'ALA': 173, 'LEU': 142, 'THR': 110, 'ILE': 84, 'TYR': 80, 'TRP': 30, 'PRO': 20, 'GLN': 17, 'LYS': 12, 'ARG': 9, 'GLU': 8, 'ASP': 7, 'ASN': 5, 'HIS': 4 | 3.6 ± 0.2 | N/A | N/A | N/A |
| **Direct hydrogen bond** | 'TYR': 210, 'GLU': 128, 'THR': 123, 'CYS': 123, 'SER': 77, 'ASN': 57, 'GLN': 53, 'ASP': 52, 'ARG': 49, 'LYS': 34, 'GLY': 25, 'HIS': 20, 'PHE': 18, 'VAL': 16, 'LEU': 14, 'ALA': 13, 'MET': 12, 'PRO': 6, 'TRP': 6, 'ILE': 4 | 3.1 ± 0.5  2.4 ± 0.6 | 141.9 ± 19.6 | N/A | N/A |
| **Water mediated H-bond** | 'ARG': 25, 'GLN': 20, 'ASN': 17, 'HIS': 15, 'SER': 14, 'TRP': 12, 'LYS': 12, 'THR': 10, 'LEU': 10, 'GLY': 8, 'GLU': 8, 'VAL': 8, 'ALA': 7, 'ASP': 6, 'MET': 2, 'TYR': 2, 'PHE': 2, 'ILE': 2, 'PRO': 1} | 3.2 ± 0.4  3.4 ± 0.5 | 135.2 ± 22.2  96.3 ± 18.7 | N/A | N/A |
| **Salt bridge** | 'ARG': 48, 'LYS': 42 | 3.9 ± 0.7 | N/A | N/A | N/A |
| **π-π stacking** | 'PHE': 82, 'HIS': 11, 'TRP': 11, 'TYR': 9} | 5.1 ± 0.2 | 76.8 ± 6.7  15.7 ± 2.6 | 1.4 ± 0.4 | T': 110  'P': 3 |
| **π-cation interaction** | 'HIS': 3, 'LYS': 3, 'ARG': 3 | 3.9 ± 0.4 | N/A | 1.0 ± 0.2 | N/A |
| **Metal complexation** | Mg: 2 | 2.4 ± 0.0 | N/A | N/A | square.pyramidal': 2 |

**Table S7**. Overview of the main properties of hydroxybenzene interactions.

| **Interaction type** | **Residues/metals involved** | **Average distance (Å)** | **Average angle (°)** | **Average offset (Å)** | **Geometry** |
| --- | --- | --- | --- | --- | --- |
| **Hydrophobic** | PHE': 191, 'ILE': 145, 'LEU': 134, 'TYR': 85, 'VAL': 78, 'TRP': 65, 'ALA': 43, 'PRO': 38, 'HIS': 33, 'ARG': 29, 'THR': 27, 'LYS': 14, 'ASN': 9, 'GLU': 6, 'GLN': 5, 'ASP': 2 | 3.7 ± 0.3 | N/A | N/A | N/A |
| **Direct hydrogen bond** | 'TYR': 65, 'ARG': 55, 'CYS': 44, 'GLY': 43, 'ALA': 33, 'ASN': 29, 'THR': 24, 'ASP': 24, 'HIS': 23, 'LYS': 22, 'GLN': 22, 'GLU': 21, 'VAL': 18, 'SER': 14, 'PHE': 7, 'TRP': 5, 'MET': 5, 'PRO': 2, 'LEU': 1 | 3.2 ± 0.5  2.4 ± 0.6 | 140.2 ± 21.2 | N/A | N/A |
| **Water mediated H-bond** | ALA': 24, 'ASN': 20, 'TYR': 16, 'HIS': 11, 'GLU': 10, 'ARG': 5, 'GLN': 5, 'SER': 4, 'TRP': 4, 'GLY': 4, 'THR': 3, 'ASP': 3, 'VAL': 3, 'PHE': 3, 'CYS': 2 | 3.2 ± 0.5  3.2 ± 0.5 | 140.6 ± 24.1  90.8 ± 13.8 | N/A | N/A |
| **Salt bridge** | ARG': 31 | 4.1 ± 0.7 | N/A | N/A | N/A |
| **π-π stacking** | 'PHE': 43, 'HIS': 28, 'TYR': 11, 'TRP': 6 | 4.3 ± 0.7 | 78.8 ± 7.1  9.2 ± 6.6 | 1.2 ± 0.5 | 'T': 43  'P': 45 |
| **π-cation interaction** | 'HIS': 3, 'ARG': 3 | 5.1 ± 0.5 | N/A | 1.4 ± 0.3 | N/A |
| **Metal complexation** | 'Fe': 84, 'Mg': 3, 'Mn': 1, 'Zn': 1 | 2.1 ± 0.2 | N/A | N/A | trigonal.bipyramidal': 38, 'octahedral': 25, 'square.pyramidal': 19, 'tetrahedral': 3 |

**Table S8.** Overview of the main properties of stilbene interactions.

| **Interaction type** | **Residues/metals involved** | **Average distance (Å)** | **Average angle (°)** | **Average offset (Å)** | **Geometry** |
| --- | --- | --- | --- | --- | --- |
| **Hydrophobic** | 'PHE': 114, 'LEU': 110, 'ILE': 59, 'ALA': 36, 'VAL': 25, 'LYS': 25, 'TRP': 21, 'THR': 18, 'PRO': 13, 'TYR': 11, 'ARG': 9, 'GLU': 7, 'GLN': 7, 'HIS': 6, 'ASN': 5, 'ASP': 2 | 3.7 ± 0.2 | N/A | N/A | N/A |
| **Direct hydrogen bond** | 'GLU': 36, 'SER': 34, 'VAL': 24, 'THR': 24, 'ASP': 20, 'LYS': 19, 'TYR': 14, 'ARG': 13, 'HIS': 13, 'ASN': 13, 'PHE': 11, 'LEU': 8, 'TRP': 6, 'GLN': 6, 'GLY': 6, 'ALA': 1, 'PRO': 1, 'CYS': 1, 'ILE': 1 | 3.2 ± 0.6  2.5 ± 0.6 | 133.7 ± 19.5 | N/A | N/A |
| **Water mediated H-bond** | 'GLU': 23, 'LYS': 21, 'SER': 13, 'THR': 11, 'GLN': 10, 'LEU': 9, 'GLY': 5, 'ALA': 5, 'ARG': 4, 'ASP': 3, 'TYR': 2, 'HIS': 2, 'CYS': 2, 'VAL': 1, 'ASN': 1} | 3.2 ± 0.4  3.4 ± 0.5 | 138.9 ± 22.3  93.5 ± 17.0 | N/A | N/A |
| **Salt bridge** | LYS': 3 | 4.6 ± 1.0 | N/A | N/A | N/A |
| **π-π stacking** | 'PHE': 25, 'TRP': 6, 'HIS': 5 | 4.6 ± 0.6 | 74.6 ± 10.5  10.6 ± 7.2 | 1.5 ± 0.4 | 'T': 20  'P': 16 |
| **π-cation interaction** | 'HIS': 5, 'LYS': 2 | 4.6 ± 0.5 | N/A | 1.9 ± 0.1 | N/A |
| **Metal complexation** | no | no | N/A | N/A | no |

**Table S9.** Overview of the main properties of coumarin interactions

| **Interaction type** | **Residues/metals involved** | **Average distance (Å)** | **Average angle (°)** | **Average offset (Å)** | **Geometry** |
| --- | --- | --- | --- | --- | --- |
| **Hydrophobic** | PHE': 18, 'ILE': 18, 'TRP': 16, 'ALA': 16, 'VAL': 10, 'LEU': 8, 'LYS': 8, 'ASN': 5, 'TYR': 4, 'ASP': 4, 'THR': 4, 'MET': 2, 'PRO': 2, 'GLU': 1 | 3.7 ± 0.2 | N/A | N/A | N/A |
| **Direct hydrogen bond** | SER': 5, 'ASN': 5, 'HIS': 4, 'CYS': 2, 'VAL': 2, 'ILE': 1 | 3.1 ± 0.3  2.3 ± 0.4 | 149.8 ± 17.2 | N/A | N/A |
| **Water mediated H-bond** | 'GLY': 2, 'LYS': 2 | 2.7 ± 0.1  3.45 ± 0.1 | 151.3 ± 16.9  74.7 ± 3.1 | N/A | N/A |
| **Salt bridge** | no | no | N/A | N/A | N/A |
| **π-π stacking** | 'PHE': 9, 'TRP': 4, 'HIS': 2 | 4.6 ± 0.6 | 80.6 ± 3.2  25.6 ± 2.9 | 0.9 ± 0.5 | 'T': 9  'P': 6 |
| **π-cation interaction** | no | no | N/A |  | N/A |
| **Metal complexation** | no | no | N/A | N/A | no |

**Table S10.** Overview of the main properties of coumestant interactions.

| **Interaction type** | **Residues/metals involved** | **Average distance (Å)** | **Average angle (°)** | **Average offset (Å)** | **Geometry** |
| --- | --- | --- | --- | --- | --- |
| **Hydrophobic** | 'ILE': 11, 'LEU': 10, 'PHE': 8, 'ALA': 5, 'VAL': 5, 'TYR': 3, 'LYS': 2 | 3.7 ± 0.2 | N/A | N/A | N/A |
| **Direct hydrogen bond** | 'ARG': 4, 'HIS': 4, 'LYS': 4, 'TYR': 3, 'ASN': 3, 'LEU': 2, 'GLU': 2, 'ASP': 2, 'GLY': 1 | 3.4 ± 0.5  2.7 ± 0.7 | 135.9 ± 19.2 | N/A | N/A |
| **Water mediated H-bond** | 'ASN': 4, 'LEU': 2 | 3.5 ± 0.2  2.9 ± 0.0 | 123.9 ± 16.6  80.7 ± 6.9 | N/A | N/A |
| **Salt bridge** | no | no | N/A | N/A | N/A |
| **π-π stacking** | 'TYR': 6, 'PHE': 4 | 4.3 ± 0.9 | 73.2 ± 0.0  5.6 ± 1.3 | 1.3 ± 0.5 | 'T': 4  P': 6, |
| **π-cation interaction** | no | no | no | no | N/A |
| **Metal complexation** | no | no | N/A | N/A | no |

**Table S11.** Overview of the main properties of lignan interactions.

| **Interaction type** | **Residues/metals involved** | **Average distance (Å)** | **Average angle (°)** | **Average offset (Å)** | **Geometry** |
| --- | --- | --- | --- | --- | --- |
| **Hydrophobic** | PHE': 10, 'VAL': 4, 'LEU': 3, 'TYR': 2, 'THR': 2, 'ALA': 1, 'ILE': 1 | 3.7 ± 0.2 | N/A | N/A | N/A |
| **Direct hydrogen bond** | MET': 12, 'GLY': 5, 'VAL': 4, 'ASN': 4, 'SER': 4, 'ARG': 4, 'LYS': 2, 'HIS': 2, 'GLN': 1, 'PRO': 1 | 3.3 ± 0.4  2.5 ± 0.5 | 146.4 ± 17.3 | N/A | N/A |
| **Water mediated H-bond** | 'GLY': 2, 'GLU': 2, 'MET': 2, 'HIS': 1, 'LYS': 1 | 2.9 ± 0.4  3.5 ± 0.4 | 143.1 ± 22.9  84.6 ± 6.9 | N/A | N/A |
| **Salt bridge** | no | no | N/A | N/A | N/A |
| **π-π stacking** | 'PHE': 14, 'HIS': 7 | 5.0 ± 0.3 | 78.1 ± 7.8  18.2 ± 7.2 | 1.5 ± 0.4 | 'T': 19  'P': 2 |
| **π-cation interaction** | no | no | no | no | N/A |
| **Metal complexation** | no | no | N/A | N/A | no |

**Table S12.** Overview of the main properties of naphtaquinone interactions.

| **Interaction type** | **Residues/metals involved** | **Average distance (Å)** | **Average angle (°)** | **Average offset (Å)** | **Geometry** |
| --- | --- | --- | --- | --- | --- |
| **Hydrophobic** | 'ARG': 12, 'TYR': 10, 'PHE': 8, 'LYS': 4, 'VAL': 3, 'PRO': 2, 'LEU': 2, 'ASP': 1, 'THR': 1, 'ALA': 1, 'TRP': 1 | 3.6 ± 0.2 | N/A | N/A | N/A |
| **Direct hydrogen bond** | 'LYS': 8, 'ARG': 7, 'TYR': 6, 'ASN': 3, 'GLN': 2, 'THR': 1, 'ALA': 1, 'VAL': 1 | 3.0 ± 0.3  2.3 ± 0.4 | 139.4 ± 16.3 | N/A | N/A |
| **Water mediated H-bond** | ARG': 11, 'GLY': 10, 'SER': 5, 'THR': 4, 'HIS': 4, 'TYR': 4, 'PRO': 2, 'GLU': 2, 'ASN': 1 | 3.6 ± 0.4  3.4 ± 0.5 | 128.1 ± 15.4  84.3 ± 11.2 | N/A | N/A |
| **Salt bridge** | ARG': 8 | 4.2 ± 0.1 | N/A | N/A | N/A |
| **π-π stacking** | TYR': 3, 'HIS': 2, 'PHE': 2, 'TRP': 1 | 4.6 ± 0.5 | 74.6 ± 10.7  11.0 ± 3.4 | 1.4 ± 0.5 | 'T': 5  'P': 3 |
| **π-cation interaction** | LYS': 2 | 4.6 ± 0.0 | N/A | 0.6 ± 0.0 | N/A |
| **Metal complexation** | no | no | N/A | N/A | no |

**Table S13.** Overview of the main properties of curcuminoid interactions.

| **Interaction type** | **Residues/metals involved** | **Average distance (Å)** | **Average angle (°)** | **Average offset (Å)** | **Geometry** |
| --- | --- | --- | --- | --- | --- |
| **Hydrophobic** | 'ILE': 13, 'LEU': 11, 'ALA': 5, 'PHE': 3, 'THR': 1, 'PRO': 1, 'VAL': 1 | 3.7 ± 0.2 | N/A | N/A | N/A |
| **Direct hydrogen bond** | 'LYS': 9, 'SER': 9, 'ASP': 5, 'PHE': 3, 'GLY': 3, 'THR': 3, 'LEU': 2, 'GLU': 1 | 3.4 ± 0.5  2.8 ± 0.6 | 131.1 ± 20.3 | N/A | N/A |
| **Water mediated H-bond** | 'LYS': 5, 'THR': 2 | 3.3 ± 0.5  3.5 ± 0.3 | 140.7 ± 20.1  118.1 ± 15.8 | N/A | N/A |
| **Salt bridge** | LYS': 2 | 2.9 ± 0.1 | N/A | N/A | N/A |
| **π-π stacking** | 'PHE': 2 | 4.2 ± 0.0 | 28.2 ± 0.0 | 1.9 ± 0.0 | P': 2 |
| **π-cation interaction** | no | no | no | no | N/A |
| **Metal complexation** | no | no | N/A | N/A | no |

**Table S14.** Overview of the main properties of calchone interactions.

| **Interaction type** | **Residues/metals involved** | **Average distance (Å)** | **Average angle (°)** | **Average offset (Å)** | **Geometry** |
| --- | --- | --- | --- | --- | --- |
| **Hydrophobic** | 'PHE': 37, 'LEU': 14, 'THR': 9, 'ILE': 8, 'VAL': 8, 'TYR': 7, 'TRP': 6, 'GLN': 4, 'PRO': 4, 'ASP': 2, 'ARG': 1, 'LYS': 1, 'ALA': 1 | 3.8 ± 0.2 | N/A | N/A | N/A |
| **Direct hydrogen bond** | PHE': 37, 'LEU': 14, 'THR': 9, 'ILE': 8, 'VAL': 8, 'TYR': 7, 'TRP': 6, 'GLN': 4, 'PRO': 4, 'ASP': 2, 'ARG': 1, 'LYS': 1, 'ALA': 1 | 3.3 ± 0.5  2.6 ± 0.6 | 131.7 ± 19.4 | N/A | N/A |
| **Water mediated H-bond** | ASN': 12, 'HIS': 4, 'SER': 4, 'ARG': 2, 'GLN': 2, 'CYS': 2, 'ASP': 2, 'LYS': 1 | 3.6 ± 0.3  3.3 ± 0.4 | 129.7 ± 20.7  90.8 ± 10.5 | N/A | N/A |
| **Salt bridge** | no | no | N/A | N/A | N/A |
| **π-π stacking** | 'TYR': 4, 'PHE': 3 | 4.9 ± 0.1 | 71.9 ± 6.9 | 0.9 ± 0.6 | 'T': 7  'P': 0 |
| **π-cation interaction** | ARG': 2, 'HIS' : 1 | 3.9 ± 1.1 | N/A | 0.4 ± 0.4 | N/A |
| **Metal complexation** | no | no | N/A | N/A | no |

**Table S15.** Overview of the main properties of compounds not classified into a specific polyphenol group (*others*).

| **Interaction type** | **Residues/metals involved** | **Average distance (Å)** | **Average angle (°)** | **Average offset (Å)** | **Geometry** |
| --- | --- | --- | --- | --- | --- |
| **Hydrophobic** | 'LEU': 48, 'PHE': 38, 'TYR': 21, 'ALA': 20, 'THR': 12, 'ILE': 10, 'LYS': 6, 'VAL': 5, 'TRP': 5, 'GLN': 4, 'MET': 4, 'ASN': 2, 'PRO': 2, 'GLU': 2, 'ARG': 1, 'ASP': 1} | 3.7 ± 0.2 | N/A | N/A | N/A |
| **Direct hydrogen bond** | 'ASP': 26, 'ARG': 18, 'SER': 14, 'GLU': 11, 'GLN': 10, 'ASN': 8, 'TYR': 7, 'GLY': 5, 'MET': 4, 'HIS': 3, 'LYS': 2, 'ALA': 2, 'LEU': 1} | 3.2 ± 0.5  2.5 ± 0.7 | 137.0 ± 20.0 | N/A | N/A |
| **Water mediated H-bond** | 'ASN': 5, 'ASP': 5, 'GLU': 4, 'THR': 4, 'LYS': 4, 'SER': 4, 'ARG': 4, 'VAL': 3, 'ALA': 2, 'LEU': 2, 'PHE': 2, 'TYR': 2, 'GLY': 2, 'HIS': 1} | 3.3 ± 0.4  3.3 ± 0.5 | 140.2 ± 24.8  87.8 ± 14.5 | N/A | N/A |
| **Salt bridge** | 'ARG': 17, 'LYS': 2 | 3.9 ± 0.6 | N/A | N/A | N/A |
| **π-π stacking** | TYR': 20, 'PHE': 12, 'TRP': 4, 'HIS': 4 | 4.1 ± 0.5 | 75.6 ± 5.4  8.3 ± 4.8 | 1.3 ± 0.4 | 'T': 10  'P': 30 |
| **π-cation interaction** | 'LYS': 2, 'HIS': 2 | 4.7 ± 0.2 | N/A | 1.4 ± 0.6 | N/A |
| **Metal complexation** | 'Mg': 4, 'Fe': 2, 'Co': 2 | 2.0 ± 0.7 | N/A | N/A | 'square.pyramidal': 6, 'tetrahedral': 2 |

**Figure S1.** Normalized radial distributions for all protein atom – polyphenol atom pairs exhibiting more than 1000 occurrences. The distances are reported in Angstromes.

**Figure S2.** Noncovalent interactions within the PcaY_PP binding site (PDB ID 6S33) involving protocatechuate (DHB). DHB interacts via various noncovalent interactions, representing direct H-bonds by purple lines, and water-mediated H-bonds by cyan lines. Salt bridges are represented by light-orange lines, and hydrophobic interactions by grey lines. The protein is depicted using sky blue cartoons, with highlighted amino acid residues shown with grey sticks. DHB carbons are shown in green sticks.

**Figure S3.** Flavonoids can form a variety of noncovalent interactions. **(a)** Flavonoid luteolin interactions with transthyretin. Luteolin carbons are shown with green sticks. **(b)** Orientin interactions with influenza A endonuclease. Orientin carbons are presented with light-green sticks. Purple lines represents H-bonds, cyan lines water-mediated H-bonds, orange lines π-cation interactions, green lines metal complexation and grey lines hydrophobic interactions. The proteins are shown using skyblue cartoons and gray sticks for carbons of highlighted amino-acid residues.

**Figure S4.** Interactions between human protein kinase CK2 (blue cartoons and gray carbons of amino-acid residues) and the hydroxycinnamic acid derivative ferulic acid (FER, green carbons). Yellow dotted lines represent a salt-bridge interaction, purple line represent direct H-bonds, cyan lines water-mediated H-bonds, and grey lines hydrophobic interactions.

**Figure S5.** Catechol (CAQ, green carbons) noncovalent interactions at the interface of a urease enzyme (blue cartoons and grey carbons of amino-acid residues). Cyan dotted lines represent water-mediated H-bonds, and gray lines hydrophobic interactions.

**Figure S6.** Crystal structure of FMN quinone reductase 2 in complex with resveratrol at high resolution. Resveratrol (STL) binds between chains A and B. Purple lines represent H-bonds, cyan lines water-mediated H-bonds, orange lines π-stacking interactions, and grey lines hydrophobic interactions. The protein FMN quinone reductase 2 is shown with skyblue cartoons and grey sticks for carbons of highlighted aminoacid residues. Resveratrol is shown with green sticks for carbons. Resveratrol also forms π-stacking interactions with flavin mononucleotide (FMN, gray) (interactions not shown).

**Figure 7.** Umbelliferone (green sticks for carbon) binding to COVID-19 non-structural protein (blue cartoons and grey lines for amino-acid carbon). Cyan lines water-mediated H-bonds, orange lines π-stacking interactions, and gray lines hydrophobic interactions.

**Figure S8.** Examples of polyphenol-protein interactions of polyphenol classes less represented in the PDB. **(a)** Coumestrol (CUE) bound to human protein kinase CK2 alpha. **(b)** Lariciresinol (GFU) bound to pinoresinol-lariciresinol reductases. **(c)** Juglone (JUG) bound to RNase P 1. **(d)** Curcumin (CUR) bound to transthyretin. **(e)** Isoliquiritigenin bound to O-ethyltransferase, and **(f)** mandelic acid (SMN) bound to mandelate racemase. Purple dotted lines represent H-bonds, cyan lines water-mediated H-bonds, dark orange lines π-stacking interactions, yellow lines π-cation interactions, green lines metal complexation, and grey lines hydrophobic interactions. Polyphenols are shown with green sticks, and proteins with blue cartoons and grey sticks for amino-acid carbons.

**Figure S9**. RMSD timeseries of the glutamate dehydrogenase-epicatechin-3-gallate (XEG) simulation, for (a) the main simulation, and (b) replica simulation. Highlights represent distinct clusters described in the main text. The (c) initial crystal structure contains no water molecules and displays (d) only direct H-bonding interactions with XEG. (e) Bridge2 output of the H-bonding interactions of the crystal structure. (f) Bridge2 output of the XEG water-mediated H-bonding for the first (f) and second (g) cluster of the replica simulation. Values on edges represent the average numbering of bridging water molecules during the MD simulation.

**Figure S10.** Three GHD conformations overlap in cartoon model representation colored green (first), cyan(transition) and magenta (second) with XEG ligand in yellow colored stick model with emphasized binding site contacts in red color.

**Figure S11.** Epicatechin-3-gallate (XEG) depicted in blue-colored stick model with surrounding glutamate dehydrogenase residues in yellow colored stick model with emphasized atoms. Red spheres represent identified water clusters where TIP3P is constantly present through the trajectory (main; 0-600 ns). Water cluster-neighbor residue distances are also presented in magenta dotted lines. Water clusters can be observed also at the locations bridging ligand contacts towards Asp119, Glu487, Hsd85, Arg86, Lys387, Asn388, His209 and Ser393 as observed beforehand.

**Figure S12**. RMSD plots for (a) the main simulation starting from the low resolution structure (1DVS), (b) the replica simulation starting from the low resolution structure, (c) the main simulation starting from the high resolution (7Q9O) structure, and (d) the replica simulation starting from the high resolution structure. Interaction contact maps between transthyretin residues and STL for the main (e) and replica (f) simulations started from the low resolution structure, and main (g), and replica (h) started from the high-resolution structure depicting the presence of hydrophobic (yellow), hydrogen bonds (green), and rare π-cation (purple) interactions. Water interaction networks obtained with Bridge2 formed by STL in the (i) replica simulation starting from the low resolution structure, (j) main simulation starting from the high resolution structure, (k) replica simulation starting from the high resolution structure. The occupancy of these water-mediated H-bonds is at least 50%. The corresponding network of the main simulation of the low resolution structure is presented in the main text.

**Figure S13**. RMSD values for SIRT6 dimer simulations. (a) main simulation when aglycone (QUE) is bound to SIRT6, (b) aglycone replica simulation, (c) glycon (HW2) main simulation, and (d) glycon replica simulation. Interaction contact maps between SIRT6 residues and QUE for the main (e) and replica (f) simulations, and between SIRT6 and HW2 for the main (g) and replica (h) simulation, depicting the presence of hydrophobic (yellow), hydrogen bonds (green) interactions.

-------------------------

MADE plugin REPORT file

-------------------------

Examined complex: frame0000

Whole chain setting used: False

Whole chain selection: / (not used)

Binding site selection: XEG.601.P

Chain selection: P

Used a custom structure with a custom clusters of complexes

Unique structures in identified cluster: ['frame0000', 'frame0010', 'frame0020', 'frame0030', 'frame0040', 'frame0050', 'frame0060', 'frame0070', 'frame0080', 'frame0090', 'frame0100', 'frame0110', 'frame0120', 'frame0130', 'frame0140', 'frame0150', 'frame0160', 'frame0170', 'frame0180', 'frame0190', 'frame0200', 'frame0210', 'frame0220', 'frame0230', 'frame0240', 'frame0250', 'frame0260', 'frame0270', 'frame0280', 'frame0290', 'frame0300', 'frame0310', 'frame0320', 'frame0330', 'frame0340', 'frame0350', 'frame0360', 'frame0370', 'frame0380', 'frame0390', 'frame0400', 'frame0410', 'frame0420', 'frame0430', 'frame0440', 'frame0450', 'frame0460', 'frame0470', 'frame0480', 'frame0490', 'frame0500', 'frame0510', 'frame0520', 'frame0530', 'frame0540', 'frame0550', 'frame0560', 'frame0570', 'frame0580', 'frame0590', 'frame0600']

Resolution of complexes [in Å]:

{}

Average Resolution of complexes in cluster: nan ± nan Å

Performing protein superposition using the align command in PyMOL: https://pymolwiki.org/index.php/Align

System volume is: 5708 cubic A

HETATM CLUSTERING------------

Clustering of heteroatoms with DBSCAN, epslilon = 0.9 A

Binding site info (name, avg x, y, z, min x, max x, min y, max y, min z, max z; box 4.0 A around extremes):

['XEG.601.P', 3.86834, -1.83824, 4.91942, 0.682, 9.076, -8.25, 3.946, 2.08, 11.32]

Examined clusters with 16 or more TIP-OH2 heteroatoms

-------------------------

Cluster nr. 0, composed of 51 TIP-OH2 heteroatoms, conservation 0.836:

(x y z iso_disp serial_number chain_identifier hetatm_type hetatom_name sequence_number PDB_id)

9.707 -0.316 10.799 0.0 69519 S TIP OH2 4675 frame0100

8.361 -1.295 11.337 0.0 53505 S TIP OH2 9337 frame0490

9.875 -0.454 11.064 0.0 55194 S TIP OH2 9900 frame0240

8.098 -0.441 10.964 0.0 55401 S TIP OH2 9969 frame0590

9.872 -0.439 10.855 0.0 54894 S TIP OH2 9800 frame0140

8.847 -0.225 10.747 0.0 77772 S TIP OH2 7426 frame0360

10.468 -0.353 10.563 0.0 74940 S TIP OH2 6482 frame0070

8.631 -1.671 10.772 0.0 65757 S TIP OH2 3421 frame0010

10.213 -0.125 10.598 0.0 52329 S TIP OH2 8945 frame0430

8.643 -0.076 10.594 0.0 68895 S TIP OH2 4467 frame0260

8.521 -0.967 11.127 0.0 61680 S TIP OH2 2062 frame0340

9.357 -1.006 10.581 0.0 75723 S TIP OH2 6743 frame0020

10.035 -0.263 10.172 0.0 68508 S TIP OH2 4338 frame0110

10.015 -0.295 10.933 0.0 54009 S TIP OH2 9505 frame0330

8.12 -0.363 10.259 0.0 65058 S TIP OH2 3188 frame0460

9.61 -0.28 9.537 0.0 67503 S TIP OH2 4003 frame0040

8.338 -0.078 10.054 0.0 56808 S TIP OH2 438 frame0520

10.09 -0.179 10.332 0.0 65676 S TIP OH2 3394 frame0300

8.753 -0.535 11.219 0.0 78720 S TIP OH2 7742 frame0540

10.099 -0.263 10.307 0.0 78126 S TIP OH2 7544 frame0440

10.035 0.06 10.233 0.0 67743 S TIP OH2 4083 frame0270

10.964 0.027 10.583 0.0 67743 S TIP OH2 4083 frame0280

8.898 -0.255 10.834 0.0 61395 S TIP OH2 1967 frame0230

9.783 0.122 10.575 0.0 61686 S TIP OH2 2064 frame0130

9.213 -1.121 10.725 0.0 79890 S TIP OH2 8132 frame0530

10.013 -0.076 11.084 0.0 54975 S TIP OH2 9827 frame0170

9.816 -0.442 10.651 0.0 55431 S TIP OH2 9979 frame0060

9.685 0.364 10.398 0.0 54009 S TIP OH2 9505 frame0310

8.428 -1.787 11.16 0.0 53787 S TIP OH2 9431 frame0380

10.057 -0.045 11.265 0.0 53244 S TIP OH2 9250 frame0290

8.852 -1.683 11.359 0.0 57378 S TIP OH2 628 frame0120

9.126 -0.975 10.429 0.0 56049 S TIP OH2 185 frame0500

9.681 -0.47 11.017 0.0 54975 S TIP OH2 9827 frame0180

10.041 -0.096 10.694 0.0 54009 S TIP OH2 9505 frame0320

9.076 -0.054 11.431 0.0 77772 S TIP OH2 7426 frame0370

8.4 -1.201 11.078 0.0 68160 S TIP OH2 4222 frame0410

10.515 0.222 10.499 0.0 75651 S TIP OH2 6719 frame0190

9.412 -0.8 11.103 0.0 57744 S TIP OH2 750 frame0210

10.315 -0.076 10.395 0.0 55107 S TIP OH2 9871 frame0480

8.828 -0.121 10.68 0.0 64104 S TIP OH2 2870 frame0050

10.072 -0.27 10.789 0.0 56886 S TIP OH2 464 frame0150

8.187 -0.163 11.023 0.0 68238 S TIP OH2 4248 frame0420

9.365 -0.246 11.13 0.0 75651 S TIP OH2 6719 frame0200

10.13 -0.087 10.259 0.0 73668 S TIP OH2 6058 frame0080

9.507 -1.054 10.635 0.0 78360 S TIP OH2 7622 frame0580

8.858 -1.482 11.073 0.0 74490 S TIP OH2 6332 frame0220

9.212 -0.638 10.884 0.0 64956 S TIP OH2 3154 frame0450

8.263 -1.054 11.925 0.0 50367 S TIP OH2 8291 frame0250

8.662 -1.67 10.871 0.0 56406 S TIP OH2 304 frame0390

8.382 -0.653 10.21 0.0 73041 S TIP OH2 5849 frame0470

9.216 -0.471 10.103 0.0 51063 S TIP OH2 8523 frame0090

Cluster 0 Averege pos.: 9.346 -0.506 10.743

Cluster 0 Averege st. dev.: 0.735 0.533 0.422

Cluster nr. 1, composed of 43 TIP-OH2 heteroatoms, conservation 0.705:

(x y z iso_disp serial_number chain_identifier hetatm_type hetatom_name sequence_number PDB_id)

4.402 -5.388 9.545 0.0 80178 S TIP OH2 8228 frame0100

4.659 -5.525 9.12 0.0 69459 S TIP OH2 4655 frame0240

4.351 -4.529 9.626 0.0 58317 S TIP OH2 941 frame0140

4.813 -5.756 9.352 0.0 63336 S TIP OH2 2614 frame0360

3.904 -4.778 9.431 0.0 75783 S TIP OH2 6763 frame0070

2.851 -4.814 9.935 0.0 61602 S TIP OH2 2036 frame0010

5.374 -5.749 8.573 0.0 53322 S TIP OH2 9276 frame0010

4.814 -5.72 9.72 0.0 63264 S TIP OH2 2590 frame0430

5.066 -5.862 9.003 0.0 61494 S TIP OH2 2000 frame0260

5.028 -5.28 9.485 0.0 51537 S TIP OH2 8681 frame0340

4.435 -5.246 10.04 0.0 50691 S TIP OH2 8399 frame0020

4.659 -5.626 9.401 0.0 59391 S TIP OH2 1299 frame0110

4.318 -4.38 8.856 0.0 59970 S TIP OH2 1492 frame0330

4.117 -6.518 10.557 0.0 64707 S TIP OH2 3071 frame0330

3.902 -5.233 9.425 0.0 70278 S TIP OH2 4928 frame0460

3.395 -5.832 10.283 0.0 56220 S TIP OH2 242 frame0040

3.712 -5.037 9.964 0.0 77319 S TIP OH2 7275 frame0300

4.102 -5.683 9.578 0.0 58482 S TIP OH2 996 frame0270

4.356 -5.369 9.906 0.0 79347 S TIP OH2 7951 frame0230

3.449 -6.052 9.243 0.0 64719 S TIP OH2 3075 frame0350

4.356 -4.907 9.583 0.0 59127 S TIP OH2 1211 frame0170

3.676 -5.286 9.798 0.0 76173 S TIP OH2 6893 frame0310

4.215 -4.934 8.838 0.0 80103 S TIP OH2 8203 frame0380

4.869 -5.049 9.796 0.0 64464 S TIP OH2 2990 frame0290

3.984 -5.872 10.014 0.0 69753 S TIP OH2 4753 frame0120

4.686 -4.841 9.55 0.0 80145 S TIP OH2 8217 frame0500

3.642 -4.711 9.863 0.0 73020 S TIP OH2 5842 frame0180

4.004 -5.304 9.975 0.0 62919 S TIP OH2 2475 frame0320

3.507 -4.671 10.695 0.0 59724 S TIP OH2 1410 frame0030

5.166 -5.639 8.816 0.0 53904 S TIP OH2 9470 frame0030

4.396 -6.344 9.455 0.0 72954 S TIP OH2 5820 frame0370

3.999 -5.987 9.373 0.0 63324 S TIP OH2 2610 frame0410

3.738 -5.462 10.254 0.0 63294 S TIP OH2 2600 frame0190

3.076 -5.448 10.013 0.0 54867 S TIP OH2 9791 frame0050

3.473 -4.823 10.257 0.0 58722 S TIP OH2 1076 frame0150

4.106 -5.326 8.814 0.0 67902 S TIP OH2 4136 frame0420

4.674 -6.162 9.209 0.0 66447 S TIP OH2 3651 frame0200

4.477 -5.266 9.147 0.0 55596 S TIP OH2 34 frame0080

5.502 -5.107 8.869 0.0 75336 S TIP OH2 6614 frame0220

4.69 -5.937 9.049 0.0 58713 S TIP OH2 1073 frame0160

3.407 -6.266 9.826 0.0 65331 S TIP OH2 3279 frame0250

4.839 -6.095 9.051 0.0 51828 S TIP OH2 8778 frame0400

3.273 -6.266 9.764 0.0 51603 S TIP OH2 8703 frame0090

Cluster 1 Averege pos.: 4.22 -5.444 9.559

Cluster 1 Averege st. dev.: 0.622 0.527 0.494

Cluster nr. 2, composed of 27 TIP-OH2 heteroatoms, conservation 0.443:

(x y z iso_disp serial_number chain_identifier hetatm_type hetatom_name sequence_number PDB_id)

2.047 2.515 3.697 0.0 61203 S TIP OH2 1903 frame0100

2.328 2.726 3.677 0.0 66915 S TIP OH2 3807 frame0240

3.11 2.616 2.89 0.0 79971 S TIP OH2 8159 frame0070

2.727 2.411 3.062 0.0 61992 S TIP OH2 2166 frame0010

2.45 2.96 4.394 0.0 77508 S TIP OH2 7338 frame0430

2.081 2.673 3.688 0.0 68139 S TIP OH2 4215 frame0260

2.448 2.919 3.317 0.0 50490 S TIP OH2 8332 frame0520

2.973 2.957 3.197 0.0 51357 S TIP OH2 8621 frame0300

3.163 2.935 2.513 0.0 77676 S TIP OH2 7394 frame0440

2.749 2.985 3.136 0.0 62778 S TIP OH2 2428 frame0270

2.788 3.019 3.168 0.0 56184 S TIP OH2 230 frame0350

2.209 3.083 3.29 0.0 68814 S TIP OH2 4440 frame0530

2.468 2.964 3.011 0.0 50583 S TIP OH2 8363 frame0170

1.948 2.665 2.71 0.0 57297 S TIP OH2 601 frame0060

2.995 2.739 2.496 0.0 75282 S TIP OH2 6596 frame0310

2.205 1.908 3.321 0.0 70974 S TIP OH2 5160 frame0120

2.239 2.126 4.021 0.0 72873 S TIP OH2 5793 frame0550

1.859 2.267 2.908 0.0 50577 S TIP OH2 8361 frame0500

1.683 2.646 4.142 0.0 69993 S TIP OH2 4833 frame0180

2.155 2.071 3.26 0.0 53409 S TIP OH2 9305 frame0600

2.399 2.017 2.628 0.0 77394 S TIP OH2 7300 frame0320

1.995 2.833 4.452 0.0 78987 S TIP OH2 7831 frame0410

1.752 2.708 3.636 0.0 65640 S TIP OH2 3382 frame0200

2.343 2.086 2.408 0.0 76563 S TIP OH2 7023 frame0580

3.105 2.753 4.128 0.0 51159 S TIP OH2 8555 frame0220

3.567 2.547 3.255 0.0 68130 S TIP OH2 4212 frame0390

2.46 1.973 3.397 0.0 57036 S TIP OH2 514 frame0090

Cluster 2 Averege pos.: 2.454 2.596 3.326

Cluster 2 Averege st. dev.: 0.469 0.357 0.557

Cluster nr. 3, composed of 46 TIP-OH2 heteroatoms, conservation 0.754:

(x y z iso_disp serial_number chain_identifier hetatm_type hetatom_name sequence_number PDB_id)

3.582 -0.158 2.883 0.0 70446 S TIP OH2 4984 frame0100

4.303 -0.58 2.554 0.0 50979 S TIP OH2 8495 frame0240

3.345 0.631 3.595 0.0 69603 S TIP OH2 4703 frame0590

4.52 0.382 3.233 0.0 77616 S TIP OH2 7374 frame0140

4.43 0.386 2.758 0.0 79404 S TIP OH2 7970 frame0360

4.409 0.241 2.82 0.0 67059 S TIP OH2 3855 frame0070

4.426 0.327 3.116 0.0 76893 S TIP OH2 7133 frame0010

3.84 0.498 3.179 0.0 58116 S TIP OH2 874 frame0260

2.679 0.175 3.552 0.0 51105 S TIP OH2 8537 frame0020

3.128 1.223 3.33 0.0 70224 S TIP OH2 4910 frame0330

2.983 0.105 4.591 0.0 66696 S TIP OH2 3734 frame0460

4.112 0.593 3.323 0.0 61242 S TIP OH2 1916 frame0040

4.405 0.764 3.237 0.0 69258 S TIP OH2 4588 frame0300

2.965 0.236 4.43 0.0 56487 S TIP OH2 331 frame0540

3.269 -0.074 4.682 0.0 64200 S TIP OH2 2902 frame0560

5.312 0.231 2.877 0.0 56391 S TIP OH2 299 frame0440

4.365 0.603 3.4 0.0 63465 S TIP OH2 2657 frame0270

2.765 0.217 4.679 0.0 74490 S TIP OH2 6332 frame0230

4.576 0.471 3.57 0.0 66849 S TIP OH2 3785 frame0130

4.548 0.938 3.804 0.0 71919 S TIP OH2 5475 frame0350

3.033 0.243 4.229 0.0 71514 S TIP OH2 5340 frame0170

2.189 0.193 3.818 0.0 75372 S TIP OH2 6626 frame0060

3.993 0.047 2.353 0.0 54285 S TIP OH2 9597 frame0310

4.077 0.392 3.048 0.0 65280 S TIP OH2 3262 frame0380

3.335 0.64 4.107 0.0 68526 S TIP OH2 4344 frame0290

3.192 -0.558 3.793 0.0 75888 S TIP OH2 6798 frame0120

3.644 0.318 3.188 0.0 55845 S TIP OH2 117 frame0500

3.296 -0.437 3.413 0.0 57954 S TIP OH2 820 frame0600

1.997 -0.215 3.997 0.0 69411 S TIP OH2 4639 frame0030

4.604 0.209 2.559 0.0 52455 S TIP OH2 8987 frame0030

3.564 0.219 3.743 0.0 64827 S TIP OH2 3111 frame0410

3.123 0.382 3.557 0.0 75930 S TIP OH2 6812 frame0190

2.177 -0.445 3.451 0.0 62505 S TIP OH2 2337 frame0210

4.767 -0.416 2.426 0.0 54192 S TIP OH2 9566 frame0210

3.602 0.154 4.174 0.0 67626 S TIP OH2 4044 frame0480

2.768 0.109 3.699 0.0 61500 S TIP OH2 2002 frame0200

3.235 -0.095 4.093 0.0 61047 S TIP OH2 1851 frame0080

3.312 -0.132 3.83 0.0 53832 S TIP OH2 9446 frame0580

2.876 -0.813 4.115 0.0 51633 S TIP OH2 8713 frame0220

3.018 0.789 4.485 0.0 75729 S TIP OH2 6745 frame0570

3.051 0.892 4.215 0.0 73473 S TIP OH2 5993 frame0450

3.711 0.092 4.011 0.0 60837 S TIP OH2 1781 frame0250

2.083 0.103 4.04 0.0 65004 S TIP OH2 3170 frame0390

4.455 0.133 2.341 0.0 73860 S TIP OH2 6122 frame0390

4.355 0.199 2.87 0.0 59232 S TIP OH2 1246 frame0400

4.616 0.101 3.471 0.0 75240 S TIP OH2 6582 frame0090

Cluster 3 Averege pos.: 3.609 0.202 3.536

Cluster 3 Averege st. dev.: 0.799 0.413 0.629

Cluster nr. 4, composed of 30 TIP-OH2 heteroatoms, conservation 0.492:

(x y z iso_disp serial_number chain_identifier hetatm_type hetatom_name sequence_number PDB_id)

5.289 -9.139 5.906 0.0 54081 S TIP OH2 9529 frame0240

5.179 -8.343 5.555 0.0 75585 S TIP OH2 6697 frame0590

5.072 -9.087 5.097 0.0 75042 S TIP OH2 6516 frame0360

4.708 -9.079 5.386 0.0 63204 S TIP OH2 2570 frame0260

4.243 -8.911 5.003 0.0 78576 S TIP OH2 7694 frame0460

4.042 -8.876 5.488 0.0 53556 S TIP OH2 9354 frame0040

5.391 -8.307 5.913 0.0 51120 S TIP OH2 8542 frame0520

4.587 -8.681 5.107 0.0 69369 S TIP OH2 4625 frame0540

5.103 -9.242 4.583 0.0 62772 S TIP OH2 2426 frame0440

5.586 -8.757 5.484 0.0 78408 S TIP OH2 7638 frame0230

5.279 -9.389 4.906 0.0 75897 S TIP OH2 6801 frame0130

5.042 -9.808 4.925 0.0 76053 S TIP OH2 6853 frame0350

4.75 -7.77 6.257 0.0 80202 S TIP OH2 8236 frame0170

4.81 -7.867 6.317 0.0 51771 S TIP OH2 8759 frame0290

4.768 -9.089 5.277 0.0 54822 S TIP OH2 9776 frame0120

4.704 -8.34 5.762 0.0 78705 S TIP OH2 7737 frame0550

5.291 -8.469 5.106 0.0 75414 S TIP OH2 6640 frame0500

4.066 -8.042 5.697 0.0 61341 S TIP OH2 1949 frame0180

4.413 -9.341 5.304 0.0 59256 S TIP OH2 1254 frame0370

4.698 -9.078 5.446 0.0 52884 S TIP OH2 9130 frame0190

4.719 -8.931 6.381 0.0 77319 S TIP OH2 7275 frame0210

4.353 -8.202 5.933 0.0 58413 S TIP OH2 973 frame0480

4.299 -9.343 4.964 0.0 74682 S TIP OH2 6396 frame0420

4.845 -8.68 6.158 0.0 60846 S TIP OH2 1784 frame0580

5.314 -9.162 5.711 0.0 66879 S TIP OH2 3795 frame0570

4.931 -7.875 5.671 0.0 58323 S TIP OH2 943 frame0450

5.474 -7.828 5.552 0.0 77670 S TIP OH2 7392 frame0510

4.647 -9.46 4.893 0.0 75474 S TIP OH2 6660 frame0250

4.949 -9.318 4.942 0.0 51615 S TIP OH2 8707 frame0400

4.877 -8.048 5.172 0.0 75558 S TIP OH2 6688 frame0470

Cluster 4 Averege pos.: 4.848 -8.749 5.463

Cluster 4 Averege st. dev.: 0.403 0.56 0.467

Cluster nr. 5, composed of 20 TIP-OH2 heteroatoms, conservation 0.328:

(x y z iso_disp serial_number chain_identifier hetatm_type hetatom_name sequence_number PDB_id)

3.143 -6.768 7.387 0.0 52074 S TIP OH2 8860 frame0240

2.442 -6.228 7.617 0.0 51426 S TIP OH2 8644 frame0590

2.512 -6.34 8.092 0.0 76083 S TIP OH2 6863 frame0140

2.781 -7.016 7.655 0.0 55557 S TIP OH2 21 frame0360

2.546 -6.439 7.765 0.0 56265 S TIP OH2 257 frame0070

2.728 -6.522 7.525 0.0 60963 S TIP OH2 1823 frame0430

2.344 -6.583 8.101 0.0 80202 S TIP OH2 8236 frame0110

3.135 -7.736 7.476 0.0 75099 S TIP OH2 6535 frame0520

2.724 -5.799 7.5 0.0 69240 S TIP OH2 4582 frame0440

3.335 -6.615 7.997 0.0 54453 S TIP OH2 9653 frame0290

2.464 -5.549 7.864 0.0 78321 S TIP OH2 7609 frame0550

2.409 -6.885 8.253 0.0 66357 S TIP OH2 3621 frame0600

2.388 -6.81 8.144 0.0 71685 S TIP OH2 5397 frame0320

2.91 -6.998 7.216 0.0 53241 S TIP OH2 9249 frame0410

3.021 -5.868 7.199 0.0 65529 S TIP OH2 3345 frame0150

2.97 -7.257 6.864 0.0 56226 S TIP OH2 244 frame0200

2.697 -6.983 7.669 0.0 59367 S TIP OH2 1291 frame0080

2.178 -6.225 7.553 0.0 76845 S TIP OH2 7117 frame0220

2.373 -7.342 8.22 0.0 67710 S TIP OH2 4072 frame0400

2.453 -6.964 7.197 0.0 63159 S TIP OH2 2555 frame0090

Cluster 5 Averege pos.: 2.678 -6.646 7.665

Cluster 5 Averege st. dev.: 0.311 0.532 0.379

Cluster nr. 6, composed of 28 TIP-OH2 heteroatoms, conservation 0.459:

(x y z iso_disp serial_number chain_identifier hetatm_type hetatom_name sequence_number PDB_id)

4.588 1.191 6.671 0.0 63681 S TIP OH2 2729 frame0100

4.623 2.638 6.978 0.0 54570 S TIP OH2 9692 frame0490

4.357 1.935 7.516 0.0 72660 S TIP OH2 5722 frame0240

4.902 2.178 7.683 0.0 55872 S TIP OH2 126 frame0360

5.419 2.025 8.023 0.0 56766 S TIP OH2 424 frame0070

4.654 1.299 7.416 0.0 74796 S TIP OH2 6434 frame0010

4.216 1.764 6.216 0.0 75801 S TIP OH2 6769 frame0260

4.417 1.787 6.95 0.0 79545 S TIP OH2 8017 frame0340

4.571 0.779 7.677 0.0 65571 S TIP OH2 3359 frame0040

4.236 1.351 7.225 0.0 59562 S TIP OH2 1356 frame0300

4.291 1.144 7.944 0.0 50808 S TIP OH2 8438 frame0530

4.368 1.378 7.101 0.0 59982 S TIP OH2 1496 frame0060

4.905 1.474 7.562 0.0 73482 S TIP OH2 5996 frame0310

3.452 1.341 7.022 0.0 56259 S TIP OH2 255 frame0180

4.668 1.405 7.501 0.0 62757 S TIP OH2 2421 frame0320

5.766 1.397 7.553 0.0 62148 S TIP OH2 2218 frame0030

3.905 1.298 7.78 0.0 76122 S TIP OH2 6876 frame0410

4.21 1.092 7.969 0.0 78990 S TIP OH2 7832 frame0190

4.403 1.911 7.56 0.0 58368 S TIP OH2 958 frame0050

4.373 2.312 6.853 0.0 68616 S TIP OH2 4374 frame0420

4.54 1.974 7.291 0.0 75909 S TIP OH2 6805 frame0220

5.5 1.917 7.694 0.0 79323 S TIP OH2 7943 frame0570

4.114 1.235 7.503 0.0 62916 S TIP OH2 2474 frame0450

4.224 2.56 7.437 0.0 74931 S TIP OH2 6479 frame0250

4.314 1.463 6.515 0.0 69750 S TIP OH2 4752 frame0390

4.959 1.898 7.366 0.0 70404 S TIP OH2 4970 frame0400

5.493 1.815 7.348 0.0 75687 S TIP OH2 6731 frame0470

4.716 1.188 6.786 0.0 68512 S TIP OH2 1006 frame0000

Cluster 6 Averege pos.: 4.578 1.634 7.326

Cluster 6 Averege st. dev.: 0.5 0.452 0.439

Cluster nr. 7, composed of 31 TIP-OH2 heteroatoms, conservation 0.508:

(x y z iso_disp serial_number chain_identifier hetatm_type hetatom_name sequence_number PDB_id)

5.529 -10.332 5.738 0.0 78612 S TIP OH2 7706 frame0100

6.013 -10.295 6.05 0.0 79860 S TIP OH2 8122 frame0140

6.627 -9.547 5.607 0.0 73545 S TIP OH2 6017 frame0070

6.267 -9.515 5.332 0.0 66045 S TIP OH2 3517 frame0430

6.121 -9.814 5.715 0.0 71007 S TIP OH2 5171 frame0020

5.93 -10.702 5.267 0.0 75003 S TIP OH2 6503 frame0110

5.815 -9.33 5.906 0.0 78399 S TIP OH2 7635 frame0330

6.623 -10.654 4.655 0.0 54699 S TIP OH2 9735 frame0460

5.711 -11.118 5.056 0.0 59973 S TIP OH2 1493 frame0040

6.304 -10.818 5.498 0.0 63306 S TIP OH2 2604 frame0300

6.836 -9.62 5.233 0.0 63525 S TIP OH2 2677 frame0560

5.627 -9.475 6.224 0.0 55863 S TIP OH2 123 frame0280

5.807 -9.362 5.554 0.0 78474 S TIP OH2 7660 frame0530

6.264 -10.254 5.437 0.0 70014 S TIP OH2 4840 frame0170

6.532 -9.509 6.076 0.0 64980 S TIP OH2 3162 frame0060

6.414 -10.551 5.448 0.0 54987 S TIP OH2 9831 frame0310

5.223 -9.955 5.282 0.0 65577 S TIP OH2 3361 frame0380

5.97 -10.188 5.518 0.0 66852 S TIP OH2 3786 frame0290

6.11 -10.357 5.497 0.0 65202 S TIP OH2 3236 frame0180

6.099 -10.578 5.939 0.0 52332 S TIP OH2 8946 frame0320

6.412 -9.768 4.968 0.0 51219 S TIP OH2 8575 frame0030

6.016 -11.005 5.247 0.0 74121 S TIP OH2 6209 frame0210

5.489 -10.574 5.293 0.0 66306 S TIP OH2 3604 frame0480

5.558 -10.43 5.297 0.0 72888 S TIP OH2 5798 frame0050

6.074 -10.277 5.3 0.0 72240 S TIP OH2 5582 frame0200

6.313 -10.287 4.609 0.0 55818 S TIP OH2 108 frame0080

6.993 -9.774 5.908 0.0 52491 S TIP OH2 8999 frame0220

6.922 -10.55 5.488 0.0 69864 S TIP OH2 4790 frame0160

6.63 -10.096 5.905 0.0 64845 S TIP OH2 3117 frame0450

6.295 -10.474 5.182 0.0 73032 S TIP OH2 5846 frame0390

5.614 -11.275 5.984 0.0 58917 S TIP OH2 1141 frame0090

Cluster 7 Averege pos.: 6.133 -10.209 5.491

Cluster 7 Averege st. dev.: 0.439 0.523 0.389

Cluster nr. 8, composed of 16 TIP-OH2 heteroatoms, conservation 0.262:

(x y z iso_disp serial_number chain_identifier hetatm_type hetatom_name sequence_number PDB_id)

-0.763 -6.973 9.019 0.0 55593 S TIP OH2 33 frame0430

-0.862 -7.319 9.57 0.0 71619 S TIP OH2 5375 frame0020

-1.237 -7.194 10.056 0.0 60522 S TIP OH2 1676 frame0110

-1.125 -7.146 9.295 0.0 52824 S TIP OH2 9110 frame0520

-1.353 -6.692 9.9 0.0 53691 S TIP OH2 9399 frame0300

-1.572 -7.632 9.819 0.0 65058 S TIP OH2 3188 frame0540

-1.412 -7.096 8.554 0.0 70251 S TIP OH2 4919 frame0350

-1.125 -7.018 9.743 0.0 65091 S TIP OH2 3199 frame0120

-0.633 -6.895 9.825 0.0 52398 S TIP OH2 8968 frame0180

-1.68 -7.499 8.922 0.0 59154 S TIP OH2 1220 frame0030

-0.798 -6.754 9.437 0.0 66000 S TIP OH2 3502 frame0050

-0.721 -6.708 9.172 0.0 67740 S TIP OH2 4082 frame0220

-1.763 -7.157 9.366 0.0 73962 S TIP OH2 6156 frame0450

-1.692 -6.964 9.863 0.0 67128 S TIP OH2 3878 frame0250

-0.946 -7.339 9.92 0.0 67017 S TIP OH2 3841 frame0400

-0.729 -7.421 8.725 0.0 52539 S TIP OH2 9015 frame0470

Cluster 8 Averege pos.: -1.151 -7.113 9.449

Cluster 8 Averege st. dev.: 0.377 0.272 0.45

Cluster nr. 9, composed of 23 TIP-OH2 heteroatoms, conservation 0.377:

(x y z iso_disp serial_number chain_identifier hetatm_type hetatom_name sequence_number PDB_id)

7.447 0.401 9.179 0.0 62370 S TIP OH2 2292 frame0010

6.135 1.069 9.052 0.0 73719 S TIP OH2 6075 frame0340

6.227 1.004 9.162 0.0 73602 S TIP OH2 6036 frame0020

7.587 1.088 9.567 0.0 56082 S TIP OH2 196 frame0110

6.934 1.195 10.017 0.0 72903 S TIP OH2 5803 frame0330

7.161 1.153 8.661 0.0 62229 S TIP OH2 2245 frame0040

6.849 1.609 8.876 0.0 59874 S TIP OH2 1460 frame0300

6.969 0.763 8.636 0.0 65886 S TIP OH2 3464 frame0540

7.046 1.067 10.079 0.0 58044 S TIP OH2 850 frame0440

6.052 1.009 9.584 0.0 64989 S TIP OH2 3165 frame0270

7.17 1.143 8.842 0.0 71985 S TIP OH2 5497 frame0310

7.084 1.005 9.207 0.0 56451 S TIP OH2 319 frame0290

7.115 1.09 10.027 0.0 55194 S TIP OH2 9900 frame0410

7.497 0.515 9.736 0.0 58029 S TIP OH2 845 frame0480

6.537 1.227 9.805 0.0 57969 S TIP OH2 825 frame0050

6.46 1.435 8.736 0.0 74406 S TIP OH2 6304 frame0150

6.364 1.553 8.703 0.0 61254 S TIP OH2 1920 frame0420

7.126 1.008 8.39 0.0 62070 S TIP OH2 2192 frame0220

7.309 1.161 8.538 0.0 51249 S TIP OH2 8585 frame0510

6.131 1.276 9.147 0.0 76305 S TIP OH2 6937 frame0250

7.51 0.836 9.396 0.0 75645 S TIP OH2 6717 frame0390

7.135 1.772 9.448 0.0 70794 S TIP OH2 5100 frame0470

6.686 1.164 9.393 0.0 70711 S TIP OH2 1739 frame0000

Cluster 9 Averege pos.: 6.893 1.111 9.225

Cluster 9 Averege st. dev.: 0.468 0.305 0.494

Cluster nr. 10, composed of 16 TIP-OH2 heteroatoms, conservation 0.262:

(x y z iso_disp serial_number chain_identifier hetatm_type hetatom_name sequence_number PDB_id)

-0.047 2.203 2.653 0.0 57114 S TIP OH2 540 frame0490

-0.861 3.045 3.593 0.0 72126 S TIP OH2 5544 frame0590

0.095 2.907 2.805 0.0 66270 S TIP OH2 3592 frame0140

-0.737 2.569 3.623 0.0 54147 S TIP OH2 9551 frame0260

0.014 2.745 3.318 0.0 59325 S TIP OH2 1277 frame0460

-0.21 3.152 2.555 0.0 63954 S TIP OH2 2820 frame0520

-1.122 2.232 3.032 0.0 62013 S TIP OH2 2173 frame0300

-0.703 3.108 2.652 0.0 56427 S TIP OH2 311 frame0130

-0.093 3.33 3.099 0.0 67140 S TIP OH2 3882 frame0350

-0.978 2.938 3.304 0.0 70425 S TIP OH2 4977 frame0530

-0.215 2.616 3.539 0.0 75276 S TIP OH2 6594 frame0170

-0.361 2.509 2.714 0.0 66573 S TIP OH2 3693 frame0120

-0.602 2.698 3.024 0.0 77850 S TIP OH2 7452 frame0600

-0.046 2.139 2.678 0.0 62391 S TIP OH2 2299 frame0370

-1.164 2.086 2.793 0.0 64767 S TIP OH2 3091 frame0160

-1.167 3.107 3.054 0.0 64257 S TIP OH2 2921 frame0250

Cluster 10 Averege pos.: -0.512 2.712 3.027

Cluster 10 Averege st. dev.: 0.441 0.385 0.348

**Supplementary references**

1. Shabani S, Rabiei Z, Amini-Khoei H (2020) Exploring the multifaceted neuroprotective actions of gallic acid: a review. International Journal of Food Properties 23:736–752

2. Choi K-C, Lee Y-H, Jung MG, Kwon SH, Kim M-J, Jun WJ, Lee J, Lee JM, Yoon H-G (2009) Gallic Acid Suppresses Lipopolysaccharide-Induced Nuclear Factor-κB Signaling by Preventing RelA Acetylation in A549 Lung Cancer Cells. Molecular Cancer Research 7:2011–2021

3. Asokkumar K, Sen S, Umamaheswari M, Sivashanmugam AT, Subhadradevi V (2014) Synergistic effect of the combination of gallic acid and famotidine in protection of rat gastric mucosa. Pharmacological Reports 66:594–599

4. Ashrafizadeh M, Zarrabi A, Mirzaei S, et al (2021) Gallic acid for cancer therapy: Molecular mechanisms and boosting efficacy by nanoscopical delivery. Food Chem Toxicol 157:112576

5. Vaughan CK, Harryson P, Buckle AM, Fersht AR (2002) A structural double-mutant cycle: estimating the strength of a buried salt bridge in barnase. Acta Cryst D 58:591–600

6. Hendsch ZS, Tidor B (1994) Do salt bridges stabilize proteins? A continuum electrostatic analysis. Protein Science 3:211–226

7. Nayek A, Gupta PSS, Banerjee S, Mondal B, Bandyopadhyay AK (2014) Salt-Bridge Energetics in Halophilic Proteins. PLOS ONE 9:e93862

8. D. Vo T, L. Schneider A, David Wilson W, K. Poon GM (2021) Salt bridge dynamics in protein/DNA recognition: a comparative analysis of Elk1 and ETV6. Physical Chemistry Chemical Physics 23:13490–13502

9. Ekalu A, Habila JD (2020) Flavonoids: isolation, characterization, and health benefits. Beni-Suef University Journal of Basic and Applied Sciences 9:45

10. Tungmunnithum D, Thongboonyou A, Pholboon A, Yangsabai A (2018) Flavonoids and Other Phenolic Compounds from Medicinal Plants for Pharmaceutical and Medical Aspects: An Overview. Medicines (Basel) 5:93

11. Barreca D, Gattuso G, Bellocco E, Calderaro A, Trombetta D, Smeriglio A, Laganà G, Daglia M, Meneghini S, Nabavi SM (2017) Flavanones: Citrus phytochemical with health-promoting properties. BioFactors 43:495–506

12. Crowe-White KM, Evans LW, Kuhnle GGC, Milenkovic D, Stote K, Wallace T, Handu D, Senkus KE (2022) Flavan-3-ols and Cardiometabolic Health: First Ever Dietary Bioactive Guideline. Advances in Nutrition 13:2070–2083

13. Simons R, Gruppen H, H. Bovee TF, A. Verbruggen M, Vincken J-P (2012) Prenylated isoflavonoids from plants as selective estrogen receptor modulators (phytoSERMs). Food & Function 3:810–827

14. Dixon RA, Sumner LW (2003) Legume Natural Products: Understanding and Manipulating Complex Pathways for Human and Animal Health. Plant Physiology 131:878–885

15. Cianci M, Folli C, Zonta F, Florio P, Berni R, Zanotti G (2015) Structural evidence for asymmetric ligand binding to transthyretin. Acta Cryst D 71:1582–1592

16. Yokoyama T, Kosaka Y, Mizuguchi M (2015) Structural Insight into the Interactions between Death-Associated Protein Kinase 1 and Natural Flavonoids. J Med Chem 58:7400–7408

17. Yang L, Guo D, Fan C (2021) Identification and Structure–Activity Relationships of Dietary Flavonoids as Human Macrophage Migration Inhibitory Factor (MIF) Inhibitors. J Agric Food Chem 69:10138–10150

18. Nilsson L, Larsson A, Begum A, Iakovleva I, Carlsson M, Brännström K, Sauer-Eriksson AE, Olofsson A (2016) Modifications of the 7-Hydroxyl Group of the Transthyretin Ligand Luteolin Provide Mechanistic Insights into Its Binding Properties and High Plasma Specificity. PLOS ONE 11:e0153112

19. Gu C, Stashko MA, Puhl-Rubio AC, Chakraborty M, Chakraborty A, Frye SV, Pearce KH, Wang X, Shears SB, Wang H (2019) Inhibition of Inositol Polyphosphate Kinases by Quercetin and Related Flavonoids: A Structure–Activity Analysis. J Med Chem 62:1443–1454

20. Narwal M, Haikarainen T, Fallarero A, Vuorela PM, Lehtiö L (2013) Screening and Structural Analysis of Flavones Inhibiting Tankyrases. J Med Chem 56:3507–3517

21. Kriegel M, Wiederanders HJ, Alkhashrom S, Eichler J, Muller YA (2021) A PROSS-designed extensively mutated estrogen receptor α variant displays enhanced thermal stability while retaining native allosteric regulation and structure. Sci Rep 11:10509

22. Over B, Wetzel S, Grütter C, Nakai Y, Renner S, Rauh D, Waldmann H (2013) Natural-product-derived fragments for fragment-based ligand discovery. Nature Chem 5:21–28

23. Trivella DBB, dos Reis CV, Lima LMTR, Foguel D, Polikarpov I (2012) Flavonoid interactions with human transthyretin: Combined structural and thermodynamic analysis. Journal of Structural Biology 180:143–153

24. Reiberger R, Radilová K, Kráľ M, Zima V, Majer P, Brynda J, Dračínský M, Konvalinka J, Kožíšek M, Machara A (2021) Synthesis and In Vitro Evaluation of C-7 and C-8 Luteolin Derivatives as Influenza Endonuclease Inhibitors. International Journal of Molecular Sciences 22:7735

25. Shuab R, Lone R, Koul KK (2016) Cinnamate and cinnamate derivatives in plants. Acta Physiol Plant 38:64

26. Adisakwattana S (2017) Cinnamic Acid and Its Derivatives: Mechanisms for Prevention and Management of Diabetes and Its Complications. Nutrients 9:163

27. Ruwizhi N, Aderibigbe BA (2020) Cinnamic Acid Derivatives and Their Biological Efficacy. International Journal of Molecular Sciences 21:5712

28. Aijaz M, Keserwani N, Yusuf M, Ansari NH, Ushal R, Kalia P (2022) Chemical, biological, and pharmacological prospects of caffeic acid. Biointerface Res Appl Chem 13:324

29. Meng S, Cao J, Feng Q, Peng J, Hu Y (2013) Roles of Chlorogenic Acid on Regulating Glucose and Lipids Metabolism: A Review. Evid Based Complement Alternat Med 2013:801457

30. Xue N, Liu Y, Jin J, Ji M, Chen X (2022) Chlorogenic Acid Prevents UVA-Induced Skin Photoaging through Regulating Collagen Metabolism and Apoptosis in Human Dermal Fibroblasts. Int J Mol Sci 23:6941

31. Nunes S, Madureira AR, Campos D, Sarmento B, Gomes AM, Pintado M, Reis F (2017) Therapeutic and nutraceutical potential of rosmarinic acid—Cytoprotective properties and pharmacokinetic profile. Critical Reviews in Food Science and Nutrition 57:1799–1806

32. Noor S, Mohammad T, Rub MA, Raza A, Azum N, Yadav DK, Hassan MI, Asiri AM (2022) Biomedical features and therapeutic potential of rosmarinic acid. Arch Pharm Res 45:205–228

33. Ma Z, Lu Y, Yang F, Li S, He X, Gao Y, Zhang G, Ren E, Wang Y, Kang X (2020) Rosmarinic acid exerts a neuroprotective effect on spinal cord injury by suppressing oxidative stress and inflammation via modulating the Nrf2/HO-1 and TLR4/NF-κB pathways. Toxicology and Applied Pharmacology 397:115014

34. Cozza G, Zonta F, Dalle Vedove A, Venerando A, Dall’Acqua S, Battistutta R, Ruzzene M, Lolli G (2020) Biochemical and cellular mechanism of protein kinase CK2 inhibition by deceptive curcumin. The FEBS Journal 287:1850–1864

35. Rajput JD, Bagul SD, Pete UD, Zade CM, Padhye SB, Bendre RS (2018) Perspectives on medicinal properties of natural phenolic monoterpenoids and their hybrids. Mol Divers 22:225–245

36. Mazzei L, Cianci M, Musiani F, Lente G, Palombo M, Ciurli S (2017) Inactivation of urease by catechol: Kinetics and structure. Journal of Inorganic Biochemistry 166:182–189

37. Zhang L-X, Li C-X, Kakar MU, et al (2021) Resveratrol (RV): A pharmacological review and call for further research. Biomedicine & Pharmacotherapy 143:112164

38. Su X, Zhou D, Li N (2022) Chapter 8 - Bioactive stilbenes from plants. In: Atta-ur-Rahman (ed) Studies in Natural Products Chemistry. Elsevier, pp 265–403

39. Neves AR, Lúcio M, Lima JLC, Reis S (2012) Resveratrol in Medicinal Chemistry: A Critical Review of its Pharmacokinetics, Drug-Delivery, and Membrane Interactions. Current Medicinal Chemistry 19:1663–1681

40. Buryanovskyy L, Fu Y, Boyd M, Ma Y, Hsieh T, Wu JM, Zhang Z (2004) Crystal Structure of Quinone Reductase 2 in Complex with Resveratrol,. Biochemistry 43:11417–11426

41. Sharifi-Rad J, Cruz-Martins N, López-Jornet P, et al (2021) Natural Coumarins: Exploring the Pharmacological Complexity and Underlying Molecular Mechanisms. Oxid Med Cell Longev 2021:6492346

42. Poumale HMP, Hamm R, Zang Y, Shiono Y, Kuete V (2013) 8 - Coumarins and Related Compounds from the Medicinal Plants of Africa. In: Kuete V (ed) Medicinal Plant Research in Africa. Elsevier, Oxford, pp 261–300

43. Schuller M, Correy GJ, Gahbauer S, et al (2021) Fragment binding to the Nsp3 macrodomain of SARS-CoV-2 identified through crystallographic screening and computational docking. Sci Adv 7:eabf8711

44. Tu Y, Yang Y, Li Y, He C (2021) Naturally occurring coumestans from plants, their biological activities and therapeutic effects on human diseases. Pharmacological Research 169:105615

45. Kuiper GGJM, Carlsson B, Grandien K, Enmark E, Häggblad J, Nilsson S, Gustafsson J-Å (1997) Comparison of the Ligand Binding Specificity and Transcript Tissue Distribution of Estrogen Receptors α and β. Endocrinology 138:863–870

46. Blomquist CH, Lima PH, Hotchkiss JR (2005) Inhibition of 3α-hydroxysteroid dehydrogenase (3α-HSD) activity of human lung microsomes by genistein, daidzein, coumestrol and C18-, C19- and C21-hydroxysteroids and ketosteroids. Steroids 70:507–514

47. Battistutta R, Lolli G (2019) Inhibitory Properties of ATP-Competitive Coumestrol and Boldine Are Correlated to Different Modulations of CK2 Flexibility. J Nat Prod 82:1014–1018

48. Yeung AWK, Tzvetkov NT, Balacheva AA, et al (2020) Lignans: Quantitative Analysis of the Research Literature. Frontiers in Pharmacology 11:

49. Milder IEJ, Arts ICW, Putte B van de, Venema DP, Hollman PCH (2005) Lignan contents of Dutch plant foods: a database including lariciresinol, pinoresinol, secoisolariciresinol and matairesinol. British Journal of Nutrition 93:393–402

50. Owen RW, Giacosa A, Hull WE, Haubner R, Spiegelhalder B, Bartsch H (2000) The antioxidant/anticancer potential of phenolic compounds isolated from olive oil. European Journal of Cancer 36:1235–1247

51. Wikul A, Damsud T, Kataoka K, Phuwapraisirisan P (2012) (+)-Pinoresinol is a putative hypoglycemic agent in defatted sesame (Sesamum indicum) seeds though inhibiting α-glucosidase. Bioorganic & Medicinal Chemistry Letters 22:5215–5217

52. López-Biedma A, Sánchez-Quesada C, Beltrán G, Delgado-Rodríguez M, Gaforio JJ (2016) Phytoestrogen (+)-pinoresinol exerts antitumor activity in breast cancer cells with different oestrogen receptor statuses. BMC Complement Altern Med 16:350

53. López-Biedma A, Sánchez-Quesada C, Delgado-Rodríguez M, Gaforio JJ (2016) The biological activities of natural lignans from olives and virgin olive oils: A review. Journal of Functional Foods 26:36–47

54. Lei S, Wu S, Wang G, Li B, Liu B, Lei X (2021) Pinoresinol diglucoside attenuates neuroinflammation, apoptosis and oxidative stress in a mice model with Alzheimer’s disease. NeuroReport 32:259

55. Xiao Y, Shao K, Zhou J, et al (2021) Structure-based engineering of substrate specificity for pinoresinol-lariciresinol reductases. Nat Commun 12:2828

56. Hook I, Mills C, Sheridan H (2014) Chapter 5 - Bioactive Naphthoquinones from Higher Plants. In: Atta-ur-Rahman (ed) Studies in Natural Products Chemistry. Elsevier, pp 119–160

57. Ahmad T, Suzuki YJ (2019) Juglone in Oxidative Stress and Cell Signaling. Antioxidants 8:91

58. Hu Q, Yang C, Zheng F, Duan H, Fu Y, Cheng Z (2020) Acute lung injury inhibition by juglone in LPS induced sepsis mouse model involves Sirt1 activation. Tropical Journal of Pharmaceutical Research 19:1001–1007

59. O. Salas C, Faundez M, Morello A, Diego Maya J, A. Tapia R (2011) Natural and Synthetic Naphthoquinones Active Against Trypanosoma Cruzi: An Initial Step Towards New Drugs for Chagas Disease. Current Medicinal Chemistry 18:144–161

60. Meyers NW, Karasik A, Kaitany K, Fierke CA, Koutmos M (2022) Gambogic acid and juglone inhibit RNase P through distinct mechanisms. Journal of Biological Chemistry. https://doi.org/10.1016/j.jbc.2022.102683

61. Hewlings SJ, Kalman DS (2017) Curcumin: A Review of Its’ Effects on Human Health. Foods 6:92

62. Fuloria S, Mehta J, Chandel A, et al (2022) A Comprehensive Review on the Therapeutic Potential of Curcuma longa Linn. in Relation to its Major Active Constituent Curcumin. Frontiers in Pharmacology 13:

63. Manolova Y, Deneva V, Antonov L, Drakalska E, Momekova D, Lambov N (2014) The effect of the water on the curcumin tautomerism: A quantitative approach. Spectrochimica Acta Part A: Molecular and Biomolecular Spectroscopy 132:815–820

64. Bank RPD RCSB PDB - 6HDR: Human DYRK2 bound to Curcumin. https://www.rcsb.org/structure/6HDR. Accessed 9 Feb 2024

65. Ciccone L, Tepshi L, Nencetti S, Stura EA (2015) Transthyretin complexes with curcumin and bromo-estradiol: evaluation of solubilizing multicomponent mixtures. New Biotechnology 32:54–64

66. Zhuang C, Zhang W, Sheng C, Zhang W, Xing C, Miao Z (2017) Chalcone: A Privileged Structure in Medicinal Chemistry. Chem Rev 117:7762–7810

67. Salehi B, Quispe C, Chamkhi I, et al (2021) Pharmacological Properties of Chalcones: A Review of Preclinical Including Molecular Mechanisms and Clinical Evidence. Frontiers in Pharmacology 11:

68. WalyEldeen AA, Sabet S, El-Shorbagy HM, Abdelhamid IA, Ibrahim SA (2023) Chalcones: Promising therapeutic agents targeting key players and signaling pathways regulating the hallmarks of cancer. Chemico-Biological Interactions 369:110297

69. Antoniolli G, Almeida WP, Frias CC, de Oliveira TB (2021) Chalcones Acting as Inhibitors of Cholinesterases, β-Secretase and β- Amyloid Aggregation and other Targets for Alzheimer’s Disease: A Critical Review. Current Medicinal Chemistry 28:4259–4282

70. Orlikova B, Tasdemir D, Golais F, Dicato M, Diederich M (2011) Dietary chalcones with chemopreventive and chemotherapeutic potential. Genes Nutr 6:125–147

71. Songyang Y, Li W, Li W, Yang J, Song T (2022) The inhibition of GLUT1-induced glycolysis in macrophage by phloretin participates in the protection during acute lung injury. International Immunopharmacology 110:109049

72. Song HS, Jang S, Kang SC (2018) Bavachalcone from Cullen corylifolium induces apoptosis and autophagy in HepG2 cells. Phytomedicine 40:37–47

73. Zubieta C, He X-Z, Dixon RA, Noel JP (2001) Structures of two natural product methyltransferases reveal the basis for substrate specificity in plant O-methyltransferases. Nat Struct Mol Biol 8:271–279

74. Sharifi-Rad J, Quispe C, Castillo CMS, et al (2022) Ellagic Acid: A Review on Its Natural Sources, Chemical Stability, and Therapeutic Potential. Oxidative Medicine and Cellular Longevity 2022:e3848084

75. García-Niño WR, Zazueta C (2015) Ellagic acid: Pharmacological activities and molecular mechanisms involved in liver protection. Pharmacological Research 97:84–103

76. Tošović J, Bren U (2020) Antioxidative Action of Ellagic Acid—A Kinetic DFT Study. Antioxidants 9:587

77. Brittain HG (2002) Mandelic Acid. In: Brittain HG (ed) Analytical Profiles of Drug Substances and Excipients. Academic Press, pp 179–211

78. Kallarakal AT, Mitra B, Kozarich JW, Gerlt JA, Clifton JG, Petsko GA, Kenyon GL (1995) Mechanism of the reaction catalyzed by mandelate racemase: structure and mechanistic properties of the K166R mutant. Biochemistry 34:2788–2797

79. Li C, Li M, Chen P, Narayan S, Matschinsky FM, Bennett MJ, Stanley CA, Smith TJ (2011) Green Tea Polyphenols Control Dysregulated Glutamate Dehydrogenase in Transgenic Mice by Hijacking the ADP Activation Site. J Biol Chem 286:34164–34174
